# Assessing the reliability of medicinal *Dendrobium* sequences in GenBank for botanical species identification

**DOI:** 10.1038/s41598-021-82385-z

**Published:** 2021-02-09

**Authors:** Hoi-Yan Wu, Kwun-Tin Chan, Grace Wing-Chiu But, Pang-Chui Shaw

**Affiliations:** 1 Li Dak Sum Yip Yio Chin R&D Centre for Chinese Medicine, The Chinese University of Hong Kong, Hong Kong, China; 2 State Key Laboratory of Research on Bioactivities and Clinical Applications of Medicinal Plants (The Chinese University of Hong Kong) and Institute of Chinese Medicine, The Chinese University of Hong Kong, Hong Kong, China; 3 School of Life Sciences, The Chinese University of Hong Kong, Hong Kong, China

**Keywords:** Biological techniques, Genetic techniques

## Abstract

DNA-based method is a promising tool in species identification and is widely used in various fields. DNA barcoding method has already been included in different pharmacopoeias for identification of medicinal materials or botanicals. Accuracy and validity of DNA-based methods rely on the accuracy and taxonomic reliability of the DNA sequences in the database to be compared against. Here we evaluated the annotation quality and taxonomic reliability of selected barcode loci (rbcL, matK, psbA-trnH, trnL-trnF and ITS) of 41 medicinal *Dendrobium* species downloaded from GenBank. Annotations of most accessions are incomplete. Only 53.06% of the 2041 accessions downloaded contain a reference to a voucher specimen. Only 31.60% and 4.8% of the entries are annotated with country of origin and collector or assessor, respectively. Taxonomic reliability of the sequences was evaluated by a Megablast search based on similarity to sequences submitted by other research groups. A small number of sequences (211, 7.14%) was regarded as highly doubted. Moreover, 10 out of 60 complete chloroplast genomes contain highly doubted sequences. Our findings suggest that sequences of GenBank should be used with caution for species-level identification. The scientific community should provide more important information regarding identity and traceability of the sample when they deposit sequences to public databases.

## Introduction

DNA-based identification techniques have gained extensive popularity in the last two decades. They have been widely adopted in various applications, such as biodiversity assessment^1^, monitoring CITES-listed plants and animals^2,3^, detecting food fraud^4^, and authenticating medicinal materials or raw materials in botanical dietary supplements^5,6^. DNA barcoding method has already been included in guiding principle of the Chinese Pharmacopoeia, general chapter of the United States Pharmacopoeia and supplementary chapter of the British Pharmacopoeia. However, reference DNA barcode sequences of most medicinal materials and their common substitutes or closely related species are still unavailable. While new molecular identification techniques keep coming out, especially for pharmacovigilance of medicinal herbs or dietary supplements by next-generation sequencing, efforts in ensuring the taxonomic reliability of sequences in public databases are not on par. Up to 2019, only a handful of research articles have been published on assessing the reliability on taxonomic identity and annotation of DNA sequences in public databases including EMBL sequence database and GenBank. Two studies focused on internal transcribed spacer (ITS) sequences of fungi and their conclusions are similarly discouraging: up to 20% of the sequences investigated might be unreliable or incorrectly identified up to species level^7,8^. Nilsson’s study also revealed a serious insufficiency in annotations of entries, with 82% of the sequences lacking explicit reference to voucher specimen and only 2% of the sequences having information on collector or determinator. Longo et al. conducted a search for sequences of human origin in non-human species, and found that ten NCBI genome assemblies were contaminated with human sequences (10.64%)^9^. Renata et al. analysed the identity of 105 GenBank accessions of ITS2 and 138 COI sequences deposited as *Tetranychus*, and estimated that nearly 30% of the sequences were misidentified or dubious sequences^10^.

In 2019, two new studies on reliability of sequences in public databases were published. The reliability of metazoan mitochondrial sequences in GenBank was evaluated by clustering sequences of 15 mitochondrial loci at 97–100% thresholds^11^. The percentage of mislabelled metazoan mitochondrial sequences at the genus level in multi-sequence clusters containing multiple genera was estimated to be 0.67–3.22%. The low error rate is encouraging, providing another strong piece of evidence supporting the use of DNA-based identification with mitochondrial sequences in animals. Meiklejohn et al. assessed the accuracy and reliability of barcode sequences of selected insects, macro-fungi and plants in the Barcode of Life DataSystems (BOLD) and GenBank, by generating relevant barcode sequences from curated reference materials and subjecting the reference sequences to identification by BOLD or by NCBI BLAST^12^. When using 2-loci barcode for plants (rbcL and matK), discrimination power at the genus and species level was about 91% and 80%, respectively, in both BOLD and GenBank. ITS sequences provided 100% accurate genus assignment and 57% correct identification of selected macro-fungi taxa. For insect taxa, the rate of accurate species-level identification was very low in both databases, with 53% for GenBank and 35% for BOLD. The low accuracy in insect identification has raised some doubts in the scientific community. Pentinsaari et al. later revisited the sequence data and samples investigated by Meiklejohn et al. and found that the rate of correct species identification should be 9/13 in both BOLD and GenBank, after resolving some missteps taken in the original study^13^. Also, the method of assessment employed by Meiklejohn et al. only shows the accuracy of taxa identification based on searching against the two databases. Misidentified or mis-annotated sequences with low similarity to the query sequence cannot be called out by the search algorithms. The number and extent of such sequences could not be revealed by their assessment. Presence of misidentified or insufficiently annotated sequences in public database would hamper the reliability of taxonomic identification inferred from such sequences, especially for species with sequences deposited by only one or few research groups.

Species level identification is essential for ensuring authenticity of herbal medicinal materials and law enforcement. Here, we attempted to evaluate the comprehensiveness of annotation and estimate the reliability taxonomic of standard barcodes and supplementary barcodes deposited in GenBank of 41 medicinal *Dendrobium* species^14^. There are 74 species and 2 varieties of *Dendrobium* in China and a lot of them have been used medicinally. *Dendrobium* was documented as a “superior grade” herb in “Shen Nong’s Herbal Classic”, the oldest surviving text on Chinese materia medica. It is well known for its multifaceted pharmacological effects, including nourishing the Yin, moisturising the lung, supplementing the stomach, promoting production of body fluids, and clearing heat^14,15^. Modern scientific research showed that polysaccharides and other compounds of *Dendrobium* have immunomodulatory effects, hepatoprotective activity, neuroprotective effects, anticancer activities and hypoglycemic effect^16,17^ *Dendrobium*, as an orchid genus, produces beautiful flowers and is popular in horticulture. *Dendrobium* species are notoriously difficult to identify macroscopically because of their similar morphological appearance and tissue structure, especially when they are in the form of dried herbs^18–20^. The abundance of closely-related species that are difficult to differentiate morphologically makes the sequences of *Dendrobium* in GenBank susceptible to mis-identification and/or mis-annotation. Previous comparative analysis of complete plastomes revealed diverse intraspecific sequence variability in plastomic mutational hotspots among different *Dendrobium* species^20^. The similarity among congeneric *Dendrobium* species makes it less easy to spot any mis-identifications based on sequence similarity by the groups depositing the sequences to the public database. It would be interesting to know how reliable the sequences of medicinal *Dendrobium* in GenBank are at species level. The evaluation results of species as complicated and as closely-related as *Dendrobium* should give us a good overall picture of validity of DNA barcode sequences in GenBank for species-level authentication of medicinal herbs.

## Materials and methods

### Sequences retrieved from GenBank

Standard barcodes (rbcL and matK) and supplementary barcodes (psbA-trnH intergenic spacer, trnL-trnF intergenic spacer and ITS) of 41 *Dendrobium* species (Table 1) listed as medicinally used by Cheng et al.^14^ were downloaded from GenBank during Jan 2019–May 2019. To ensure consistency and thoroughness, NCBI taxonomy ID of each species, together with gene name, was used to search for sequences in NCBI Nucleotide database. Sequences and data in the form of INSDSeq eXtensible Markup Language (XML) files were downloaded from GenBank. An XSLT script was used to extract and categorize data from the XML files for annotation assessment. Irrelevant sequences were removed after manual screening. Complete chloroplast genomes of the target species were also downloaded by the same means. Barcodes within complete chloroplast genomes were retrieved by manually extracting the sequences according to gene annotation shown in the accession. For trnL-trnF intergenic spacer from complete chloroplast genome, the extracted sequence spans from the nucleotide immediately after trnL-UAA to the nucleotide immediately before trnF-GAA. For psbA-trnH intergenic spacer, the sequence was obtained by joining the fragment from the nucleotide immediately after trnH-GUG to the last nucleotide of the accession, and the fragment from the first nucleotide of the accession to the nucleotide immediately before psbA gene together.

**Table 1:** List of 41 medicinal *Dendrobium* species^14^ included in this study and the number of accessions evaluated.

Species	rbcL	matK	psbA-trnH	trnL-trnF	trnL^a^	ITS	ITS2
*D. aduncum* Lindl	8	9	9	0	2	15	5
*D. aphyllum* (Roxb.) C. E. C. Fisch	22	22	4	1	1	29	4
*D. aurantiacum* Rchb. f. var. *denneanum* (Kerr) Z.H.Tsi	4	9	57	1	3	15	3
*D. brymerianum* Rchb. f	4	9	6	1	2	12	2
*D. capillipes* Rchb. f	2	8	2	1	1	8	3
*D. chrysanthum* Wall. & Lindl	16	21	7	0	1	31	0
*D. chrysotoxum* Lindl	8	13	10	1	2	20	6
*D. crepidatum* Lindl. & Paxton	15	32	11	1	2	38	4
*D. crystallinum* Rchb. f	6	8	4	1	1	9	2
*D. densiflorum* Lindl	10	12	6	1	2	18	6
*D. devonianum* Paxton	10	12	7	1	2	17	4
*D. falconeri* Hook. f	7	9	5	1	2	15	2
*D. fimbriatum* Hook. f	12	16	4	1	1	19	4
*D. flexicaule* Z. H. Tsi, S. C. Sun & L. G. Xu	0	0	3	0	2	7	0
*D. gibsonii* Lindl	1	1	0	1	0	4	2
*D. gratiosissimum* Rchb. f	4	7	5	0	4	13	1
*D. hancockii* Rolfe	3	9	6	1	1	13	5
*D. harveyanum* Rchb. f	3	6	3	0	3	7	1
*D. hercoglossum* Rchb. f	4	9	7	1	5	16	4
*D. heterocarpum* Wall. & Lindl	7	8	5	1	1	12	1
*D. huoshanense* C. Z. Tang & S. J. Cheng	4	5	5	0	3	18	0
*D. jenkinsii* Wall. & Lindl	8	13	5	1	3	19	4
*D. lindleyi* Steud	6	13	3	0	1	15	6
*D. lituiflorum* Lindl	2	7	2	0	0	5	2
*D. loddigesii* Rolfe	6	9	5	1	3	11	5
*D. minutiflorum* S. C. Chen & Z. H. Tsi	2	2	2	0	2	4	0
*D. moniliforme* (L.) Sw	37	39	42	1	3	32	5
*D. monticola* P.F.Hunt & Summerh	0	0	0	0		2	0
*D. moschatum* (Buch.-Ham.) Sw	10	14	1	0	1	17	2
*D. nobile* Lindl	17	33	22	21	5	71	11
*D. officinale* Kimura & Migo	3	14	23	1	0	82	21
*D. parciflorum* Rchb. f. & Lindl	0	1	0	0	0	2	0
*D. parishii* Rchb. f	3	7	0	0	1	10	1
*D. pendulum* Roxb	5	7	5	0	4	14	3
*D. primulinum* Lindl	12	18	5	1	2	21	4
*D. scoriarum* W.W.Sm	1	1	4	0	2	11	3
*D. strongylanthum* Rchb. f	3	3	3	0	4	12	1
*D. thyrsiflorum* Rchb. f	5	7	6	0	1	15	7
*D. tosaense* Makino	2	2	3	6	1	17	0
*D. wardianum* Warner	5	7	5	1	0	12	3
*D. willsonii* Rolfe	2	3	3	0	1	4	0

^a^Downloaded from GenBank as trnL-trnF but later found to be trnL gene.

### Evaluation of accession annotation and taxonomic reliability

Validity of DNA-based identification methods rely on a comprehensive reference sequence database. Reference sequence selected should be sequence generated from voucher specimen that are collected or morphologically determined by an experienced taxonomist, and with clear record of geographic origin. To evaluate the quality of annotation on sequence traceability, availability of information on (1) voucher specimen number, (2) country of origin and (3) collector or assessor in FEATURES field and publication status of GenBank accession was recorded. To confirm if the accession has actually been published and to find out if there would be additional information on the three criteria of sequence traceability from the published articles, we used the GenBank accession number, AUTHORS and (article or project) TITLE fields of the accession to search for English and Chinese publications in Pubmed, Google Scholar, China Journal Net and Wanfang Data platform.

Taxonomic reliability of the extracted sequences was estimated by a versus-all search in NCBI Nucleotide collection (nr/nt) by Megablast. Taxonomic identity of a query sequence would be regarded as not-doubted if the query sequence has ≥ 99% similarity to sequences of the declared species deposited by ≥ 2 groups of researchers. Query sequences 97.0–98.9% similar to sequences of declared species are classified as doubted, if the declared species is (one of) the best-matched species in BLAST. Sequences that are best matched to another species with < 99% similarity to sequence of declared species and sequences having < 97% similarity to sequence of declared species are regarded as highly doubted. Query sequences would not be assessable if the sequences of a certain species had been deposited by one research group only. In addition to taxonomic identity, accuracy of gene or loci labelling would also be noted.

### Estimation of species discriminatory power by phylogenetic tree analysis

Barcode sequences of the *Dendrobium* species which are not listed in Table 1 were also downloaded from GenBank, with a sequence length filter ranging from 300 to 4000 bp to limit the number and length of sequences to a manageable scale. These downloaded sequences were aligned with the evaluated sequences of the 41 *Dendrobium* species, with the highly doubted sequences excluded, by MAFFT version 7 online^21^. Barcoding gap analysis was performed by computing the maximum intraspecific distance and minimum interspecific distance of each sequence using Species Identifier 1.8 in TaxonDNA^22^. For each of the 41 *Dendrobium* species, maximum intraspecific distance was plotted against minimum distance to the nearest congeneric species for each region. Uncorrected p-distance was chosen and the sequences should have at least 300 bp in common. Neighbor-joining trees were constructed for each region using Kimura 2-parameter (K2P) model with 1000 bootstrap replicates using MEGA 7.0^23^. Gaps and missing data treatment were selected as partial deletion with 90% site coverage cut-off. Sequences of *Liparis kumokiri* were also downloaded from GenBank and aligned together as outgroup.

## Results

### Incompleteness of annotation for traceability

A total of 2041 accessions were downloaded from GenBank. Table 2 summarises the results of assessment of annotations. The availability of information vital for traceability and verification is far from satisfactory. It was found that 53.06% of the entries contain reference to a voucher specimen number in the GenBank record. Only 31.60% of the sequences have the country of origin marked. The number of entries carrying information on the collector or assessor is alarmingly low, with only 98 accessions (4.80%). However, some authors would include these three pieces of information in the publication, but not the GenBank record. When information from the publications were also taken into account, there would be 59.97% accessions with country of origin annotated, which almost doubles the percentage in GenBank records. The identity of the collector or assessor was also more commonly mentioned in publications, resulting a total of 18.19% accessions.

**Table 2:** Summary of assessment of annotations.

Number of *Dendrobium* species	41
Total number of accessions evaluated	2041
**Number of accessions**	
With voucher specimen in GenBank	1083 (53.06%)
With country specified in GenBank	645 (31.60%)
With collector/determinator specified in GenBank	98 (4.80%)
With additional information on voucher specimen in publication	178 (8.72%)
With additional information on country in publication	579 (28.37%)
With additional information on collector/determinator in publication	272 (13.33%)
Annotated as published in GenBank	1030 (50.47%)
Published but not annotated as so in GenBank	222 (10.88%)
Annotated as published in GenBank but could not be explicitly found in the annotated publication	41 (2.01%)
Of which voucher specimen number in GenBank record is not the same as the number in publication	8 (0.39%)

There are 1030 (50.47%) accessions marked as published in GenBank. But our literature search found out that 222 (10.88%) additional sequences, listed as unpublished sequences, have been published. These publications were not updated in the GenBank record. Interestingly, 2.01% of the sequences were annotated as published with citation of research articles, but they were not mentioned in their corresponding publications.

### Estimation of taxonomic reliability

In this study, we tried to estimate the taxonomic reliability of the barcode and supplementary barcode sequences in GenBank based on the similarity of the query sequences to other sequences of the declared species submitted by another research group in GenBank by a versus-all Megablast search. Standard barcodes, rbcL and matK, and supplementary barcodes, psbA-trnH, trnL and trnL-trnF, were extracted from chloroplast complete genomes and analysed individually. The same also applied to ITS1 and ITS2 from full-length ITS sequences. Among the 2984 sequences evaluated, 89.78% of them were classified as not-doubted while 7.05% of them were regarded as highly doubted (Table 3). During the BLAST search, we revealed an unexpected annotation error commonly made by different contributors. Out of 123 “trnL-trnF intergenic spacer” sequences studied, 75 sequences (60.98%) actually belong to trnL intron only, but not trnL-trnF.

**Table 3:** Summary of taxonomic reliability of all sequences retrieved.

Total number of blasted sequences	2993
Number of highly doubted sequences	211 (7.05%)
Number of doubted sequences	95 (3.17%)
Total number of complete chloroplast genomes	60
Total number of doubted chloroplast genomes	10 (16.67%)

Looking into the results of each region individually, ITS1 and ITS2 regions are the most contributed (Table 4). There were ITS1 sequences of all 41 *Dendrobium* species in GenBank, and the number of ITS2 sequences was the highest among the seven regions studied. The trnL-trnF intergenic spacer is the least contributed sequence, with only 108 sequences covering 33 *Dendrobium* species, especially after we re-classified the sequences containing trnL only for our analysis. The trnL intron has the highest proportion of highly doubted sequences at 21.48%, which is more than double of ITS1, the region with the second highest proportion of highly doubted sequence (8.99%). The rbcL and matK have the least highly doubted sequences, at 1.77% and 1.44%, respectively. There are 48 sequences classified as “not-assessable” because their contributors are the only research groups submitting sequences of the particular barcode regions of the declared species. Most of them belong to trnL-trnF, of which 20% of the sequences could not be assessed by our method. It was also found that 10 out of 60 complete chloroplast genomes evaluated contain highly doubted sequences, and nine of them have been adopted as provisional refseq by NCBI. All accessions regarded as “highly doubted” were listed in Table S1 with reasons of doubt provided. Among 211 highly doubted sequences, 149 were best match to another *Dendrobium* species and 11 were best match to a totally different genus.

**Table 4:** Tally of doubted sequences for each barcode locus.

	rbcL	matK	psbA-trnH	trnL-trnF	trnL intron	ITS1	ITS2
Number of species with this region	40	40	39	33	39	41	40
Number of blasted sequences	339	485	365	108	135	712	849
Number of highly doubted sequences	6 (1.77%)	7 (1.44%)	31 (8.49%)	7 (6.48%)	29 (21.48%)	64 (8.99%)	67 (7.89%)
Number of doubted sequences	0	4 (0.82%)	10 (2.74%)	5 (4.63%)	26 (19.26%)	20 (2.81%)	29 (3.42%)

### Differentiation power of individual locus at species level

**Figure 1:**
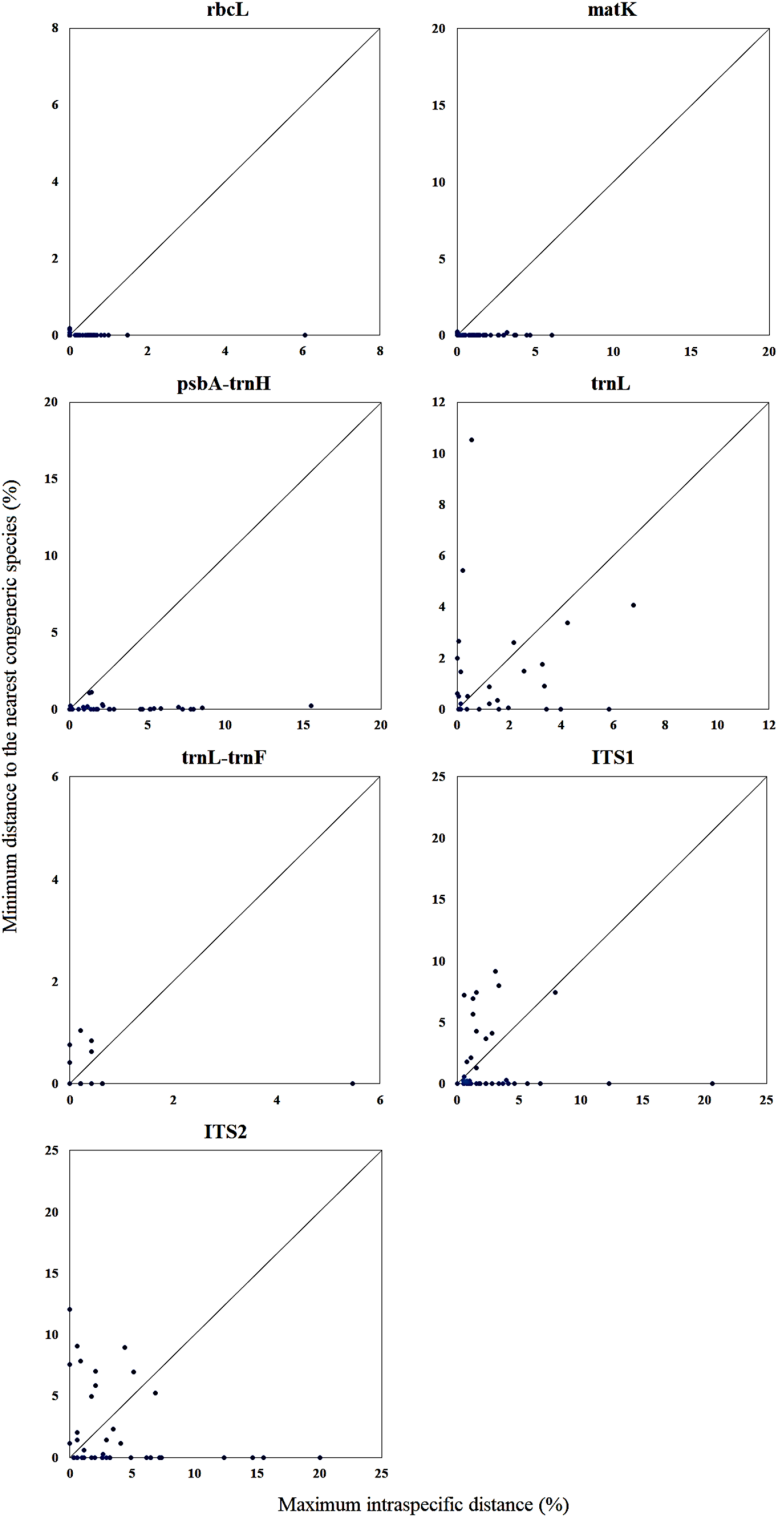
Barcode gap plot for the seven individual barcodes. The distances to the nearest congeneric species vs. the maximum intraspecific distances (%) were plotted for species discrimination. Each dot represents one species. Dots above the 1:1 line indicated the presence of a barcode gap.

Estimation of sequence reliability based on its similarity to other sequences of the same species could not indicate the differentiation power of a sequence. Ambiguous match results are common in BLAST, when more than one species tied for the top match with the same max score, i.e. the highest bit-score. To shed light on the discrimination ability of the not-doubted sequences of the 41 *Dendrobium* species, distance-based barcoding gap analysis and phylogenetic tree analysis were performed. The performance of the seven single barcode loci in species discrimination is shown in Table 5 and Fig. 1. Neighbor-joining trees of the seven loci are shown in Figs. S1–S7. Using not-doubted sequences of the 41 *Dendrobium* species and non-curated, downloaded sequences of other *Dendrobium* species, ITS1 and ITS2 gave the highest rate of species discrimination in barcoding gap analysis, with 12 out of 41 species (29.27%) showing distinct barcoding gaps. In tree-based analysis, ITS2 resolved the highest number of species, i.e. same species clustered in a monophyletic clade (14 out of 41 species, 34.15%), followed by ITS1 (13 out of 41 species, 31.71%) and trnL gene (7 out of 41 species, 17.07%).

**Table 5:** *Dendrobium* species identifiable using the single barcode based on barcoding gap analysis and tree-based analysis.

rbcL	matK	psbA-trnH	trnL	trnL-trnF	ITS1	ITS2
**Barcoding gap analysis**						
*D. harveyanum*	*D. flexicaule*	*D. harveyanum*	*D. aduncum*	*D. aphyllum*	*D. aduncum*	*D. brymerianum*
*D. minutiflorum*	*D. minutiflorum*		*D. brymerianum*	*D. brymerianum*	*D. brymerianum*	*D. chrysanthum*
*D. scoriarum*	*D. scoriarum*		*D. crepidatum*	*D. devonianum*	*D. chrysanthum*	*D. chrysotoxum*
*D. thyrsiflorum*			*D. crystallinum*	*D. fimbriatum*	*D. chrysotoxum*	*D. crystallinum*
			*D. falconeri*	*D. scoriarum*	*D. crystallinum*	*D. devonianum*
			*D. flexicaule*		*D. devonianum*	*D. gibsonii*
			*D. gratiosissimum*		*D. gibsonii*	*D. harveyanum*
			*D. heterocarpum*		*D. gratiosissimum*	*D. heterocarpum*
			*D. minutiflorum*		*D. harveyanum*	*D. jenkinsii*
			*D. scoriarum*		*D. jenkinsii*	*D. loddigesii*
					*D. loddigesii*	*D. minutiflorum*
					*D. pendulum*	*D. pendulum*
**Neighbor joining tree analysis**						
*D. scoriarum*	*D. capillipes*	*D. harveyanum*	*D. chrysotoxum*	*D. aphyllum*	*D. aduncum*	*D. aduncum*
	*D. chrysotoxum*	*D. minutiflorum*	*D. falconeri*	*D. brymerianum*	*D. brymerianum*	*D. brymerianum*
	*D. scoriarum*	*D. thyrsiflorum*	*D. flexicaule*	*D. devonianum*	*D. chrysanthum*	*D. chrysanthum*
			*D. gratiosissimum*	*D. fimbriatum*	*D. chrysotoxum*	*D. chrysotoxum*
			*D. heterocarpum*	*D. scoriarum*	*D. crystallinum*	*D. devonianum*
			*D. minutiflorum*		*D. devonianum*	*D. falconeri*
			*D. scoriarum*		*D. falconeri*	*D. gibsonii*
					*D. gibsonii*	*D. harveyanum*
					*D. harveyanum*	*D. heterocarpum*
					*D. heterocarpum*	*D. jenkinsii*
					*D. jenkinsii*	*D. loddigesii*
					*D. loddigesii*	*D. minutiflorum*
					*D. pendulum*	*D. pendulum*
						*D. wardianum*

## Discussion

Ideally, sequences to be used as “reference” for comparison and inferring taxonomic identity or phylogenetic relationship of a query sample should be generated from a voucher specimen or a vouchered material that had been identified by an expert of the taxonomic group. Voucher specimen allows future verification and taxonomic revision, and the presence of a voucher specimen from which the DNA sequence is generated provides scientific credibility and traceability to the sequence and the study. To accurately identify a medicinal material by DNA-based method, one would require not just the reference sequences of the genuine species, but also those of closely-related species and common adulterants. It would be tempting to adopt the sequences already deposited in public database for analysis in DNA-based identification test, but this should not be blindly done presuming taxonomic accuracy of all sequences. The abundance of accessions with compromised annotation in the ever-growing GenBank database would be confusing and further complicate the data analysis for species identification, especially for species with sequences submitted by only one research group. This study found that the availability of information vital for traceability and verification of the GenBank accessions is far from satisfactory. The incomplete and compromised annotations are not much different from the situation revealed by Nilsson et al.^8^. It is quite disappointing to see that the importance of these annotations important for traceability is still overlooked by scientists almost 15 years later. For some accessions, the information is recorded in the publication but not in GenBank. This increase in annotation availability might be because of a more stringent requirement on sample information and its origin by journals or reviewers.

For assessment of taxonomic accuracy or reliability of public sequences, previous studies have adopted two kinds of strategies. One is by generating reliable DNA sequences directly from curated reference materials from museums or herbariums and comparing the sequences deposited in the public database to those reference sequences obtained, as performed by Meiklejohn et al.^12^. Generating more reference sequences is of great importance, but it is difficult to collect voucher specimens of dozens or hundreds of congeneric species in a short period of time. A more commonly adopted approach is by assessing the percentage of sequence similarity, either by searching against a database with BLAST or by comparing the downloaded sequences against each other with clustering^11^. Earlier studies are more lenient and allowed a lower similarity threshold. Bridge et al. considered fungal ITS sequences with over 90% similarity with those of at least three closely related species “identified”^7^. Nilsson et al. regarded fungal ITS sequence with at least 98.5% identity to other matched sequence of the same species in BLAST a thorough match^8^, commenting that it was more stringent than the informal 3% rule species delimitation among bacteria and fungi. The recent study of metazoan mitochondrial sequences by Leray et al. involves clustering of metazoan mitochondrial sequences at 97%, 98%, 99% and 100% thresholds and clusters containing multiple taxonomic groups were further examined to estimate the number of misassigned sequences. They found a very low percentage of mislabelled sequences at genus level (0.67–3.47%) based on non-solitary clusters at the 97% clustering threshold^11^. The selection of cut-off threshold is inevitably arbitrary. But the level of sequence similarity typical of intraspecific variation of the target genes and the organisms studied should be taken into account. In previous studies, sequence similarity is the only assessment criteria for sequence accuracy. Sequences of the same species submitted by only one group might be regarded as acceptable as long as they have high sequence similarity to each other. This would overlook the possibility that all source samples might have been mis-identified by the group, leading to the deposition of multiple mis-identified and mislabelled sequences. In this study, we focus on *Dendrobium*, a genus known to include a lot of genetically closely-related species that cannot be discriminated by sequences on single DNA barcode loci. We have, therefore, set two cut-off thresholds, 99% and 97% similarity, with two additional criteria, (1) whether similar sequences have been submitted by two or more research groups, and (2) whether the declared species is the best-matched species in BLAST.

After an extensive versus-all Megablast search and individual analyses, 7.14% of all evaluated sequences were regarded as highly doubted. This low value is quite encouraging but it should be noted that sequences submitted by only one research group could not be assessed by this approach. It is quite alarming that nine provisional refseq accessions of complete chloroplast genomes were found to contain “highly doubted” barcode sequences. This would be a convincing reminder of the importance of evaluating the taxonomic reliability of sequences in under-curated public databases. When it comes to discriminatory power at species level, our results are similar to those of Xu et al., who found that ITS and ITS2 were the best single loci for species discrimination with 31.93% and 22.29% successful rate in tree-based methods. Another similar finding is the low level of species discrimination of the barcode psbA-trnH, which could only discriminate one (*Dendrobium harveyanum*) in barcoding gap analysis and two species (*Dendrobium thyrisiflorum* and *Dendrobium harveyanum*) in tree-based analysis, respectively. In Xu’s study, psbA-trnH could only discriminate species at 8.14% in tree-based method, and it did not show a distinct barcoding gap in distance analysis. Considering its low discrimination ability and low successful rate in sequencing^24,25^, psbA-trnH intergenic spacer is not a good barcode candidate for *Dendrobium* species, despite being recommended as a complementary barcode to ITS2 for medicinal plants^26^. We did not perform multi-locus analysis as not all of the 41 species have sequences of all seven barcode candidates available. And it would be out of the original scope of this study, which is to evaluate the annotation comprehensiveness and taxonomic reliability of individual barcode sequences of the 41 *Dendrobium* species in GenBank. For the identification of *Dendrobium* species, an ITS + matK combination, which was found to have the highest discriminatory power at 76.92% by Xu et al.^25^, could be considered. It would be better if one would ensure the discriminatory power of the potential barcode combinations on the suspected/target species, before deciding the loci or combination to use. A simple workflow has been included to show how to download sequences with optional filtering to select accessions annotated with voucher specimen number, country, or collector from GenBank (Supplementary File 1). After downloading the sequences, alignment and phylogenetic analysis could be performed to estimate the ability of the barcode(s) to discriminate the concerned species. Species-specific PCR identification methods have already been developed for *D. officinale*^27^ and *D. huoshanense*^28^ as an Association Standard or in Chinese Pharmacopoeia (2020 Edition), respectively, to allow quick and accurate identification at a lower operating cost.

It would be impossible to know the true reasons causing the incorrect species assignment, as well as to confirm the erroneous taxonomic identity of the sequences, without re-amplifying and re-sequencing from the original sample. This is another example showing the importance of keeping a voucher specimen. Likely sources of error previously reported include sample misidentification, laboratory contaminations, data entry error, contamination by associated organisms in the sample and pseudogenes^11,13^. The mis-assignment to congeneric species could be resulted from misidentification of samples, or cross-sample contamination when samples of multiple *Dendrobium* species were handled, extracted and amplified at the same time. We have come across two different psbA-trnH sequences originating from the same voucher specimen (EF590688 and GQ248286). In this case, either one of the reporting groups have made some mistakes in the experimental work or in data entry, or the voucher specimen contained more than one individual/organism. As previously suggested, some, if not most, of these incorrect assignments should have been detected by a simple BLAST search before sequence submission^11,13,29^. This small step is, unfortunately, also overlooked.

To avoid erroneous sequences in public database, it would be advisable to opt for better curated database like The Barcode of Life Data System (BOLD)^30^. However, the plant identification engine of BOLD only allows BLAST search of rbcL or matK sequences and may not be very useful in species-level identification. Another good move is to download only sequences tagged with a voucher specimen from GenBank. This could be easily done by adding words like “AND voucher” in the search box of NCBI Nucleotide page as shown in the workflow (Supplementary File 1). For medicinal herbs, our group has generated the Medicinal Materials DNA Barcode Database collecting barcode sequences of genuine medicinal herbs and those of their common adulterants in a simple one-stop platform for sequence alignment and primer design^31^. In situations when correct species-level identification is necessary, one must confirm the availability of reliable sequences of the target and closely related species, as well as the species discrimination ability of the sequences or the validity of the method employed, before carrying out any experimental work.

## Supplementary Information

Supplementary Information 1.

Supplementary Information 2.

Supplementary Figure S1.

Supplementary Figure S2.

Supplementary Figure S3.

Supplementary Figure S4.

Supplementary Figure S5.

Supplementary Figure S6.

Supplementary Figure S7.
