# Supplementary material for: Assessing the reliability of medicinal Dendrobium sequences in GenBank for botanical species identification
Source: Sci Rep. 2021 Feb 9;11:3439. doi: 10.1038/s41598-021-82385-z (PMC7873228; doi:10.1038/s41598-021-82385-z)

Assessing the reliability of medicinal *Dendrobium* sequences in GenBank for botanical species identification

Hoi-Yan WU[1], Kwun-Tin CHAN[1,3], Grace Wing-Chiu BUT[3], Pang-Chui SHAW[1,2,3*]

[1] Li Dak Sum Yip Yio Chin R & D Centre for Chinese Medicine, The Chinese University of Hong Kong, Hong Kong

[2] State Key Laboratory of Research on Bioactivities and Clinical Applications of Medicinal Plants (The Chinese University of Hong Kong) and Institute of Chinese Medicine, The Chinese University of Hong Kong, Hong Kong

[3] School of Life Sciences, The Chinese University of Hong Kong, Hong Kong

- Corresponding authors: Pang-Chui Shaw; School of Life Sciences, The Chinese University of Hong Kong, Hong Kong; Tel: +852-3943-1363; Fax: +852-2603-7246; Email: pcshaw@cuhk.edu.hk

A simple workflow to download nucleotide sequences of a particular taxonomic group from GenBank with optional filtering for essential annotation.

GenBank is a huge and ever-growing public genetic sequence database, collecting all publicly available sequences with daily data exchange with the DNA DataBank of Japan and the European Nucleotide Archive. It is accessible to all, providing a user-friendly web-based interface and some important programs like BLAST that allows searching and alignment of GenBank sequences. However, GenBank accepts sequences from any kind of materials/specimens by almost anyone, with or without expert taxonomic authentication of sample. In this study, we have shown the inadequacy of annotation of sample source in the accessions of DNA barcodes of *Dendrobium* species. Here, we present a simple workflow showing how to download nucleotide sequences of a particular taxonomic group from the GenBank web interface with filtering to filter out insufficiently annotated sequences.

Introduction to source annotation in GenBank

GenBank collects information of a list of source modifiers when user submits the sequences. The provision of such information is optional. Here are some source identifiers reflecting reliability or traceability, at least to a certain degree, of the source sample.

https://www.ncbi.nlm.nih.gov/WebSub/html/help/genbank-source-table.html#modifiers

Table S2. The description and use of the source modifiers

Source modifier	Description	Use
Specimen_voucher	A preserved and representative sample of the individual or collection of the source organism that is used for verification and identification.	Showing physical traceability of source material
Bio_material	A preserved and representative biological material from which the DNA information was acquired, and organised with suitable label for the location of storage.	
Country	The nation where the source material was found.	Showing the country of origin or locality of the source material
Collected_by	The person or persons who collected the source material.	Showing traceability of taxonomic identity entered into GenBank
Identified_by	The person or persons who identified the source material.	

How to access genetic sequences of a particular taxonomic group

1. Go to https://www.ncbi.nlm.nih.gov/taxonomy. Enter the name of the taxonomic group, either a species name, genus name, or the name of other taxonomic ranks. Genus *Dendrobium* is used for demonstration. Press the Enter key or click search to proceed.

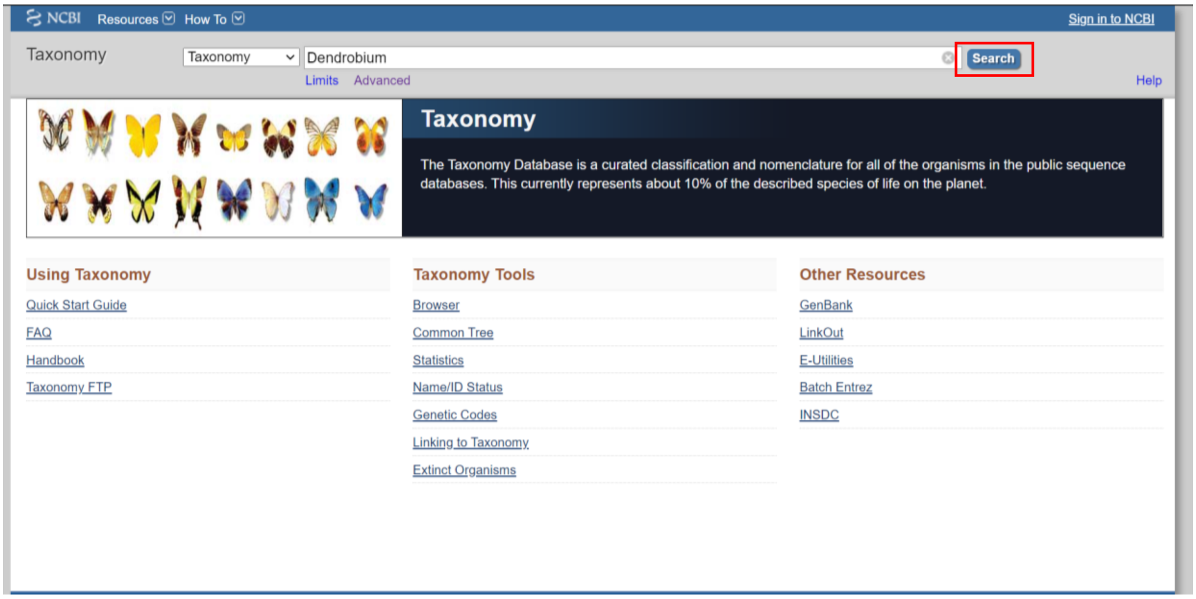

2. Click "Nucleotide" under the search result to search for the nucleotide sequence and relative information.

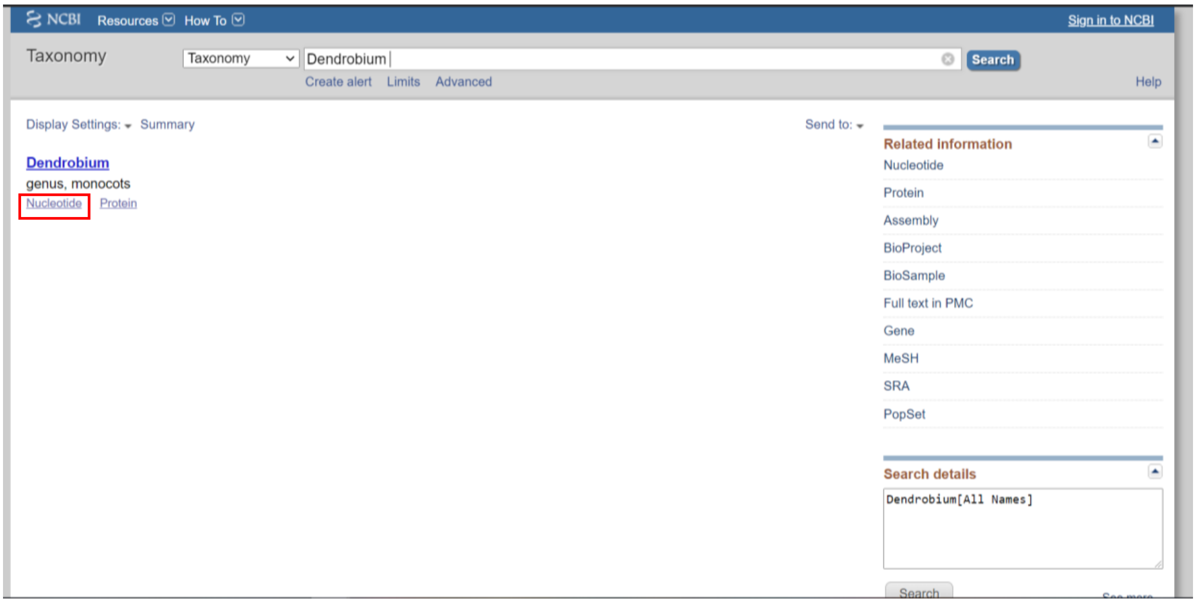

3. The taxonomic ID of *Dendrobium* (txid37818) is shown at this step. Click "Advanced" to refine the search.

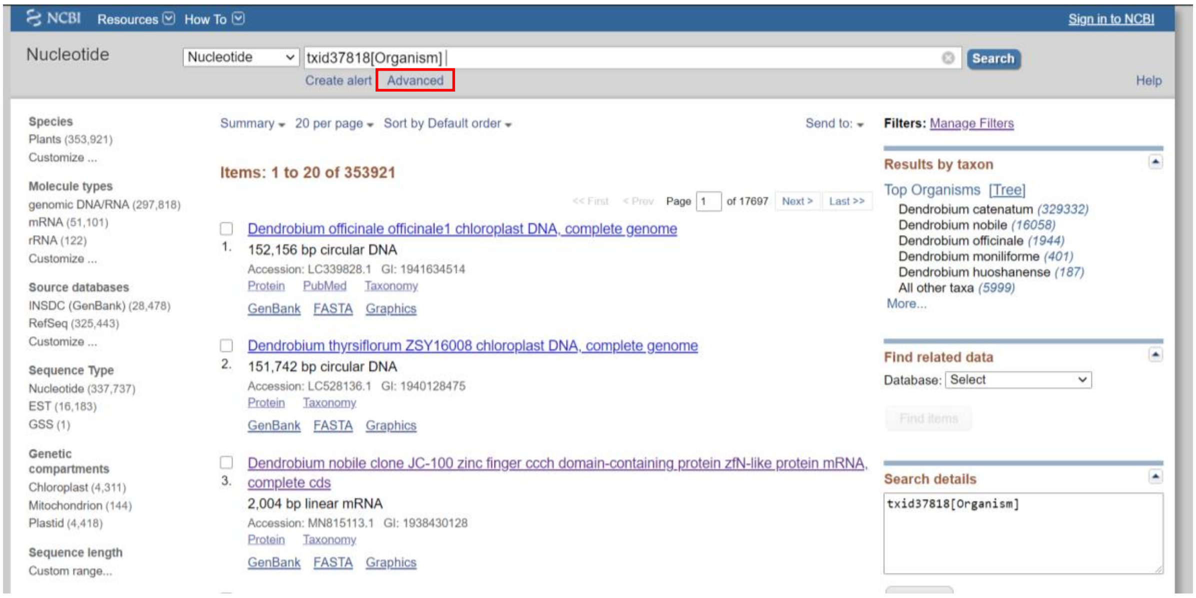

4. In the "Nucleotide Advanced Search Builder" section, different terms can be added to the query box to narrow down the search. For instance, we added several keywords including DNA barcode, voucher, country and collected for a more flexible, convenient search.

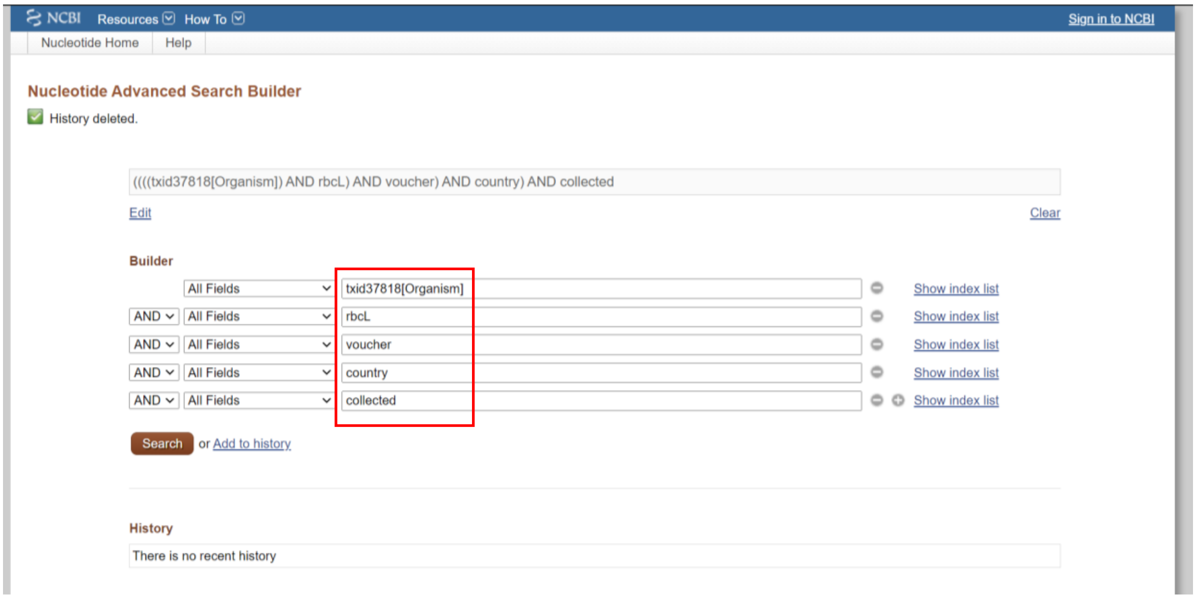

4. The search result was refined into 48 items from 353921 items.

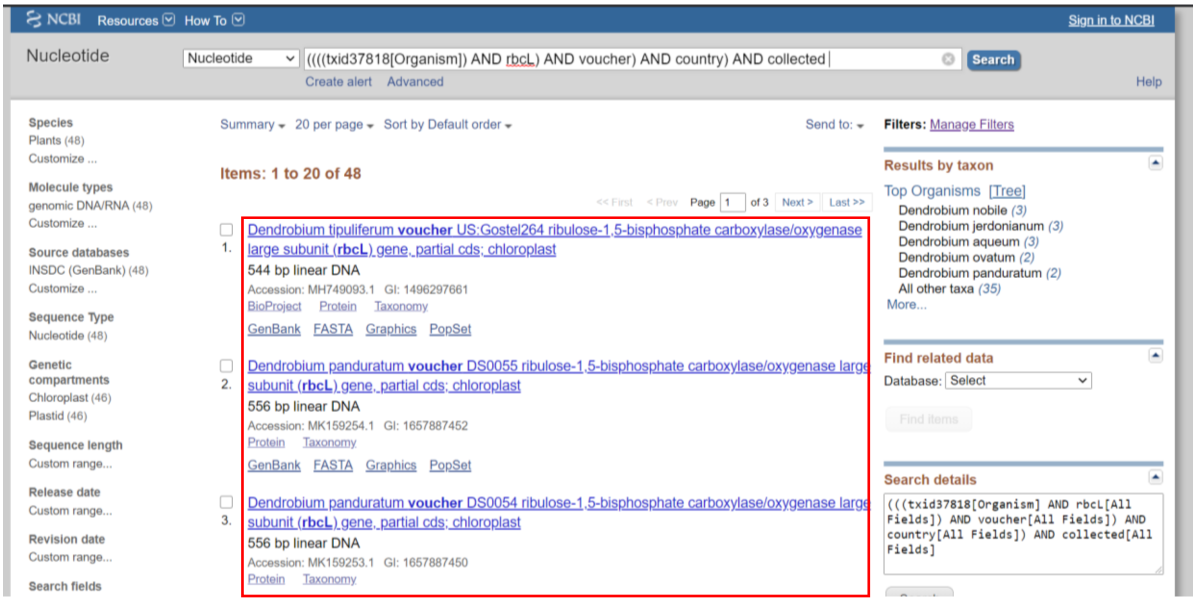

5. To filter for “identified by” instead of “collected by”, we replaced “collected” into “identified”.

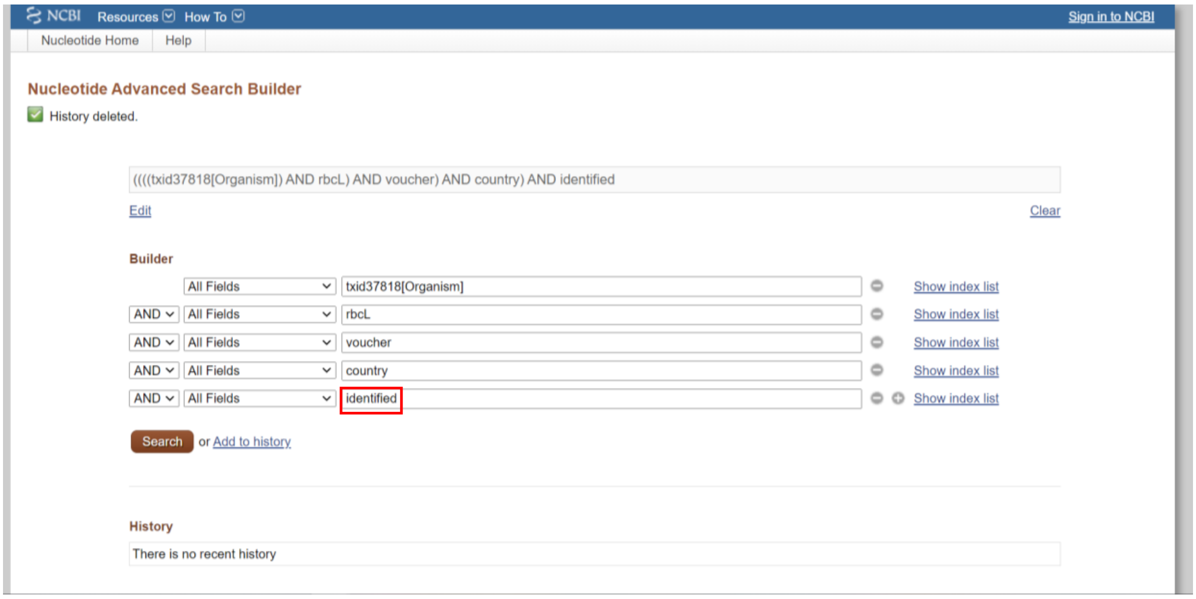

6. Only 5 search results can be found. Sometimes, you may get some accessions that appear to belong to another taxonomic group. It is due to change of taxonomic name or grouping done by NCBI after submission of accession by the user. The search results show the “DEFINITION” of the record, which remains unchanged, even when the ORGANISM and Taxonomy ID have been changed.

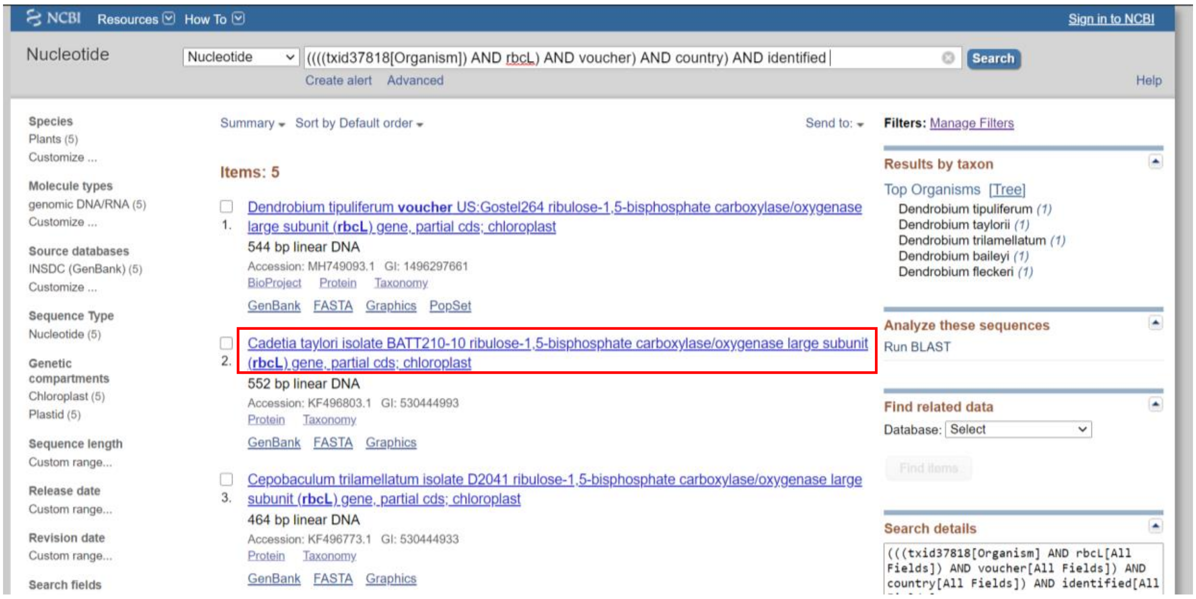

7. The second result was shown as *Cadetia taylori*. However, the organism of the source has been updated to *Dendrobium taylorii*.

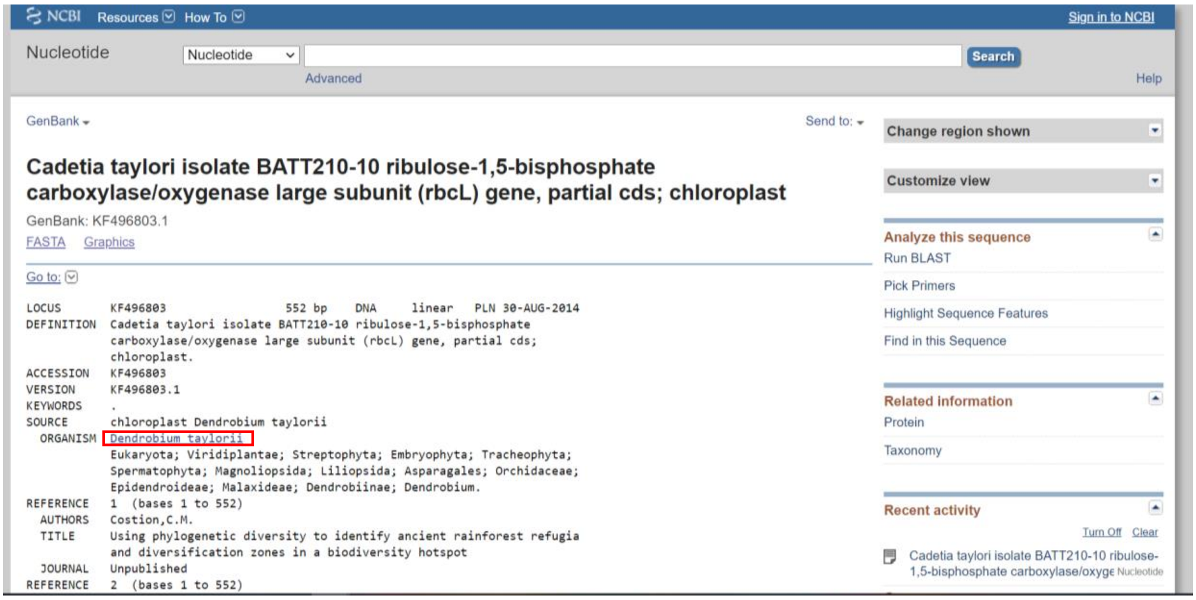

8. To download sequences and relative information, click "Send to", followed by "File" and choose the "Format" to download.

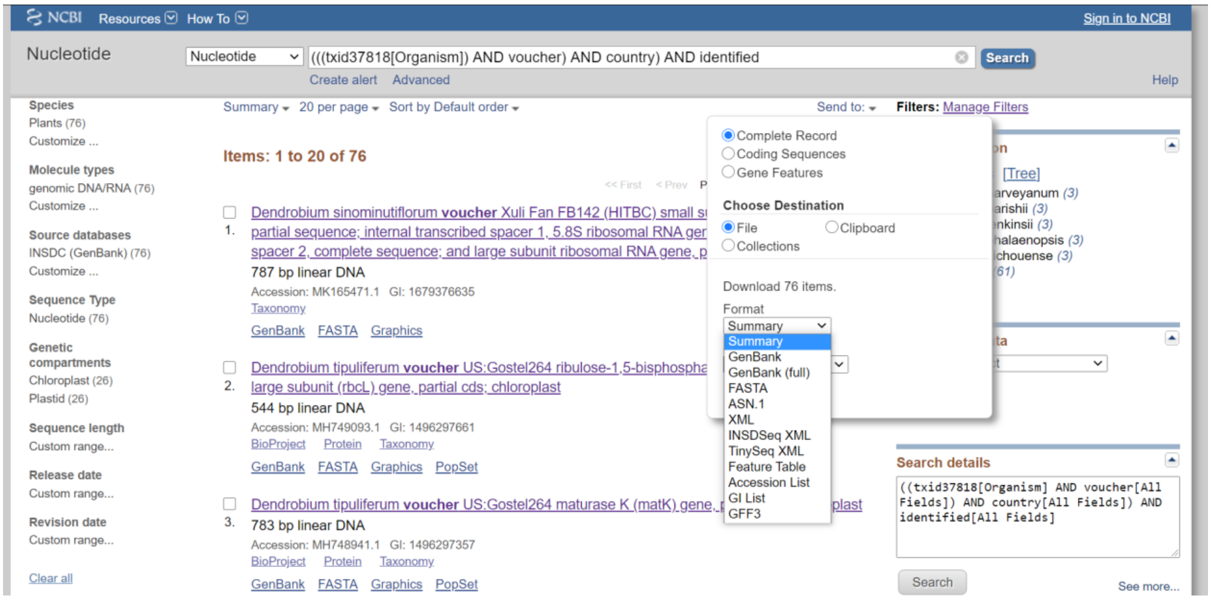

Supplement: Supplementary file 1 — Supplementary Information 1. [file 41598_2021_82385_MOESM1_ESM.pdf]
