# Supplementary material for: Assessing the reliability of medicinal Dendrobium sequences in GenBank for botanical species identification
Source: Sci Rep. 2021 Feb 9;11:3439. doi: 10.1038/s41598-021-82385-z (PMC7873228; doi:10.1038/s41598-021-82385-z)
Supplement: Supplementary file 2 — Supplementary Information 2. [file 41598_2021_82385_MOESM2_ESM.docx]

**Assessing the reliability of medicinal *Dendrobium* sequences in GenBank for botanical species identification**

Hoi-Yan WU^1^, Kwun-Tin CHAN^1,3^, Grace Wing-Chiu BUT^3^, Pang-Chui SHAW^1,2,3*^

^1^ Li Dak Sum Yip Yio Chin R & D Centre for Chinese Medicine, The Chinese University of Hong Kong, Hong Kong

^2^ State Key Laboratory of Research on Bioactivities and Clinical Applications of Medicinal Plants (The Chinese University of Hong Kong) and Institute of Chinese Medicine, The Chinese University of Hong Kong, Hong Kong

^3^ School of Life Sciences, The Chinese University of Hong Kong, Hong Kong

- Corresponding authors: Pang-Chui Shaw; School of Life Sciences, The Chinese University of Hong Kong, Hong Kong; Tel: +852-3943-1363; Fax: +852-2603-7246; Email: [pcshaw@cuhk.edu.hk](mailto:pcshaw@cuhk.edu.hk)

**Supplementary Material**

**Table S1 | List of highly doubted sequences with reason of doubt.**

**Figure S1. Neighbor-Joining (NJ) tree based on rbcL sequences of *Dendrobium* from GenBank.**

The condensed tree (50% bootstrap consensus tree) showing only

clustering topology is presented. Evolutionary distances of 624 sequences were inferred using Kimura two-parameter model. Sequences of *Liparis kumokiri* were used as outgroup to root the tree. GenBank accessions are displayed with corresponding species name.

**Figure S2. Neighbor-Joining (NJ) tree based on matK sequences of *Dendrobium* from GenBank.**

The condensed tree (50% bootstrap consensus tree) showing only

clustering topology is presented. Evolutionary distances of 1044 sequences were inferred using Kimura two-parameter model. Sequences of *Liparis kumokiri* were used as outgroup to root the tree. GenBank accessions are displayed with corresponding species name.

**Figure S3. Neighbor-Joining (NJ) tree based on psbA-trnH sequences of *Dendrobium* from GenBank.**

The condensed tree (50% bootstrap consensus tree) showing only

clustering topology is presented. Evolutionary distances of 529 sequences were inferred using Kimura two-parameter model. Sequences of *Liparis kumokiri* were used as outgroup to root the tree. GenBank accessions are displayed with corresponding species name.

**Figure S4. Neighbor-Joining (NJ) tree based on trnL-trnF sequences of *Dendrobium* from GenBank.**

The condensed tree (50% bootstrap consensus tree) showing only

clustering topology is presented. Evolutionary distances of 107 sequences were inferred using Kimura two-parameter model. Sequences of *Liparis kumokiri* were used as outgroup to root the tree. GenBank accessions are displayed with corresponding species name.

**Figure S5. Neighbor-Joining (NJ) tree based on trnL sequences of *Dendrobium* from GenBank.**

The condensed tree (50% bootstrap consensus tree) showing only

clustering topology is presented. Evolutionary distances of 227 sequences were inferred using Kimura two-parameter model. Sequences of *Liparis kumokiri* were used as outgroup to root the tree. GenBank accessions are displayed with corresponding species name.

**Figure S6. Neighbor-Joining (NJ) tree based on ITS1 sequences of *Dendrobium* from GenBank.**

The condensed tree (50% bootstrap consensus tree) showing only

clustering topology is presented. Evolutionary distances of 1645 sequences were inferred using Kimura two-parameter model. Sequences of *Liparis kumokiri* were used as outgroup to root the tree. GenBank accessions are displayed with corresponding species name.

**Figure S7. Neighbor-Joining (NJ) tree based on ITS2 sequences of *Dendrobium* from GenBank.**

The condensed tree (50% bootstrap consensus tree) showing only

clustering topology is presented. Evolutionary distances of 1794 sequences were inferred using Kimura two-parameter model. Sequences of *Liparis kumokiri* were used as outgroup to root the tree. GenBank accessions are displayed with corresponding species name.

**Table S1. List of highly doubted sequences with reason of doubt**

| **Barcode** | **Accession** | **Species** | **Reason of doubt** |
| --- | --- | --- | --- |
| ITS1 | HM590372 | *D. aduncum* | Only 97.40% or less similar to other *D. aduncum* sequences. Best match to AB593581 *D. hercoglossum* at 98.27% similarity. |
| ITS1 | HQ114249 | *D. aduncum* | Not match to any other *D. aduncum* sequences. 100% match to AF362043 *D. aurantiacum* var. *denneanum*. |
| ITS1 | HQ114250 | *D. aduncum* | Not match to any other *D. aduncum* sequences. 100% match to KY966562 *D. loddigesii.* |
| ITS2 | HM590372 | *D. aduncum* | Only 97.52% or less similar to other *D. aduncum* sequences. 100% match to various sequences of *D. hercoglossum, D. nobile* and *D. officinale*. |
| ITS2 | HQ114249 | *D. aduncum* | Not match to any other *D. aduncum* sequences. 99.59% match to *D. chryseum*, *D. aurantiacum* var. *denneanum* and *D. jiajiangense*. |
| ITS2 | HQ114250 | *D. aduncum* | Not match to any other *D. aduncum* sequences. 99.58% match to KY966562 *D. loddigesii*. |
| ITS2 | KY745835 | *D. aduncum* | Only 97.56% or less similar to other *D. aduncum* sequences. 100% similar to various sequences of *D. hercoglossum*, *D. linawianum*, *D. nobile* and *D. officinale*. |
| psbA-trnH | FJ216483 | *D. aduncum* | Only 98.02% or less similar to other *D. aduncum* sequences. Best match (100%) to a lot of *D. aurantiacum* var. *denneanum* sequences. |
| psbA-trnH | KJ174241 | *D. aduncum* | Only 98.15% or less similar to other *D. aduncum* sequences. Best match to EU887928 *D. harveyanum* at 99.87% similarity. |
| psbA-trnH | KP159307 | *D. aduncum* | Only 97.55% or less similar to other *D. aduncum* sequences. Best match to KP159309 *D. hercoglossum* at 100% similarity. |
| trnL | KC568307 | *D. aduncum* | Not match to any other *D. aduncum* sequences. 99.86% match to KF143577 *D. hercoglossum*. |
| ITS1 | KC205201 | *D. aphyllum* | Not match to any other *D. aduncum* sequences. Best match to JN388570 *D. anosmum* at 99.57%. |
| ITS2 | KC205201 | *D. aphyllum* | Not match to any other *D. aduncum* sequences. Best match to MT004845 *D. anosmum* at 100% similarity. |
| matK | KF361660 | *D. aphyllum* | Not match to any *Dendrobium* sequences. Best match to KF361659 *Oberonia ensiformis* at 99.88%. |
| chloroplast trnL | LC192955 | *D. aurantiacum var. denneanum* | Not match to any *D. deanneanum* sequences. Best match to LC348863 *D. shixingense* at 100% similarity. |
| chloroplast trnL-trnF | LC192955 | *D. aurantiacum var. denneanum* | Not match to any *D. deanneanum* sequences. Best match to LC348863 *D. shixingense* at 100% similarity. |
| trnL | EF397918 | *D. aurantiacum var. denneanum* | Best match to LC192810. *D. primulinum* at 91.65%. |
| trnL | KF143555 | *D. aurantiacum var. denneanum* | Only 95.36% similar to other *D. deannean* sequences. Best match to KF143567 *D. fimbriatum* at 97.67%. |
| trnL | KF143556 | *D. aurantiacum var. denneanum* | Only 96.93% similar to other *D. deannean* sequences. Best match to *D. aurantiacum* KP749353 at 100% similarity and LC193521 *D. fimbriatum* at 99% similarity. |
| trnL-trnF | EF397918 | *D. aurantiacum var. denneanum* | Best match to LC192810 *D. primulinum* at 91.65%. |
| ITS1 | FJ428221 | *D. brymerianum* | Only 89.18% or less similar to other *D. brymerianum* sequences. Best match to KJ210416.1 *D. aurantiacum* at 100% similarity. |
| ITS1 | AY485693 | *D. brymerianum* | Not match to other *D. brymerianum* sequences. Best match to AY485692 *D. officinale* at 96.56%, followed by MT295882 *D. officinale* at 92.44%. |
| ITS2 | FJ428221 | *D. brymerianum* | Not match to other *D. brymerianum* sequences. Best match to MT004870 *D. fimbriatum* and KJ210416 *D. aurantiacum* at 100%. |
| ITS2 | AY485693 | *D. brymerianum* | Not match to other *D. brymerianum* sequences. Best match to AY485692 *D. officinale* at 94.63%, followed by AF311776 *D. officinale* at 92.98%. |
| psbA-trnH | KP412232 | *D. brymerianum* | Only 94.56% or less similar to other *D. brymerianum* sequences. Best match to GU458304 *D. nobile* at 100% similarity. |
| ITS1 | HM590379 | *D. capillipes* | Not match to any other *D. capillipes* sequences. 100% match to HM590383 *D. chrysotoxum*. |
| ITS2 | HM590379 | *D. capillipes* | Not match to any other *D. capillipes* sequences. 100% match to MT004851 *D. chrysotoxum*. |
| matK | AB972322 | *D. capillipes* | Best match to *D. heterocarpum* AB847779 at 99.91%. |
| trnL | EF397912 | *D. capillipes* | Not match to any other *D. capillipes* sequences. Best match to *D. chrysotoxum* at 99.92% |
| trnL | KF143539 | *D. capillipes* | Only 95.61% similar to EF397912 *D. capillipes*. Best match to EF397915 *D. chrysotosum* at 95.73%. |
| ITS1 | AY485713 | *D. chrysanthum* | Only 94.30% or less similar to other *D. chrysanthum* sequences. |
| ITS1 | KC205191 | *D. chrysanthum* | Not match to any other *D. chrysanthum* sequences. Best match to KC205203 *D. scoriarum* and JN388569 *D. loddigesii*. |
| ITS1 | KY966522 | *D. chrysanthum* | Not match to any other *D. chrysanthum* sequences. Best mathc to KT778763 *D. fimbriatum*. |
| ITS2 | AY485713 | *D. chrysanthum* | Only 94.04% or less similar to other *D. chrysanthum* sequences. |
| ITS2 | KC205191 | *D. chrysanthum* | Not match to any other *D. chrysanthum* sequences. Best match to KC205203 *D. scoriarum* and several *D. loddigesii* sequences at 100%. |
| ITS2 | KY966522 | *D. chrysanthum* | Not match to any other *D. chrysanthum* sequences. Best match to several *D. fimbriatum* sequences at 100%. |
| trnL | KF143549 | *D. chrysanthum* | Only 97.42% similar to the other *D. chrysanthum* sequence. Best match to KC568309 *D. strongylanthum* at 98.05%. |
| trnL | LC193514 | *D. chrysanthum* | Not match to other *D. chrysanthum* sequence. Best match to LC193516 *D. lohohense* at 98.07%. |
| chloroplast matK | KT633996 | *D. chrysotoxum* | Not match to any *Dendrobium* sequences. 100% match to HQ130494 *Otochilus lancilabius*. |
| chloroplast psbA-trnH | KT633996 | *D. chrysotoxum* | Not match to any *Dendrobium* sequences. Best match to KC704331 *Bletilla striata* at 95.50%. |
| chloroplast rbcL | KT633996 | *D. chrysotoxum* | Not match to any *Dendrobium* sequences. Best match to MT193723 *Bletilla striata* at 99.39%. |
| chloroplast trnL-trnF | KT633996 | *D. chrysotoxum* | Not match to any *Dendrobium* sequences. Best match to MK356240 *Coelogyne prasina* at 99.75% |
| ITS1 | KC205204 | *D. chrysotoxum* | Only 84.98% or less similar to other *D. chrysotoxum* sequences |
| ITS2 | KC205204 | *D. chrysotoxum* | Only 79.17% similar to its best match *D. chrysotoxum* sequence. |
| trnL-trnF | EF3979115 | *D. chrysotoxum* | Only 97.93% similar to the other *D. chrysotoxum* sequence. Best match to EF397912 *D. capillipes*  at 99.92% . |
| chloroplast psbA-trnH | LC193509 | *D. crepidatum* | Only 98.76% match to the other *D. crepidatum* sequence. Best match to LC192961 *D. wardianum* at 99.38%. |
| chloroplast trnL | LC193509 | *D. crepidatum* | Only 96.77% or less similar to other *D. crepidatum* sequences. Best match to LC193517 *D. chrysotoxum* at 97.74% |
| ITS1 | HQ114241 | *D. crepidatum* | 100% similar to KY499227 *D. crepidatum*. But both sequences are only 82.98% or less similar to other *D. crepidatum* sequences. Best match to KY928056 *D. chrysanthum* at 100%. |
| ITS1 | KY499227 | *D. crepidatum* | 100% similar to HQ114241 *D. crepidatum*. But both sequences are only 82.98% or less similar to other *D. crepidatum* sequences. Best match to KY928056 *D. chrysanthum* at 100%. |
| ITS2 | HQ114241 | *D. crepidatum* | 100% similar to KY499227 *D. crepidatum*. But both sequences are only 82.98% or less similar to other *D. crepidatum* sequences. Best match to KY928056 *D. chrysanthum* at 100%. |
| ITS2 | KY499227 | *D. crepidatum* | 100% similar to HQ114241 *D. crepidatum*. But both sequences are only 82.98% or less similar to other *D. crepidatum* sequences. Best match to KY928056 *D. chrysanthum* at 100%. |
| ITS2 | KY508515 | *D. crepidatum* | Only 93.88% or less similar to other *D. crepidatum* sequences. |
| ITS1 | AF363023 | *D. crystallinum* | 99.53% similar to KJ672662 *D. crystallinum*, but only 91.85% or less similar to all other *D. crystallinum* sequences. Second best match is MT295894 *D. moniliforme* at 92.27%. |
| ITS1 | KJ672662 | *D. crystallinum* | 99.53% similar to AF363023 *D. crystallinum*, but only 91.85% or less similar to all other *D. crystallinum* sequences. Second best match is KJ672630 *D. moniliforme* at 92.49%. |
| ITS1 | FJ384742 | *D. densiflorum* | Not match to other *D. densiflorum* sequences. Best match to JN388594 *D. harveyanum* at 100%. |
| ITS1 | KY966533 | *D. densiflorum* | Only 93.72% or less similar to other *D. densiflorum* sequences. Best match to AB593574 *D. guibertii* at 100%. |
| ITS2 | FJ384742 | *D. densiflorum* | Not match to other *D. densiflorum* sequences. Best match to KJ210452 *D. harveyanum* at 100%. |
| ITS2 | KY966533 | *D. densiflorum* | Only 97.57% or less similar to other *D. densiflorum* sequences. Best match to AB593574 *D. guibertii* at 100%. |
| psbA-trnH | KP412228 | *D. densiflorum* | Only 97.46% or less similar to other *D. densiflorum* sequences. Best match to KP412231 *D. crepidatum* and KJ174248 *D. chrysanthum* at 100% similarity. |
| psbA-trnH | KP412229 | *D. densiflorum* | Not match to other *D. densiflorum* sequences. Best match to KP412231 *D. crepidatum* and KJ174248 *D. chrysanthum* at 100% similarity. |
| trnL | KF143557 | *D. densiflorum* | Only 90.71% or less similar to other *D. densiflorum* sequences. Best match to AF519939 *Bletilla striata* at 100% similarity. |
| ITS1 | EF629322 | *D. devonianum* | Not match to other D. denvonianum sequences. Best match to KX600513 *D. heterocarpum* at 93.04% similarity |
| ITS2 | EF629322 | *D. devonianum* | Not match to any other sequences except for itself. |
| ITS1 | AY485708 | *D. falconeri* | Only 93.83% or less similar to other *D. falconeri* sequences. |
| ITS2 | AY485708 | *D. falconeri* | Only 93.22% or less similar to other *D. falconeri* sequences. |
| psbA-trnH | KJ174259 | *D. falconeri* | Not match to other *D. falconeri* sequences. Best match to EU672797 *D. nobile* at 99.48% similarity. |
| rbcL | DQ449478 | *D. falconeri* | Not match to other *D. falconeri* sequences. Best match to KY635881 *Litchi chinensis* and DQ449472 *D. crumenatum* at 100% similarity. |
| trnL | EF397920 | *D. falconeri* | Not match to other *D. falconeri* sequences. Best match to LC192961 *D. wardianum* at 91.69%. |
| ITS1 | KX522634 | *D. fimbriatum* | Only 87.93% or less similar to other *D. fimbriatum* sequences. Best match to KT778750 *D. moschatum*. |
| ITS1 | AF362041 | *D. fimbriatum* | Only 84.55% or less similar to other *D. fimbriatum* sequences. Best match to AB593568 *D. gibsonii* at 100% similarity. |
| ITS2 | AY842036 | *D. fimbriatum* | Not match to other *D. fimbriatum* sequences. Best match to AY485713 *D. chrysanthum* at 93.86% similarity |
| ITS2 | KX522634 | *D. fimbriatum* | Not match to other *D. fimbriatum* sequences. Best match to MN263240 *D. moschatum* at 100% similarity. |
| ITS2 | AF362041 | *D. fimbriatum* | Not match to other *D. fimbriatum* sequences. Best match to MK522204 *D. gibsonii* at 99.18% similarity. |
| ITS2 | AY485714 | *D. fimbriatum* | Only 91.43% or less similar to other *D. fimbriatum* sequences. |
| ITS1 | HQ114256 | *D. gibsonii* | Only 85.84% or less similar to other *D. gibsonii* sequences. Best match to KJ210418 *D. aurantiacum* at 100% similarity. |
| ITS2 | HQ114256 | *D. gibsonii* | Only 97.13% or less similar to other *D. gibsonii* sequences. Best match to KY745857 *D. aurantiacum* at 100% similarity. |
| chloroplast psbA-trnH | LC192958 | *D. gratiosissimum* | Not match to any other *D. gratiosissimum* sequences. Best match to KP704440 *D. catenatum* at 99.19% similarity |
| chloroplast trnL | LC192958 | *D. gratiosissimum* | Not match to any other *D. gratiosissimum* sequences. Best match to LC192961 *D. wardianum* at 94.73% similarity |
| ITS1 | AY485711 | *D. gratiosissimum* | Only 95.54% or less similar to other *D. gratiosissimum* sequences. |
| ITS1 | HQ114236 | *D. gratiosissimum* | 100% similar to KR075043 *D. gratiosissimum*. But both are only 88.11% or less similar to other *D. gratiosissimum* sequences. Best match to KJ210410 *D. aduncum* at 100% similarity. |
| ITS1 | KR075043 | *D. gratiosissimum* | 100% similar to HQ114236 *D. gratiosissimum*. But both are only 88.11% or less similar to other *D. gratiosissimum* sequences. Best match to KJ210410 *D. aduncum* at 100% similarity. |
| ITS1 | KX522646 | *D. gratiosissimum* | Only 89.47% or less similar to other *D. gratiosissimum* sequences. Best match to KY966529 *D. crystallinum* at 99.57% similarity. |
| ITS1 | KY499213 | *D. gratiosissimum* | Only 94.74% or less similar to other *D. gratiosissimum* sequences. Best match to MK483278 *D. transparens* at 100% similarity. |
| ITS2 | AY485711 | *D. gratiosissimum* | Only 94.98% or less similar to other *D. gratiosissimum* sequences. |
| ITS2 | HQ114236 | *D. gratiosissimum* | 100% similar to KR075043 *D. gratiosissimum*. But both are only 94.98% or less similar to other *D. gratiosissimum* sequences. Best match to MK522248 *D. aduncum* at 100% similarity. |
| ITS2 | KR075043 | *D. gratiosissimum* | 100% similar to HQ114236 *D. gratiosissimum*. But both are only 94.98% or less similar to other *D. gratiosissimum* sequences. Best match to MK522248 *D. aduncum* at 100% similarity. |
| ITS2 | KX522646 | *D. gratiosissimum* | Only 93.47% or less similar to other *D. gratiosissimum* sequences. Best match to KY966529 *D. crystallinum* at 99.57% similarity. |
| ITS2 | KY499213 | *D. gratiosissimum* | Only 91.87% or less similar to other *D. gratiosissimum* sequences. Best match to MK483278 *D. transparens* at 100% similarity. |
| matK | KR075050 | *D. gratiosissimum* | Not match to any other *D. gratiosissimum* sequences. Best match to AB847680 *D. aduncum* at 99.79% |
| psbA-trnH | FJ216472 | *D. gratiosissimum* | Only 96.01% or less similar to other *D. gratiosissimum* sequences. Best match to EU887932 *D. brymerianum* at 97.26% similarity. |
| rbcL | KR075078 | *D. gratiosissimum* | Not match to any other *D. gratiosissimum* sequences. Best match to KP704495 *D. strongylanthum* at 100% similarity |
| psbA-trnH | KP159306 | *D. hancockii* | Not match to any *Dendrobium* sequences. Best match to HE966664 *Sedum maximum* subsp. maximum at 95.18% similarity. |
| psbA-trnH | KF177507 | *D. harveyanum* | Only 96.44% or less similar to the other *D. harveyanum* sequences. Best match to KF177553.1 *D. strongylanthum* at 99.00% |
| ITS1 | AY485694 | *D. hercoglossum* | Only 92.04% or less similar to other *D. hercoglossum* sequences. Best match to AY485719 *D. nobile* at 94.69%. |
| ITS2 | AY485694 | *D. hercoglossum* | Only 94.65% or less similar to other *D. hercoglossum* sequences. Best match to AY485692 *D. officinale* at 95.87%. |
| ITS2 | KY745858 | *D. hercoglossum* | 100% match to MT004911 *D. hercoglossum*. But both sequences are only 93.90% or less similar to other *D. hercoglossum* sequences. Also best match to MK522248 *D. aduncum* at 100% similarity. |
| psbA-trnH | KJ174270 | *D. hercoglossum* | Only 97.21% or less similar to other *D. hercoglossum* sequences. Best match to KF177512 *D. heterocarpum* at 100% similarity. |
| trnL | KF143577 | *D. hercoglossum* | Not match to other *D. hercoglossum* sequences. Only matched to three *Dendrobium* sequences (KC568307, LC192958 and LC193510) at 100%, 91.50% and 89.96% similarity. The former two sequences are also highly doubted sequences. |
| ITS1 | HQ114235 | *D. heterocarpum* | Not match to other *D. heterocarpum* sequences. Best match to MN221396 *D. moniliforme*, KY966557 *D. linawianum*, KP159300 *D. hercoglossum* and several *D. nobile* sequences at 100% similarity. |
| ITS1 | KY966550 | *D. heterocarpum* | Only 97.44% or less similar to other *D. heterocarpum* sequences. Best match to AB593647 *D. rhombeum* at 97.86% similarity. |
| ITS1 | AY485717 | *D. heterocarpum* | Not match to other *D. heterocarpum* sequences. Best match to AY485692 *D. officinale* at 86.52% similarity. |
| ITS2 | HQ114235 | *D. heterocarpum* | Not match to other *D. heterocarpum* sequences. Best match to multiple sequences of *D. moniliforme*, *D. linawianum*, *D. hercoglossum*, *D. nobile* and *D. aduncum* at 100% similarity. |
| ITS2 | KY966550 | *D. heterocarpum* | Only 97.93% or less similar to other *D. heterocarpum* sequences. Best match to AB972350 *D. capilliepes* at 98.35% similarity. |
| ITS2 | AY485717 | *D. heterocarpum* | Not match to other *D. heterocarpum* sequences. Best match to AY485692 *D. officinale* at 86.89% similarity. |
| psbA-trnH | KF177512 | *D. heterocarpum* | Only 96.96% or less similar to other *D. heterocarpum* sequences. Best match to KJ174283 *D. nobile* at 97.14% similarity. |
| psbA-trnH | KP412185 | *D. heterocarpum* | Only 96.92% or less similar to other *D. heterocarpum* sequences. Best match to EU887941 *D. nobile* at 100% similarity. |
| psbA-trnH | KJ174271 | *D. heterocarpum* | Not match to other *D. heterocarpum* sequences. Best match to KT630834 *D. huoshanense* and several *D. moniliforme* sequences at 100% similarity. |
| ITS1 | KC568300 | *D. huoshanense* | Only 94.49% or less similar to other *D. huoshanense* sequences. Best match to MT295895 *D. moniliforme* at 94.92% similarity. |
| ITS1 | HM590368 | *D. huoshanense* | Only 83.13% or less similar to other *D. huoshanense* sequences. |
| ITS2 | AF355569 | *D. huoshanense* | Only 95.88% or less similar to other *D. huoshanense* sequences. |
| chloroplast trnL | LC193515 | *D. jenkinsii* | Only 94.81% or less similar to other *D. jenkinsii* sequences. Best match to LC193517 *D. lindleyi* at 96.09% similarity. |
| ITS1 | EF629321 | *D. jenkinsii* | Not match to other *D. jenkinsii* sequences. Best match to several sequences of *D. acinaciforme* and *D. spatella* at 100% similarity. |
| ITS2 | EF629321 | *D. jenkinsii* | Not match to other *D. jenkinsii* sequences. Best match to several sequences of *D. acinaciforme* and *D. spatella* at 100% similarity. |
| ITS2 | KY745860 | *D. jenkinsii* | Only 92.00% or less similar to other *D. jenkinsii* sequences. Best match to multiple *D. lindleyi* sequences at 100% similarity. |
| matK | FJ216660 | *D. jenkinsii* | Not match to other *D. jenkinsii* sequences. Best match to KY062488 *D. sp.* and several sequences of *D. primulinum* at 100% similarity. |
| psbA-trnH | KF177517 | *D. jenkinsii* | Not match to other *D. jenkinsii* sequences. Best match to KF177525. *D. menglaense* at 99.87% similarity. |
| trnL | KC568314 | *D. jenkinsii* | Only 94.81% or less similar to other *D. jenkinsii* sequences. Best match to multiple *D. lindleyi* sequences at 99.19% similarity. |
| psbA-trnH | EF590688 | *D. lindleyi* | Same sample voucher number as GQ24286. One of them should be wrong. |
| psbA-trnH | GQ248286 | *D. lindleyi* | Same sample voucher number as EF590688. One of them should be wrong. |
| rbcL | EF590520 | *D. lindleyi* | Not match to any *Dendrobium* sequences. Best match to MK451849 *Ludisa discolor* at 99.57% similarity. |
| ITS1 | HQ114258 | *D. lituiflorum* | Only 95.15% or less similar to other *D. lituiflorum* sequences. Best match to KF143465 *D. gratiosissimum* at 100% similarity. |
| ITS1 | KX792015 | *D. lituiflorum* | Not match to any other *D. lituiflorum* sequences. Best match to KX792017 *D. candidum* at 100% similarity. |
| ITS2 | HQ114258 | *D. lituiflorum* | Only 91.87% or less similar to other *D. lituiflorum* sequences. Best match to MT004863 *D. devonianum* at 100% similarity. |
| ITS2 | KX792015 | *D. lituiflorum* | Not match to any other *D. lituiflorum* sequences. Best match to KY499214 *D. stuposum* at 99.18% similarity. |
| ITS2 | KY745806 | *D. lituiflorum* | Only 91.46% or less similar to other *D. lituiflorum* sequences. Best match to several *D. aphyllum* sequences at 100% similarity. |
| ITS2 | KY745807 | *D. lituiflorum* | Only 97.58% or less similar to other *D. lituiflorum* sequences. Best match to several *D. anosmum* sequences at 100% similarity. |
| ITS1 | AY485703 | *D. loddigesii* | Only 92.54% or less similar to other *D. loddigesii* sequences. |
| ITS1 | KJ672683 | *D. loddigesii* | Only 94.42% or less similar to other *D. loddigesii* sequences. |
| ITS2 | AY485703 | *D. loddigesii* | Only 97.93% or much less (91.09%) similar to other *D. loddigessi* sequences. |
| ITS2 | KJ672683 | *D. loddigesii* | Only 97.93% or much less (92.59%) similar to other *D. loddigessi* sequences. |
| trnL | EF397927 | *D. loddigesii* | Not match to other D. loddigessi sequences. Best match to LC192810 *D. primuilnum* at 97.13% similarity. |
| ITS1 | DQ058800 | *D. minutiflorum* | Only 92.67% or less similar to other *D. minutiflorum* sequences. Best match to KF143445 *D. compactum* at 100% similarity. |
| ITS2 | DQ058800 | *D. minutiflorum* | Only 92.92% or less similar to other *D. minutiflorum* sequences. Best match to KF143445 *D. compactum* at 100% similarity. |
| chloroplast psbA-trnH | AB893950 | *D. moniliforme* | Only 97.84% or less similar to other *D. moniliforme* sequences. |
| ITS1 | HM590369 | *D. moniliforme* | Not match to other *D. moniliforme* sequences. Best match to KY062487 *D. sp.* and several *D. parishii* sequences at 100% similarity. |
| ITS1 | AY485718 | *D. moniliforme* | Only 94.25% or less similar to other *D. moniliforme* sequences. |
| ITS2 | HM590369 | *D. moniliforme* | Not match to other *D. moniliforme* sequences. Best match to KY062487 *D. sp*., KY745844.1 *D. williamsonii* and multiple sequences of *D. parishii* at 100% similarity. |
| ITS2 | AY485718 | *D. moniliforme* | Only 95.08% or less similar to other *D. moniliforme* sequences. |
| psbA-trnH | KP412174 | *D. moniliforme* | Only 98.19% or less similar to other *D. moniliform*e sequences. Best match to KJ174261. *D. fanjingshanense* at 99.61% similarity. |
| rbcL | DQ449475 | *D. moniliforme* | Not match to other *D. moniliforme* sequences. Best match to KY635881 *Litchi chinensis* and DQ449472 *D. crumenatum* at 100% similarity. |
| trnL | KF143595 | *D. moniliforme* | Only 96.96% or less similar to other *D. moniliforme* sequences. |
| ITS1 | KF143492 | *D. moschatum* | Only 85.71% or less similar to other *D. moschatum* sequences. Best match to several *D. pulchellum* sequences at 100%. |
| ITS1 | KY499229 | *D. moschatum* | Only 96.15% or less similar to other *D. moschatum* sequences. |
| ITS1 | AY485695 | *D. moschatum* | Not match to any other sequences except for itself. |
| ITS2 | KF143492 | *D. moschatum* | Only 82.23% or less match to other *D. moschaum* sequences. Best match to several *D. pulchellum* sequences at 100%. |
| ITS2 | AY485695 | *D. moschatum* | Only 93.06% or less match to other *D. moschaum* sequences. |
| ITS2 | KY745859 | *D. moschatum* | Not match to other *D. moschaum* sequences. Best match to MK522191 *D. tuposum*., KY745823.1 *D. hainanense* and multiple sequences of *D. fimbriatum* at 100% similarity. |
| chloroplast psbA-trnH | KX377961 | *D. nobile* | Not match to any other *D. nobile* sequences. Best match to several sequences of *D. catenatum* at 99.87% similarity. |
| chloroplast trnL | KX377961 | *D. nobile* | Only 94.38% or less similar to other *D. nobile* sequences. Best match to KJ862886 *D. officinale* at 98.72% similarity. |
| chloroplast trnL-trnF | KX377961 | *D. nobile* | Only 96.72% or less similar to other *D. nobile* sequences. Best match to multiple sequences of *D. officinale* at 99.22% similarity. |
| ITS1 | KY966564 | *D. nobile* | Only 90.09% or less match to other *D. nobile* sequences. |
| ITS1 | KX600497 | *D. nobile* | Not match to any other *D. nobile* sequences. Best match to several sequences of *D. wardianum* at 100% similarity. |
| ITS1 | EU477507 | *D. nobile* | Only 92.34% or less match to other *D. nobile* sequences. Best match to AB593662 *D. signatum* at 99.15% similarity. |
| ITS1 | AY485719 | *D. nobile* | Only 94.67% or less match to other *D. nobile* sequences. |
| ITS2 | KX600497 | *D. nobile* | Not match to any other *D. nobile* sequences. Best match to several sequences of *D. wardianum* at 100% similarity. |
| ITS2 | AY485719 | *D. nobile* | Only 91.80% or less match to other *D. nobile* sequences. |
| psbA-trnH | EF590687 | *D. nobile* | Query coverage to other matched *D. nobile* sequences is only 92%. Best match to KP412231 *D. crepidatum* at 93.30% similarity. |
| ITS1 | AY485692 | *D. officinale* | Only 96.89% or less similar to other *D. officinale* sequences. |
| ITS1 | AF401490 | *D. officinale* | Not match to any other *D. officinale* sequences. Best match to KJ210478 *D. officinale* at 99.57% similarity. |
| ITS1 | MH031756 | *D. officinale* | 100% similar to MH031756-MH031761. But all are 95% or less similar to other *D. officinale* sequences. Best match to multiple sequences of *D. nobile* at 100% similarity. |
| ITS1 | MH031757 | *D. officinale* | 100% similar to MH031756-MH031761. But all are 95% or less similar to other *D. officinale* sequences. Best match to multiple sequences of *D. nobile* at 100% similarity. |
| ITS1 | MH031758 | *D. officinale* | 100% similar to MH031756-MH031761. But all are 95% or less similar to other *D. officinale* sequences. Best match to multiple sequences of *D. nobile* at 100% similarity. |
| ITS1 | MH031759 | *D. officinale* | 100% similar to MH031756-MH031761. But all are 95% or less similar to other *D. officinale* sequences. Best match to multiple sequences of *D. nobile* at 100% similarity. |
| ITS1 | MH031760 | *D. officinale* | 100% similar to MH031756-MH031761. But all are 95% or less similar to other *D. officinale* sequences. Best match to multiple sequences of *D. nobile* at 100% similarity. |
| ITS1 | MH031761 | *D. officinale* | 100% similar to MH031756-MH031761. But all are 95% or less similar to other *D. officinale* sequences. Best match to multiple sequences of *D. nobile* at 100% similarity. |
| ITS2 | AF401490 | *D. officinale* | Only 96.14% or less similar to other *D. officinale* sequences. Best match to KY508531 *D. moniliforme* at 99.14% similarity. |
| ITS2 | MH031756 | *D. officinale* | 100% similar to MH031756-MH031761. But all are 98% or less similar to other *D. officinale* sequences. Best match to multiple sequences of *D. nobile* at 100% similarity. |
| ITS2 | MH031757 | *D. officinale* | 100% similar to MH031756-MH031761. But all are 98% or less similar to other *D. officinale* sequences. Best match to multiple sequences of *D. nobile* at 100% similarity. |
| ITS2 | MH031758 | *D. officinale* | 100% similar to MH031756-MH031761. But all are 98% or less similar to other *D. officinale* sequences. Best match to multiple sequences of *D. nobile* at 100% similarity. |
| ITS2 | MH031759 | *D. officinale* | 100% similar to MH031756-MH031761. But all are 98% or less similar to other *D. officinale* sequences. Best match to multiple sequences of *D. nobile* at 100% similarity. |
| ITS2 | MH031760 | *D. officinale* | 100% similar to MH031756-MH031761. But all are 98% or less similar to other *D. officinale* sequences. Best match to multiple sequences of *D. nobile* at 100% similarity. |
| ITS2 | MH031761 | *D. officinale* | 100% similar to MH031756-MH031761. But all are 98% or less similar to other *D. officinale* sequences. Best match to multiple sequences of *D. nobile* at 100% similarity. |
| ITS2 | KY745843 | *D. parishii* | Not match to any other *D. parisihii* sequences. Best match to multiple sequences of *D. williamsonii* and *D. cariniferum* at 100% similarity. |
| chloroplast psbA-trnH | KT695604 | *D. pendulum* | Not match to any other *D. pendulum* sequences. Best match to LC192957 *D. falconeri* at 99.70% similarity. |
| chloroplast trnL | KT695604 | *D. pendulum* | Not match to any other *D. pendulum* sequences. Best match to LC192957 *D. falconeri* at 100% similarity. |
| ITS1 | AY485712 | *D. pendulum* | Only 92.92% or less similar to other *D. pendulum* sequences. |
| ITS2 | AY485712 | *D. pendulum* | Only 93.78% or less similar to other *D. pendulum* sequences. |
| trnL | KF143603 | *D. pendulum* | Not match to any other *D. pendulum* sequences. Best match to KF143607 *D. porphyrochilum* at 99.62% similarity. |
| chloroplast psbA-trnH | LC192810 | *D. primulinum* | Not match to any other *D. primulinum* sequences. Best match to LC192953 *D. aphyllum* at 100% similarity. |
| ITS1 | AY485715 | *D. primulinum* | Only 92.79% or less similar to other *D. primulinum* sequences. |
| ITS2 | AY485715 | *D. primulinum* | Only 91.84% or less similar to other *D. primulinum* sequences. Best match to AY485708 *D. falconeri* at 92.65% similarity. |
| matK | AB972317 | *D. primulinum* | Not match to any other *D. primulinum* sequences. Best match to multiple sequences of *D. crystallinum* at 100% similarity. |
| psbA-trnH | KP704452 | *D. primulinum* | Only 97.59% or less similar to other *D. primulinum* sequences. Best match to KF177470 *D. bellatulum* and multiple sequences of *D. aduncum* at 100% similarity. |
| trnL | KF143605 | *D. primulinum* | Only 97.32% or less similar to other *D. primulinum* sequences. Best match to EF397927 *D. loddigesii* at 100% similarity. |
| ITS1 | KC205203 | *D. scoriarum* | Not match to any other *D. scoriarum* sequences. Best match to KC205191 *D. chrysanthum* at 100% similarity. |
| ITS1 | GU339108 | *D. scoriarum* | Only 84.68% or less similar to other *D. scoriarum* sequences. Best match to GU339108 *D. guangxiense* at 100% similarity. |
| ITS2 | KC205203 | *D. scoriarum* | Not match to any other *D. scoriarum* sequences. Best match to multiple sequences of *D. loddigesii* at 100% similarity. |
| ITS2 | GU339108 | *D. scoriarum* | Best match to GU339108 *D. guangxiense* at 100% similarity. |
| trnL | KC568315 | *D. scoriarum* | Only 95.49% or less similar to other *D. scoriarum* sequences. Best match to LC348722 *D. shixingense* at 97.61% similarity. |
| chloroplast matK | KR673323 | *D. strongylanthum* | Not match to any other *D. strongylanthum* sequences. Best match to KR296655 *Alocasia macrorrhizos* at 99.61% similarity. |
| chloroplast psbA-trnH | KR673323 | *D. strongylanthum* | Not match to any other *D. strongylanthum* sequences. Best match to KR296655 *Alocasia macrorrhizos* at 99.66% similarity. |
| chloroplast rbcL | KR673323 | *D. strongylanthum* | Not match to any other *D. strongylanthum* sequences. Best match to KR296655 *Alocasia macrorrhizos* at 100% similarity. |
| chloroplast trnL | KR673323 | *D. strongylanthum* | Only 94.06% or less similar to other *D. strongylanthum* sequences. Best match to KR296655 *Alocasia macrorrhizos* at 100% similarity. |
| ITS1 | KC568296 | *D. strongylanthum* | Not match to any other *D. strongylanthum* sequences. Best match to multiple sequences of *D. moschatum* at 98.71% similarity. |
| ITS2 | KC568296 | *D. strongylanthum* | Not match to any other *D. strongylanthum* sequences. Best match to multiple sequences of *D. moschatum* at 100% similarity. |
| psbA-trnH | KF177553 | *D. strongylanthum* | Not match to any other *D. strongylanthum* sequences. Best match to KF177491 *D. denudans* at 99.87% similarity. |
| trnL | KP749362 | *D. strongylanthum* | Only 96.64% or less similar to other *D. strongylanthum* sequences. |
| trnL | KC568309 | *D. strongylanthum* | Not match to any other *D. strongylanthum* sequences. Best match to EF397911 *D. brymerianum* at 98.48% similarity. |
| trnL | KF143621 | *D. strongylanthum* | 99.88% similar to KF143620 *D. strongylanthum*. But both are only 96.51% or less similar to other *D. strongylanthum* sequences. Best match to KF143558 *D. denudans* at 99.88% similarity. |
| trnL | KF143620 | *D. strongylanthum* | 99.88% similar to KF143621 *D. strongylanthum*. But both are only 96.51% or less similar to other *D. strongylanthum* sequences. Best match to KF143558 *D. denudans* at 99.88% similarity. |
| ITS1 | AY485702 | *D. thyrsiflorum* | Not match to any other *D. thyrsiflorum* sequences. |
| ITS1 | KC205200 | *D. thyrsiflorum* | Only 91.77% or less similar to other *D. thyrsiflorum* sequences. Best match to KC205192 *D.* hybrid cultivar at 99.57% similarity. |
| ITS2 | AY485702 | *D. thyrsiflorum* | Not match to any other *D. thyrsiflorum* sequences. |
| ITS2 | KC205200 | *D. thyrsiflorum* | Only 92.47% or less similar to other *D. thyrsiflorum* sequences. Best match to MK522209 *D. amabile* at 100% similarity. |
| trnL | EF397933 | *D. thyrsiflorum* | Best match to LC189343 *Bulbophyllum praetervisum* and LC189340 *Bulbophyllum macranthum* at 97.58% similarity |
| trnL-trnF | EF397933 | *D. thyrsiflorum* | Best match to LC189343 *Bulbophyllum praetervisum* and LC189340 *Bulbophyllum macranthum* at 97.58% similarity |
| psbA-trnH | KJ672728 | *D. tosaense* | Not match to any other *D. tosaense* sequences. Best match to multiple sequences of *D. catenatum* and *D. officinale* at 100% similarity. |
| ITS1 | KX600500 | *D. wardianum* | Not match to any other *D. wardianum* sequences. Best match to multiple sequences of *D. nobile* at 100% similarity. |
| ITS2 | KX600500 | *D. wardianum* | Not match to any other *D. wardianum* sequences. Best match to multiple sequences of *D. nobile* at 100% similarity. |
| psbA-trnH | KT792700 | *D. wardianum* | Not match to any other *D. wardianum* sequences. Best match to GQ153536 *D. gratiosissimum* and FJ216477 *D. lituiflorum* at 98.47% similarity. |
| trnL-trnF | EF397931 | *D. wardianum* | Only 91.39% similar to the other *D. thyrsiflorum* sequence. Best match to EF397914 *D. chrysanthum* at 96.16% similarity. |
