# Supplementary figures and images for: Assessing the reliability of medicinal Dendrobium sequences in GenBank for botanical species identification
Source: Sci Rep. 2021 Feb 9;11:3439. doi: 10.1038/s41598-021-82385-z (PMC7873228; doi:10.1038/s41598-021-82385-z)

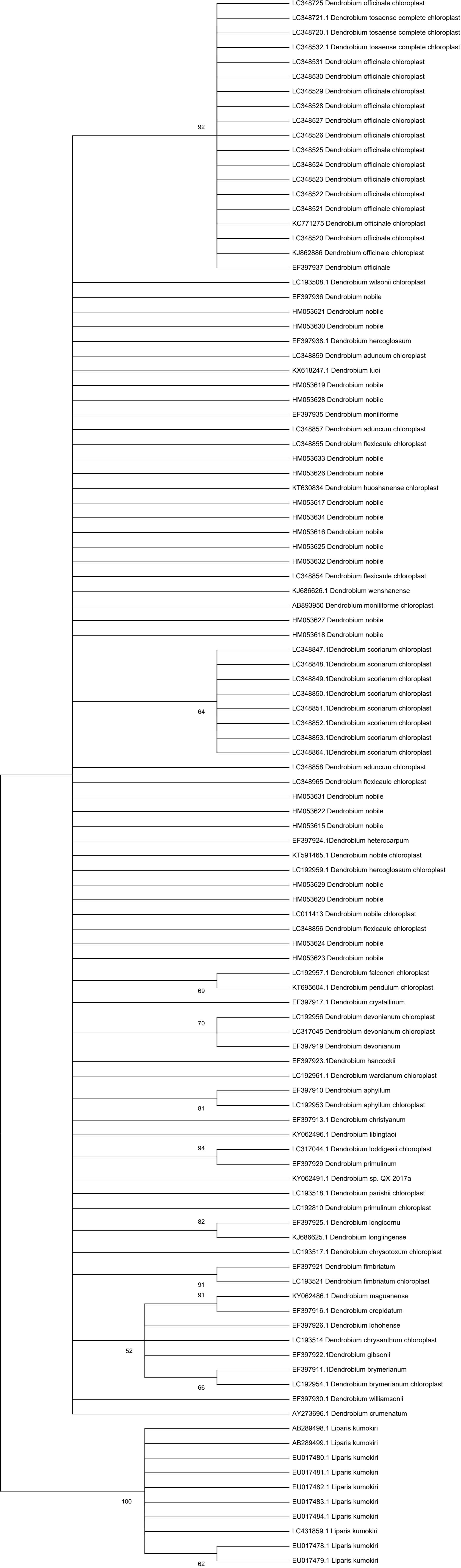

Supplement: Supplementary file 6 — Supplementary Figure S4. [file 41598_2021_82385_MOESM6_ESM.pdf]

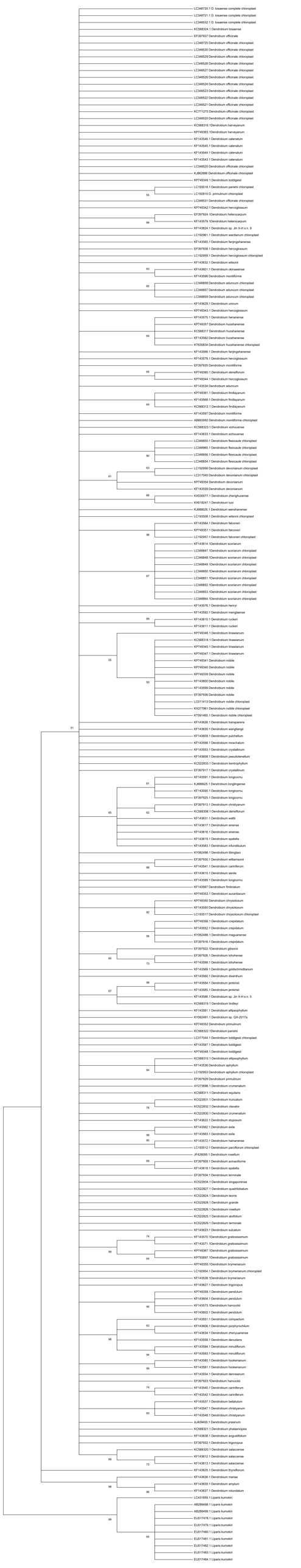

Supplement: Supplementary file 7 — Supplementary Figure S5. [file 41598_2021_82385_MOESM7_ESM.pdf]
