# Supplementary material for: Assessing the reliability of medicinal Dendrobium sequences in GenBank for botanical species identification
Source: Sci Rep. 2021 Feb 9;11:3439. doi: 10.1038/s41598-021-82385-z (PMC7873228; doi:10.1038/s41598-021-82385-z)
Supplement: Supplementary file 4 — Supplementary Figure S2. [file 41598_2021_82385_MOESM4_ESM.pdf]

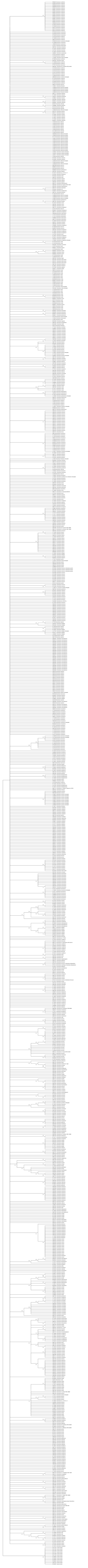
KP686307 Dendrobium moniliforme
KF262075 Dendrobium moniliforme
KP686308 Dendrobium moniliforme
KP686309 Dendrobium moniliforme
KP686310 Dendrobium moniliforme
KP686311 Dendrobium moniliforme
KP686312 Dendrobium moniliforme
KP686313 Dendrobium moniliforme
KP686314 Dendrobium moniliforme
KP686315 Dendrobium moniliforme
KP686316 Dendrobium moniliforme
KP686317 Dendrobium moniliforme
KP686318 Dendrobium moniliforme
KP686319 Dendrobium moniliforme
KP686320 Dendrobium moniliforme
KC704627 Dendrobium moniliforme
KC704628 Dendrobium moniliforme
AB519773 Dendrobium moniliforme
AB519774 Dendrobium moniliforme
AB519775 Dendrobium moniliforme
KP704463 Dendrobium huoshanense
KP704465 Dendrobium huoshanense
KP704464 Dendrobium huoshanense
KJ187340 Dendrobium moniliforme
KF143699 Dendrobium moniliforme
AB847816 Dendrobium moniliforme
KT630834 Dendrobium huoshanense chloroplast
KF143686 Dendrobium huoshanense
LC086430 Dendrobium huoshanense
KJ187337.1Dendrobium heterocarpum
AB847823.1 Dendrobium okinawense
KF143704.1 Dendrobium okinawense
KJ187332.1 Dendrobium henanense
KF143679.1 Dendrobium henanense
KJ686620.1 Dendrobium wenshanense
KJ187347 Dendrobium moniliforme
KJ187346 Dendrobium moniliforme
LC193508.1 Dendrobium wilsonii chloroplast
KP159294 Dendrobium moniliforme
KR075044.1 Dendrobium wilsonii
KF143698 Dendrobium moniliforme
KJ187345 Dendrobium moniliforme
KT778741 Dendrobium devonianum
AB847684.1 Dendrobium aff. moniliforme TBG 124453
KJ187348 Dendrobium moniliforme
KJ187343 Dendrobium moniliforme
KJ187342 Dendrobium moniliforme
KJ187341 Dendrobium moniliforme
KF361618 Dendrobium moniliforme
FJ216654 Dendrobium moniliforme
AB893950 Dendrobium moniliforme chloroplast
KF143700 Dendrobium moniliforme
KJ187344 Dendrobium moniliforme
KP704462 Dendrobium moniliforme
KP704461 Dendrobium moniliforme
HM055147 Dendrobium aduncum
FJ794061.1 Dendrobium flexicaule
LC348965 Dendrobium flexicaule chloroplast
LC348856 Dendrobium flexicaule chloroplast
LC348854 Dendrobium flexicaule chloroplast
LC348855 Dendrobium flexicaule chloroplast
AB847713.1 Dendrobium catenatum
KF143650.1 Dendrobium catenatum
GU565191.1 Dendrobium aurantiacum
AF448861.1 Dendrobium chryseum
MG324296.1 Dendrobium catenatum
MG490215.1 Dendrobium catenatum
MG490207.1 Dendrobium catenatum
MG760737 Dendrobium officinale
GU569960.1 Dendrobium dixanthum
EF221847 Dendrobium officinale
MG760736 Dendrobium officinale
LC348525 Dendrobium officinale chloroplast
AB519770.1 Dendrobium tosaense
AB519771.1 Dendrobium tosaense
KP159295.1 Dendrobium candidum
KF143649.1 Dendrobium catenatum
KF143647.1 Dendrobium catenatum
KF143648.1 Dendrobium catenatum
MG490212.1 Dendrobium catenatum
FJ794050 Dendrobium officinale
FJ794049 Dendrobium officinale
FJ794048 Dendrobium officinale
FJ794047 Dendrobium officinale
FJ794043 Dendrobium officinale
FJ794046 Dendrobium officinale
GU181388 Dendrobium officinale
FJ216653 Dendrobium officinale
KJ862886 Dendrobium officinale chloroplast
LC348520 Dendrobium officinale chloroplast
KC771275 Dendrobium officinale chloroplast
LC348521 Dendrobium officinale chloroplast
