# Supplementary material for: Assessing the reliability of medicinal Dendrobium sequences in GenBank for botanical species identification
Source: Sci Rep. 2021 Feb 9;11:3439. doi: 10.1038/s41598-021-82385-z (PMC7873228; doi:10.1038/s41598-021-82385-z)

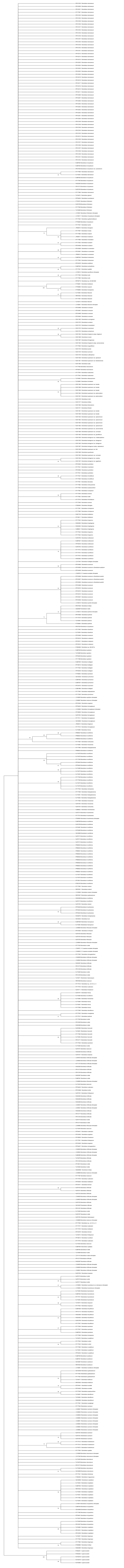

Supplement: Supplementary file 5 — Supplementary Figure S3. [file 41598_2021_82385_MOESM5_ESM.pdf]
