# Supplementary material for: Assessing the reliability of medicinal Dendrobium sequences in GenBank for botanical species identification
Source: Sci Rep. 2021 Feb 9;11:3439. doi: 10.1038/s41598-021-82385-z (PMC7873228; doi:10.1038/s41598-021-82385-z)
Supplement: Supplementary file 8 — Supplementary Figure S6. [file 41598_2021_82385_MOESM8_ESM.pdf]

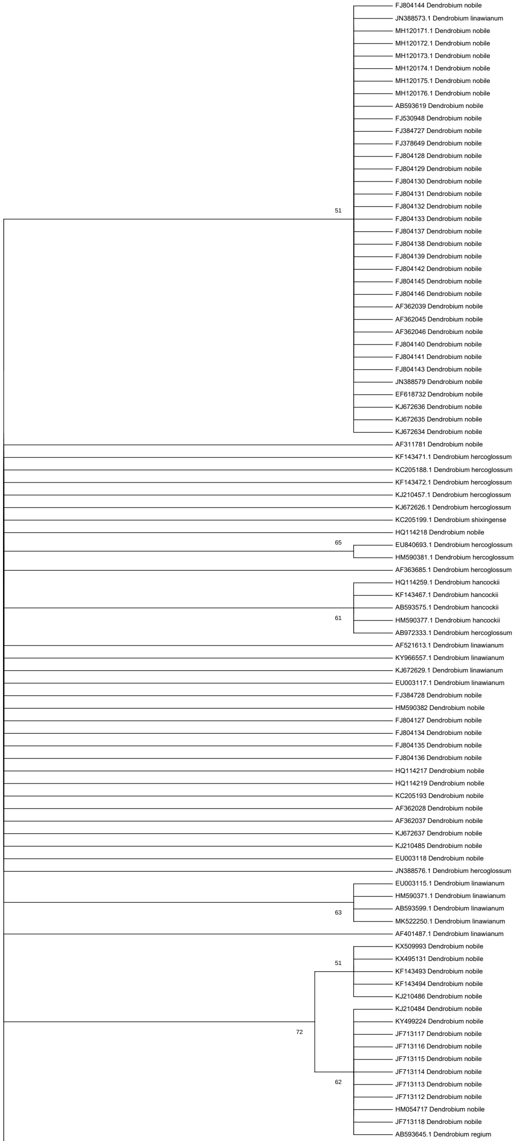
FJ804144 Dendrobium nobile
JN388573.1 Dendrobium linawianum
MH120171.1 Dendrobium nobile
MH120172.1 Dendrobium nobile
MH120173.1 Dendrobium nobile
MH120174.1 Dendrobium nobile
MH120175.1 Dendrobium nobile
MH120176.1 Dendrobium nobile
AB593619 Dendrobium nobile
FJ530948 Dendrobium nobile
FJ384727 Dendrobium nobile
FJ378649 Dendrobium nobile
FJ804128 Dendrobium nobile
FJ804129 Dendrobium nobile
FJ804130 Dendrobium nobile
FJ804131 Dendrobium nobile
FJ804132 Dendrobium nobile
51
FJ804133 Dendrobium nobile
FJ804137 Dendrobium nobile
FJ804138 Dendrobium nobile
FJ804139 Dendrobium nobile
FJ804142 Dendrobium nobile
FJ804145 Dendrobium nobile
FJ804146 Dendrobium nobile
AF362039 Dendrobium nobile
AF362045 Dendrobium nobile
AF362046 Dendrobium nobile
FJ804140 Dendrobium nobile
FJ804141 Dendrobium nobile
FJ804143 Dendrobium nobile
JN388579 Dendrobium nobile
EF618732 Dendrobium nobile
KJ672636 Dendrobium nobile
KJ672635 Dendrobium nobile
KJ672634 Dendrobium nobile
AF311781 Dendrobium nobile
KF143471.1 Dendrobium hercoglossum
KC205188.1 Dendrobium hercoglossum
KF143472.1 Dendrobium hercoglossum
KJ210457.1 Dendrobium hercoglossum
KJ672626.1 Dendrobium hercoglossum
KC205199.1 Dendrobium shixingense
HQ114218 Dendrobium nobile
65
EU840693.1 Dendrobium hercoglossum
HM590381.1 Dendrobium hercoglossum
AF363685.1 Dendrobium hercoglossum
HQ114259.1 Dendrobium hancockii
KF143467.1 Dendrobium hancockii
AB593575.1 Dendrobium hancockii
61
HM590377.1 Dendrobium hancockii
AB972333.1 Dendrobium hercoglossum
AF521613.1 Dendrobium linawianum
KY966557.1 Dendrobium linawianum
KJ672629.1 Dendrobium linawianum
EU003117.1 Dendrobium linawianum
FJ384728 Dendrobium nobile
HM590382 Dendrobium nobile
FJ804127 Dendrobium nobile
FJ804134 Dendrobium nobile
FJ804135 Dendrobium nobile
FJ804136 Dendrobium nobile
HQ114217 Dendrobium nobile
HQ114219 Dendrobium nobile
KC205193 Dendrobium nobile
AF362028 Dendrobium nobile
AF362037 Dendrobium nobile
KJ672637 Dendrobium nobile
KJ210485 Dendrobium nobile
EU003118 Dendrobium nobile
JN388576.1 Dendrobium hercoglossum
EU003115.1 Dendrobium linawianum
HM590371.1 Dendrobium linawianum
AB593599.1 Dendrobium linawianum
63
MK522250.1 Dendrobium linawianum
AF401487.1 Dendrobium linawianum
KX509993 Dendrobium nobile
51
KX495131 Dendrobium nobile
KF143493 Dendrobium nobile
KF143494 Dendrobium nobile
KJ210486 Dendrobium nobile
KJ210484 Dendrobium nobile
KY499224 Dendrobium nobile
72
JF713117 Dendrobium nobile
JF713116 Dendrobium nobile
JF713115 Dendrobium nobile
JF713114 Dendrobium nobile
62
JF713113 Dendrobium nobile
JF713112 Dendrobium nobile
HM054717 Dendrobium nobile
JF713118 Dendrobium nobile
AB593645.1 Dendrobium regium

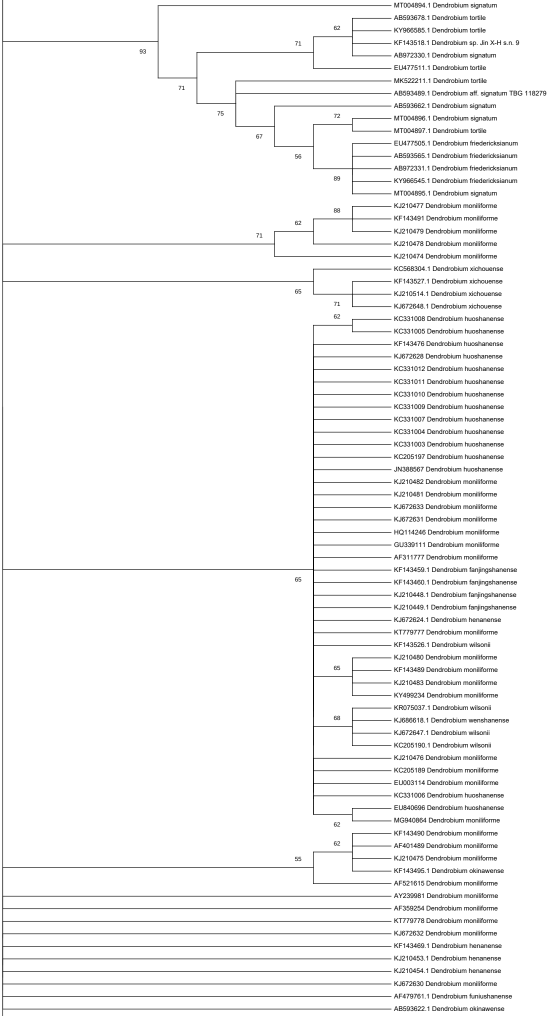

MT004894.1 Dendrobium signatum
AB593678.1 Dendrobium tortile
62
KY966585.1 Dendrobium tortile
71
KF143518.1 Dendrobium sp. Jin X-H s.n. 9
93
AB972330.1 Dendrobium signatum
EU477511.1 Dendrobium tortile
MK522211.1 Dendrobium tortile
71
AB593489.1 Dendrobium aff. signatum TBG 118279
AB593662.1 Dendrobium signatum
75
72
MT004896.1 Dendrobium signatum
MT004897.1 Dendrobium tortile
67
EU477505.1 Dendrobium friedericksianum
AB593565.1 Dendrobium friedericksianum
56
AB972331.1 Dendrobium friedericksianum
89
KY966545.1 Dendrobium friedericksianum
MT004895.1 Dendrobium signatum
KJ210477 Dendrobium moniliforme
88
KF143491 Dendrobium moniliforme
62
KJ210479 Dendrobium moniliforme
71
KJ210478 Dendrobium moniliforme
KJ210474 Dendrobium moniliforme
KC568304.1 Dendrobium xichouense
KF143527.1 Dendrobium xichouense
65
KJ210514.1 Dendrobium xichouense
71
KJ672648.1 Dendrobium xichouense
62
KC331008 Dendrobium huoshanense
KC331005 Dendrobium huoshanense
KF143476 Dendrobium huoshanense
KJ672628 Dendrobium huoshanense
KC331012 Dendrobium huoshanense
KC331011 Dendrobium huoshanense
KC331010 Dendrobium huoshanense
KC331009 Dendrobium huoshanense
KC331007 Dendrobium huoshanense
KC331004 Dendrobium huoshanense
KC331003 Dendrobium huoshanense
KC205197 Dendrobium huoshanense
JN388567 Dendrobium huoshanense
KJ210482 Dendrobium moniliforme
KJ210481 Dendrobium moniliforme
KJ672633 Dendrobium moniliforme
KJ672631 Dendrobium moniliforme
HQ114246 Dendrobium moniliforme
GU339111 Dendrobium moniliforme
AF311777 Dendrobium moniliforme
KF143459.1 Dendrobium fanjingshanense
65
KF143460.1 Dendrobium fanjingshanense
KJ210448.1 Dendrobium fanjingshanense
KJ210449.1 Dendrobium fanjingshanense
KJ672624.1 Dendrobium henanense
KT779777 Dendrobium moniliforme
KF143526.1 Dendrobium wilsonii
KJ210480 Dendrobium moniliforme
65
KF143489 Dendrobium moniliforme
KJ210483 Dendrobium moniliforme
KY499234 Dendrobium moniliforme
KR075037.1 Dendrobium wilsonii
68
KJ686618.1 Dendrobium wenshanense
KJ672647.1 Dendrobium wilsonii
KC205190.1 Dendrobium wilsonii
KJ210476 Dendrobium moniliforme
KC205189 Dendrobium moniliforme
EU003114 Dendrobium moniliforme
KC331006 Dendrobium huoshanense
EU840696 Dendrobium huoshanense
MG940864 Dendrobium moniliforme
62
KF143490 Dendrobium moniliforme
62
AF401489 Dendrobium moniliforme
55
KJ210475 Dendrobium moniliforme
KF143495.1 Dendrobium okinawense
AF521615 Dendrobium moniliforme
AY239981 Dendrobium moniliforme
AF359254 Dendrobium moniliforme
KT779778 Dendrobium moniliforme
KJ672632 Dendrobium moniliforme
KF143469.1 Dendrobium henanense
KJ210453.1 Dendrobium henanense
KJ210454.1 Dendrobium henanense
KJ672630 Dendrobium moniliforme
AF479761.1 Dendrobium funiushanense
AB593622.1 Dendrobium okinawense

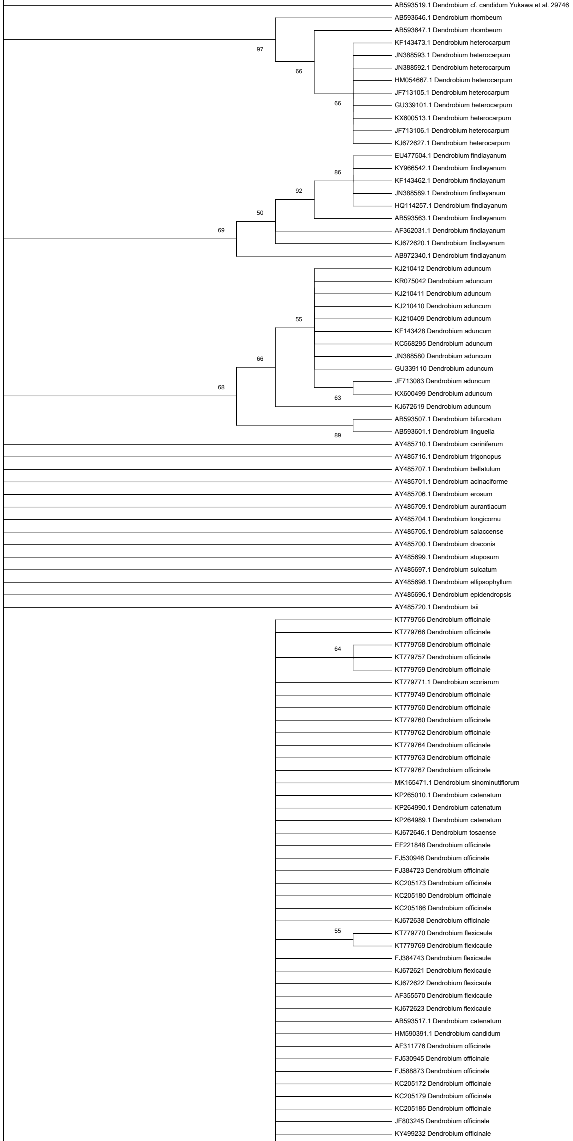
AB593519.1 Dendrobium cf. candidum Yukawa et al. 29746
AB593646.1 Dendrobium rhombeum
AB593647.1 Dendrobium rhombeum
97
KF143473.1 Dendrobium heterocarpum
JN388593.1 Dendrobium heterocarpum
JN388592.1 Dendrobium heterocarpum
66
HM054667.1 Dendrobium heterocarpum
JF713105.1 Dendrobium heterocarpum
66
GU339101.1 Dendrobium heterocarpum
KX600513.1 Dendrobium heterocarpum
JF713106.1 Dendrobium heterocarpum
KJ672627.1 Dendrobium heterocarpum
EU477504.1 Dendrobium findlayanum
KY966542.1 Dendrobium findlayanum
86
KF143462.1 Dendrobium findlayanum
92
JN388589.1 Dendrobium findlayanum
HQ114257.1 Dendrobium findlayanum
50
AB593563.1 Dendrobium findlayanum
69
AF362031.1 Dendrobium findlayanum
KJ672620.1 Dendrobium findlayanum
AB972340.1 Dendrobium findlayanum
KJ210412 Dendrobium aduncum
KR075042 Dendrobium aduncum
KJ210411 Dendrobium aduncum
KJ210410 Dendrobium aduncum
55
KJ210409 Dendrobium aduncum
KF143428 Dendrobium aduncum
KC568295 Dendrobium aduncum
66
JN388580 Dendrobium aduncum
GU339110 Dendrobium aduncum
68
JF713083 Dendrobium aduncum
KX600499 Dendrobium aduncum
63
KJ672619 Dendrobium aduncum
AB593507.1 Dendrobium bifurcatum
AB593601.1 Dendrobium linguella
89
AY485710.1 Dendrobium cariniferum
AY485716.1 Dendrobium trigonopus
AY485707.1 Dendrobium bellatulum
AY485701.1 Dendrobium acinaciforme
AY485706.1 Dendrobium erosum
AY485709.1 Dendrobium aurantiacum
AY485704.1 Dendrobium longicornu
AY485705.1 Dendrobium salaccense
AY485700.1 Dendrobium draconis
AY485699.1 Dendrobium stuposum
AY485697.1 Dendrobium sulcatum
AY485698.1 Dendrobium ellipsophyllum
AY485696.1 Dendrobium epidendropsis
AY485720.1 Dendrobium tsii
KT779756 Dendrobium officinale
KT779766 Dendrobium officinale
64
KT779758 Dendrobium officinale
KT779757 Dendrobium officinale
KT779759 Dendrobium officinale
KT779771.1 Dendrobium scoriarum
KT779749 Dendrobium officinale
KT779750 Dendrobium officinale
KT779760 Dendrobium officinale
KT779762 Dendrobium officinale
KT779764 Dendrobium officinale
KT779763 Dendrobium officinale
KT779767 Dendrobium officinale
MK165471.1 Dendrobium sinominutiflorum
KP265010.1 Dendrobium catenatum
KP264990.1 Dendrobium catenatum
KP264989.1 Dendrobium catenatum
KJ672646.1 Dendrobium tosaense
EF221848 Dendrobium officinale
FJ530946 Dendrobium officinale
FJ384723 Dendrobium officinale
KC205173 Dendrobium officinale
KC205180 Dendrobium officinale
KC205186 Dendrobium officinale
KJ672638 Dendrobium officinale
55
KT779770 Dendrobium flexicaule
KT779769 Dendrobium flexicaule
FJ384743 Dendrobium flexicaule
KJ672621 Dendrobium flexicaule
KJ672622 Dendrobium flexicaule
AF355570 Dendrobium flexicaule
KJ672623 Dendrobium flexicaule
AB593517.1 Dendrobium catenatum
HM590391.1 Dendrobium candidum
AF311776 Dendrobium officinale
FJ530945 Dendrobium officinale
FJ588873 Dendrobium officinale
KC205172 Dendrobium officinale
KC205179 Dendrobium officinale
KC205185 Dendrobium officinale
JF803245 Dendrobium officinale
KY499232 Dendrobium officinale

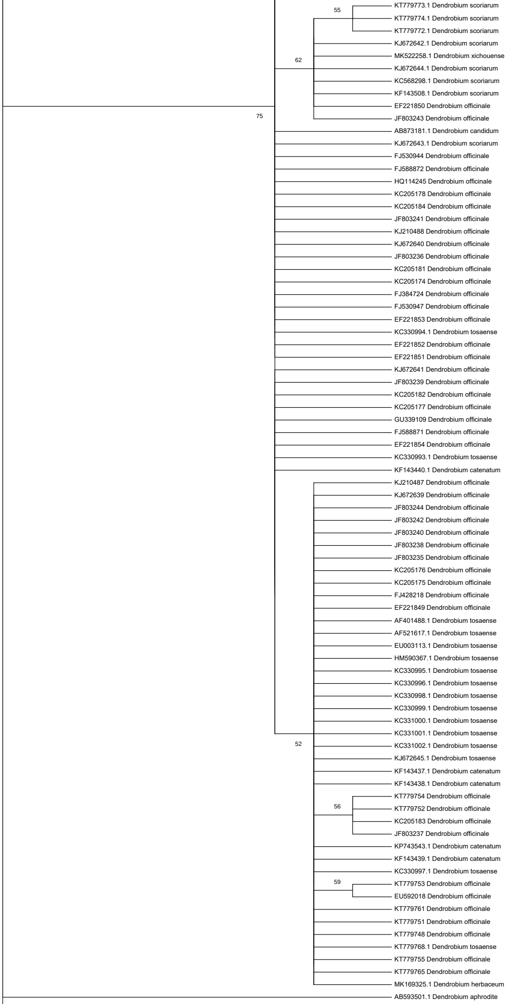

55
KT779773.1 Dendrobium scoriarum
KT779774.1 Dendrobium scoriarum
KT779772.1 Dendrobium scoriarum
KJ672642.1 Dendrobium scoriarum
MK522258.1 Dendrobium xichouense
62
KJ672644.1 Dendrobium scoriarum
KC568298.1 Dendrobium scoriarum
KF143508.1 Dendrobium scoriarum
EF221850 Dendrobium officinale
75
JF803243 Dendrobium officinale
AB873181.1 Dendrobium candidum
KJ672643.1 Dendrobium scoriarum
FJ530944 Dendrobium officinale
FJ588872 Dendrobium officinale
HQ114245 Dendrobium officinale
KC205178 Dendrobium officinale
KC205184 Dendrobium officinale
JF803241 Dendrobium officinale
KJ210488 Dendrobium officinale
KJ672640 Dendrobium officinale
JF803236 Dendrobium officinale
KC205181 Dendrobium officinale
KC205174 Dendrobium officinale
FJ384724 Dendrobium officinale
FJ530947 Dendrobium officinale
EF221853 Dendrobium officinale
KC330994.1 Dendrobium tosaense
EF221852 Dendrobium officinale
EF221851 Dendrobium officinale
KJ672641 Dendrobium officinale
JF803239 Dendrobium officinale
KC205182 Dendrobium officinale
KC205177 Dendrobium officinale
GU339109 Dendrobium officinale
FJ588871 Dendrobium officinale
EF221854 Dendrobium officinale
KC330993.1 Dendrobium tosaense
KF143440.1 Dendrobium catenatum
KJ210487 Dendrobium officinale
KJ672639 Dendrobium officinale
JF803244 Dendrobium officinale
JF803242 Dendrobium officinale
JF803240 Dendrobium officinale
JF803238 Dendrobium officinale
JF803235 Dendrobium officinale
KC205176 Dendrobium officinale
KC205175 Dendrobium officinale
FJ428218 Dendrobium officinale
EF221849 Dendrobium officinale
AF401488.1 Dendrobium tosaense
AF521617.1 Dendrobium tosaense
EU003113.1 Dendrobium tosaense
HM590367.1 Dendrobium tosaense
KC330995.1 Dendrobium tosaense
KC330996.1 Dendrobium tosaense
KC330998.1 Dendrobium tosaense
KC330999.1 Dendrobium tosaense
KC331000.1 Dendrobium tosaense
KC331001.1 Dendrobium tosaense
52
KC331002.1 Dendrobium tosaense
KJ672645.1 Dendrobium tosaense
KF143437.1 Dendrobium catenatum
KF143438.1 Dendrobium catenatum
KT779754 Dendrobium officinale
56
KT779752 Dendrobium officinale
KC205183 Dendrobium officinale
JF803237 Dendrobium officinale
KP743543.1 Dendrobium catenatum
KF143439.1 Dendrobium catenatum
KC330997.1 Dendrobium tosaense
59
KT779753 Dendrobium officinale
EU592018 Dendrobium officinale
KT779761 Dendrobium officinale
KT779751 Dendrobium officinale
KT779748 Dendrobium officinale
KT779768.1 Dendrobium tosaense
KT779755 Dendrobium officinale
KT779765 Dendrobium officinale
MK169325.1 Dendrobium herbaceum
AB593501.1 Dendrobium aphrodite

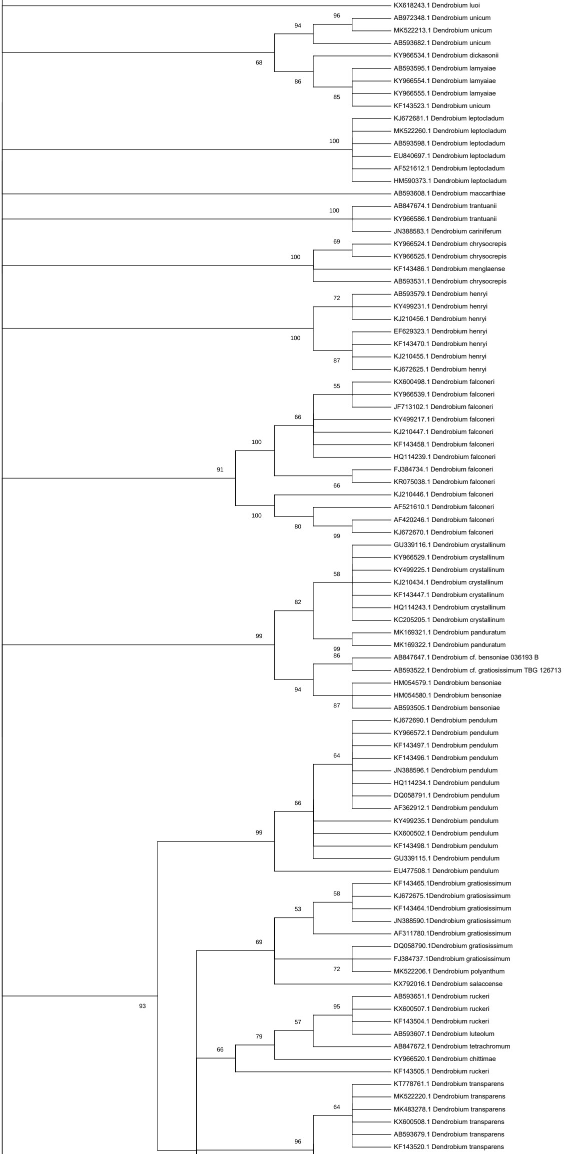
KX618243.1 Dendrobium luoi
96
AB972348.1 Dendrobium unicum
94
MK522213.1 Dendrobium unicum
AB593682.1 Dendrobium unicum
KY966534.1 Dendrobium dickasonii
68
AB593595.1 Dendrobium lamyaiae
86
KY966554.1 Dendrobium lamyaiae
KY966555.1 Dendrobium lamyaiae
85
KF143523.1 Dendrobium unicum
KJ672681.1 Dendrobium leptocladum
MK522260.1 Dendrobium leptocladum
100
AB593598.1 Dendrobium leptocladum
EU840697.1 Dendrobium leptocladum
AF521612.1 Dendrobium leptocladum
HM590373.1 Dendrobium leptocladum
AB593608.1 Dendrobium maccarthiae
100
AB847674.1 Dendrobium trantuanii
KY966586.1 Dendrobium trantuanii
JN388583.1 Dendrobium cariniferum
69
KY966524.1 Dendrobium chrysocrepis
100
KY966525.1 Dendrobium chrysocrepis
KF143486.1 Dendrobium menglaense
AB593531.1 Dendrobium chrysocrepis
72
AB593579.1 Dendrobium henryi
KY499231.1 Dendrobium henryi
KJ210456.1 Dendrobium henryi
EF629323.1 Dendrobium henryi
100
KF143470.1 Dendrobium henryi
KJ210455.1 Dendrobium henryi
87
KJ672625.1 Dendrobium henryi
KX600498.1 Dendrobium falconeri
55
KY966539.1 Dendrobium falconeri
JF713102.1 Dendrobium falconeri
66
KY499217.1 Dendrobium falconeri
KJ210447.1 Dendrobium falconeri
100
KF143458.1 Dendrobium falconeri
HQ114239.1 Dendrobium falconeri
91
FJ384734.1 Dendrobium falconeri
KR075038.1 Dendrobium falconeri
66
KJ210446.1 Dendrobium falconeri
AF521610.1 Dendrobium falconeri
100
AF420246.1 Dendrobium falconeri
80
KJ672670.1 Dendrobium falconeri
99
GU339116.1 Dendrobium crystallinum
KY966529.1 Dendrobium crystallinum
KY499225.1 Dendrobium crystallinum
58
KJ210434.1 Dendrobium crystallinum
82
KF143447.1 Dendrobium crystallinum
HQ114243.1 Dendrobium crystallinum
KC205205.1 Dendrobium crystallinum
99
MK169321.1 Dendrobium panduratum
MK169322.1 Dendrobium panduratum
99
86
AB847647.1 Dendrobium cf. bensoniae 036193 B
AB593522.1 Dendrobium cf. gratiosissimum TBG 126713
HM054579.1 Dendrobium bensoniae
94
HM054580.1 Dendrobium bensoniae
87
AB593505.1 Dendrobium bensoniae
KJ672690.1 Dendrobium pendulum
KY966572.1 Dendrobium pendulum
KF143497.1 Dendrobium pendulum
64
KF143496.1 Dendrobium pendulum
JN388596.1 Dendrobium pendulum
HQ114234.1 Dendrobium pendulum
DQ058791.1 Dendrobium pendulum
66
AF362912.1 Dendrobium pendulum
KY499235.1 Dendrobium pendulum
99
KX600502.1 Dendrobium pendulum
KF143498.1 Dendrobium pendulum
GU339115.1 Dendrobium pendulum
EU477508.1 Dendrobium pendulum
KF143465.1Dendrobium gratiosissimum
58
KJ672675.1Dendrobium gratiosissimum
53
KF143464.1Dendrobium gratiosissimum
JN388590.1Dendrobium gratiosissimum
69
AF311780.1Dendrobium gratiosissimum
DQ058790.1Dendrobium gratiosissimum
FJ384737.1Dendrobium gratiosissimum
72
MK522206.1 Dendrobium polyanthum
KX792016.1 Dendrobium salaccense
93
AB593651.1 Dendrobium ruckeri
95
KX600507.1 Dendrobium ruckeri
57
KF143504.1 Dendrobium ruckeri
79
AB593607.1 Dendrobium luteolum
66
AB847672.1 Dendrobium tetrachromum
KY966520.1 Dendrobium chittimae
KF143505.1 Dendrobium ruckeri
KT778761.1 Dendrobium transparens
MK522220.1 Dendrobium transparens
64
MK483278.1 Dendrobium transparens
KX600508.1 Dendrobium transparens
96
AB593679.1 Dendrobium transparens
KF143520.1 Dendrobium transparens

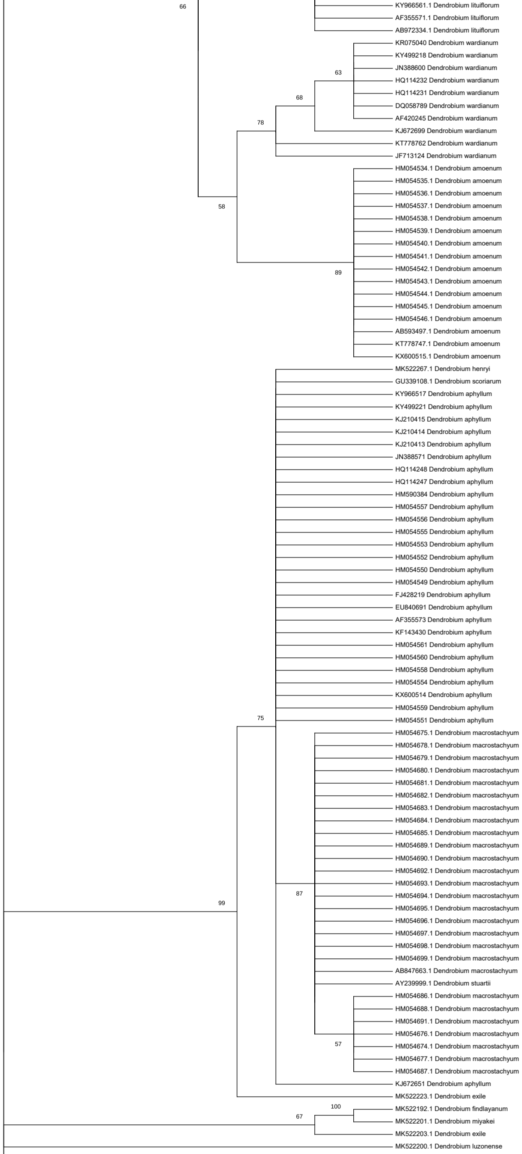
66
KY966561.1 Dendrobium lituiflorum
AF355571.1 Dendrobium lituiflorum
AB972334.1 Dendrobium lituiflorum
KR075040 Dendrobium wardianum
KY499218 Dendrobium wardianum
JN388600 Dendrobium wardianum
63
HQ114232 Dendrobium wardianum
HQ114231 Dendrobium wardianum
68
DQ058789 Dendrobium wardianum
AF420245 Dendrobium wardianum
78
KJ672699 Dendrobium wardianum
KT778762 Dendrobium wardianum
JF713124 Dendrobium wardianum
HM054534.1 Dendrobium amoenum
HM054535.1 Dendrobium amoenum
HM054536.1 Dendrobium amoenum
HM054537.1 Dendrobium amoenum
58
HM054538.1 Dendrobium amoenum
HM054539.1 Dendrobium amoenum
HM054540.1 Dendrobium amoenum
HM054541.1 Dendrobium amoenum
HM054542.1 Dendrobium amoenum
89
HM054543.1 Dendrobium amoenum
HM054544.1 Dendrobium amoenum
HM054545.1 Dendrobium amoenum
HM054546.1 Dendrobium amoenum
AB593497.1 Dendrobium amoenum
KT778747.1 Dendrobium amoenum
KX600515.1 Dendrobium amoenum
MK522267.1 Dendrobium henryi
GU339108.1 Dendrobium scoriarum
KY966517 Dendrobium aphyllum
KY499221 Dendrobium aphyllum
KJ210415 Dendrobium aphyllum
KJ210414 Dendrobium aphyllum
KJ210413 Dendrobium aphyllum
JN388571 Dendrobium aphyllum
HQ114248 Dendrobium aphyllum
HQ114247 Dendrobium aphyllum
HM590384 Dendrobium aphyllum
HM054557 Dendrobium aphyllum
HM054556 Dendrobium aphyllum
HM054555 Dendrobium aphyllum
HM054553 Dendrobium aphyllum
HM054552 Dendrobium aphyllum
HM054550 Dendrobium aphyllum
HM054549 Dendrobium aphyllum
FJ428219 Dendrobium aphyllum
EU840691 Dendrobium aphyllum
AF355573 Dendrobium aphyllum
KF143430 Dendrobium aphyllum
HM054561 Dendrobium aphyllum
HM054560 Dendrobium aphyllum
HM054558 Dendrobium aphyllum
HM054554 Dendrobium aphyllum
KX600514 Dendrobium aphyllum
HM054559 Dendrobium aphyllum
75
HM054551 Dendrobium aphyllum
HM054675.1 Dendrobium macrostachyum
HM054678.1 Dendrobium macrostachyum
HM054679.1 Dendrobium macrostachyum
HM054680.1 Dendrobium macrostachyum
HM054681.1 Dendrobium macrostachyum
HM054682.1 Dendrobium macrostachyum
HM054683.1 Dendrobium macrostachyum
HM054684.1 Dendrobium macrostachyum
HM054685.1 Dendrobium macrostachyum
HM054689.1 Dendrobium macrostachyum
HM054690.1 Dendrobium macrostachyum
HM054692.1 Dendrobium macrostachyum
HM054693.1 Dendrobium macrostachyum
87
HM054694.1 Dendrobium macrostachyum
99
HM054695.1 Dendrobium macrostachyum
HM054696.1 Dendrobium macrostachyum
HM054697.1 Dendrobium macrostachyum
HM054698.1 Dendrobium macrostachyum
HM054699.1 Dendrobium macrostachyum
AB847663.1 Dendrobium macrostachyum
AY239999.1 Dendrobium stuartii
HM054686.1 Dendrobium macrostachyum
HM054688.1 Dendrobium macrostachyum
HM054691.1 Dendrobium macrostachyum
HM054676.1 Dendrobium macrostachyum
57
HM054674.1 Dendrobium macrostachyum
HM054677.1 Dendrobium macrostachyum
HM054687.1 Dendrobium macrostachyum
KJ672651 Dendrobium aphyllum
MK522223.1 Dendrobium exile
100
MK522192.1 Dendrobium findlayanum
67
MK522201.1 Dendrobium miyakei
MK522203.1 Dendrobium exile
MK522200.1 Dendrobium luzonense

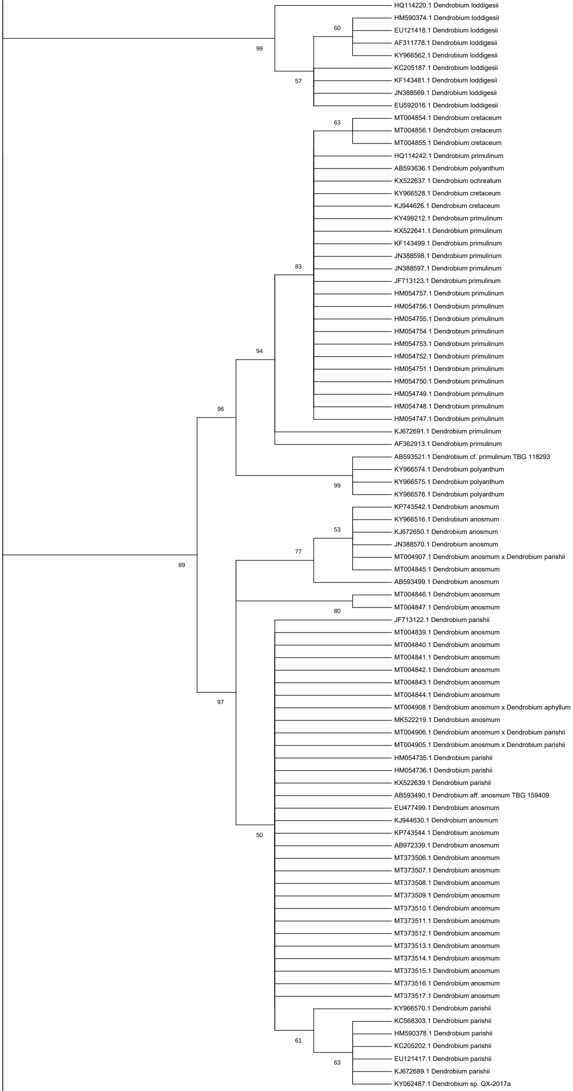

HQ114220.1 Dendrobium loddigesii
HM590374.1 Dendrobium loddigesii
60
EU121418.1 Dendrobium loddigesii
AF311778.1 Dendrobium loddigesii
99
KY966562.1 Dendrobium loddigesii
KC205187.1 Dendrobium loddigesii
57
KF143481.1 Dendrobium loddigesii
JN388569.1 Dendrobium loddigesii
EU592016.1 Dendrobium loddigesii
MT004854.1 Dendrobium cretaceum
63
MT004856.1 Dendrobium cretaceum
MT004855.1 Dendrobium cretaceum
HQ114242.1 Dendrobium primulinum
AB593636.1 Dendrobium polyanthum
KX522637.1 Dendrobium ochreatum
KY966528.1 Dendrobium cretaceum
KJ944626.1 Dendrobium cretaceum
KY499212.1 Dendrobium primulinum
KX522641.1 Dendrobium primulinum
KF143499.1 Dendrobium primulinum
JN388598.1 Dendrobium primulinum
83
JN388597.1 Dendrobium primulinum
JF713123.1 Dendrobium primulinum
HM054757.1 Dendrobium primulinum
HM054756.1 Dendrobium primulinum
HM054755.1 Dendrobium primulinum
HM054754.1 Dendrobium primulinum
HM054753.1 Dendrobium primulinum
94
HM054752.1 Dendrobium primulinum
HM054751.1 Dendrobium primulinum
HM054750.1 Dendrobium primulinum
HM054749.1 Dendrobium primulinum
HM054748.1 Dendrobium primulinum
96
HM054747.1 Dendrobium primulinum
KJ672691.1 Dendrobium primulinum
AF362913.1 Dendrobium primulinum
AB593521.1 Dendrobium cf. primulinum TBG 118293
KY966574.1 Dendrobium polyanthum
KY966575.1 Dendrobium polyanthum
99
KY966576.1 Dendrobium polyanthum
KP743542.1 Dendrobium anosmum
KY966516.1 Dendrobium anosmum
53
KJ672650.1 Dendrobium anosmum
JN388570.1 Dendrobium anosmum
77
MT004907.1 Dendrobium anosmum x Dendrobium parishii
89
MT004845.1 Dendrobium anosmum
AB593499.1 Dendrobium anosmum
MT004846.1 Dendrobium anosmum
MT004847.1 Dendrobium anosmum
80
JF713122.1 Dendrobium parishii
MT004839.1 Dendrobium anosmum
MT004840.1 Dendrobium anosmum
MT004841.1 Dendrobium anosmum
MT004842.1 Dendrobium anosmum
MT004843.1 Dendrobium anosmum
MT004844.1 Dendrobium anosmum
97
MT004908.1 Dendrobium anosmum x Dendrobium aphyllum
MK522219.1 Dendrobium anosmum
MT004906.1 Dendrobium anosmum x Dendrobium parishii
MT004905.1 Dendrobium anosmum x Dendrobium parishii
HM054735.1 Dendrobium parishii
HM054736.1 Dendrobium parishii
KX522639.1 Dendrobium parishii
AB593490.1 Dendrobium aff. anosmum TBG 159409
EU477499.1 Dendrobium anosmum
KJ944630.1 Dendrobium anosmum
50
KP743544.1 Dendrobium anosmum
AB972339.1 Dendrobium anosmum
MT373506.1 Dendrobium anosmum
MT373507.1 Dendrobium anosmum
MT373508.1 Dendrobium anosmum
MT373509.1 Dendrobium anosmum
MT373510.1 Dendrobium anosmum
MT373511.1 Dendrobium anosmum
MT373512.1 Dendrobium anosmum
MT373513.1 Dendrobium anosmum
MT373514.1 Dendrobium anosmum
MT373515.1 Dendrobium anosmum
MT373516.1 Dendrobium anosmum
MT373517.1 Dendrobium anosmum
KY966570.1 Dendrobium parishii
KC568303.1 Dendrobium parishii
HM590378.1 Dendrobium parishii
61
KC205202.1 Dendrobium parishii
EU121417.1 Dendrobium parishii
63
KJ672689.1 Dendrobium parishii
KY062487.1 Dendrobium sp. QX-2017a

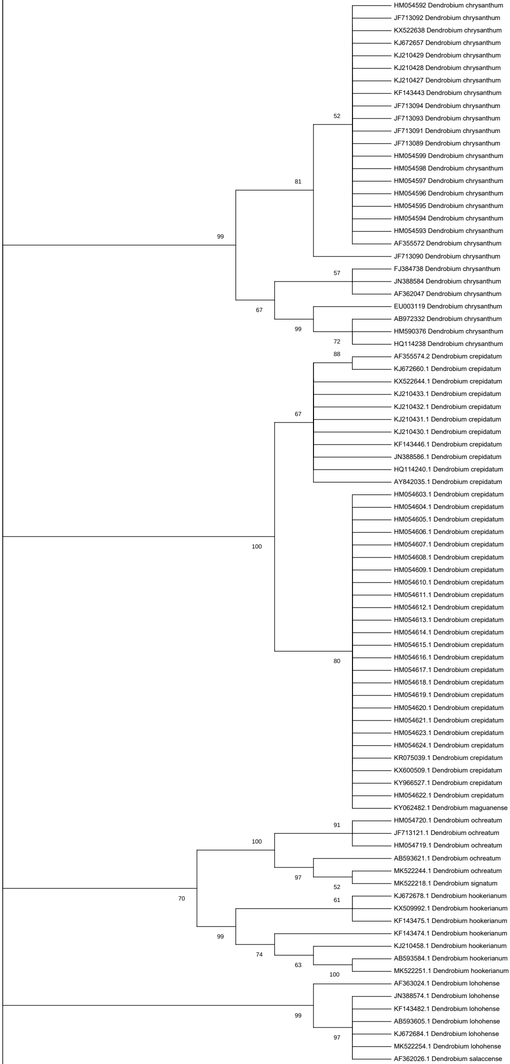
HM054592 Dendrobium chrysanthum
JF713092 Dendrobium chrysanthum
KX522638 Dendrobium chrysanthum
KJ672657 Dendrobium chrysanthum
KJ210429 Dendrobium chrysanthum
KJ210428 Dendrobium chrysanthum
KJ210427 Dendrobium chrysanthum
KF143443 Dendrobium chrysanthum
JF713094 Dendrobium chrysanthum
52
JF713093 Dendrobium chrysanthum
JF713091 Dendrobium chrysanthum
JF713089 Dendrobium chrysanthum
HM054599 Dendrobium chrysanthum
HM054598 Dendrobium chrysanthum
81
HM054597 Dendrobium chrysanthum
HM054596 Dendrobium chrysanthum
HM054595 Dendrobium chrysanthum
HM054594 Dendrobium chrysanthum
HM054593 Dendrobium chrysanthum
99
AF355572 Dendrobium chrysanthum
JF713090 Dendrobium chrysanthum
FJ384738 Dendrobium chrysanthum
57
JN388584 Dendrobium chrysanthum
AF362047 Dendrobium chrysanthum
EU003119 Dendrobium chrysanthum
67
AB972332 Dendrobium chrysanthum
99
HM590376 Dendrobium chrysanthum
72
HQ114238 Dendrobium chrysanthum
88
AF355574.2 Dendrobium crepidatum
KJ672660.1 Dendrobium crepidatum
KX522644.1 Dendrobium crepidatum
KJ210433.1 Dendrobium crepidatum
KJ210432.1 Dendrobium crepidatum
67
KJ210431.1 Dendrobium crepidatum
KJ210430.1 Dendrobium crepidatum
KF143446.1 Dendrobium crepidatum
JN388586.1 Dendrobium crepidatum
HQ114240.1 Dendrobium crepidatum
AY842035.1 Dendrobium crepidatum
HM054603.1 Dendrobium crepidatum
HM054604.1 Dendrobium crepidatum
HM054605.1 Dendrobium crepidatum
HM054606.1 Dendrobium crepidatum
HM054607.1 Dendrobium crepidatum
100
HM054608.1 Dendrobium crepidatum
HM054609.1 Dendrobium crepidatum
HM054610.1 Dendrobium crepidatum
HM054611.1 Dendrobium crepidatum
HM054612.1 Dendrobium crepidatum
HM054613.1 Dendrobium crepidatum
HM054614.1 Dendrobium crepidatum
HM054615.1 Dendrobium crepidatum
HM054616.1 Dendrobium crepidatum
80
HM054617.1 Dendrobium crepidatum
HM054618.1 Dendrobium crepidatum
HM054619.1 Dendrobium crepidatum
HM054620.1 Dendrobium crepidatum
HM054621.1 Dendrobium crepidatum
HM054623.1 Dendrobium crepidatum
HM054624.1 Dendrobium crepidatum
KR075039.1 Dendrobium crepidatum
KX600509.1 Dendrobium crepidatum
KY966527.1 Dendrobium crepidatum
HM054622.1 Dendrobium crepidatum
KY062482.1 Dendrobium maguanense
HM054720.1 Dendrobium ochreatum
91
JF713121.1 Dendrobium ochreatum
100
HM054719.1 Dendrobium ochreatum
AB593621.1 Dendrobium ochreatum
MK522244.1 Dendrobium ochreatum
97
MK522218.1 Dendrobium signatum
52
KJ672678.1 Dendrobium hookerianum
70
61
KX509992.1 Dendrobium hookerianum
KF143475.1 Dendrobium hookerianum
KF143474.1 Dendrobium hookerianum
99
KJ210458.1 Dendrobium hookerianum
74
AB593584.1 Dendrobium hookerianum
63
MK522251.1 Dendrobium hookerianum
100
AF363024.1 Dendrobium lohohense
JN388574.1 Dendrobium lohohense
KF143482.1 Dendrobium lohohense
99
AB593605.1 Dendrobium lohohense
KJ672684.1 Dendrobium lohohense
97
MK522254.1 Dendrobium lohohense
AF362026.1 Dendrobium salaccense

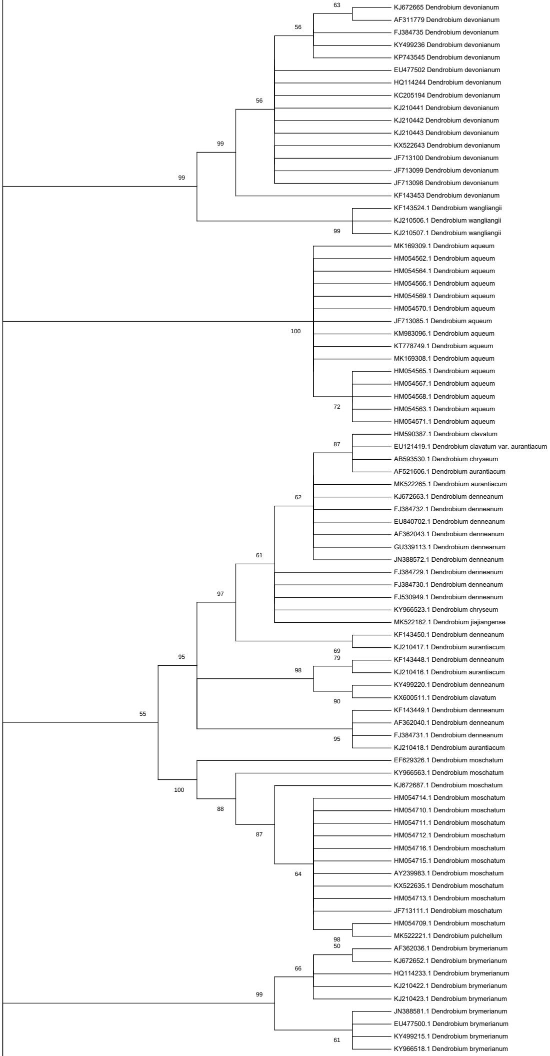
63
KJ672665 Dendrobium devonianum
AF311779 Dendrobium devonianum
56
FJ384735 Dendrobium devonianum
KY499236 Dendrobium devonianum
KP743545 Dendrobium devonianum
EU477502 Dendrobium devonianum
HQ114244 Dendrobium devonianum
KC205194 Dendrobium devonianum
56
KJ210441 Dendrobium devonianum
KJ210442 Dendrobium devonianum
KJ210443 Dendrobium devonianum
99
KX522643 Dendrobium devonianum
JF713100 Dendrobium devonianum
JF713099 Dendrobium devonianum
99
JF713098 Dendrobium devonianum
KF143453 Dendrobium devonianum
KF143524.1 Dendrobium wangliangii
KJ210506.1 Dendrobium wangliangii
99
KJ210507.1 Dendrobium wangliangii
MK169309.1 Dendrobium aqueum
HM054562.1 Dendrobium aqueum
HM054564.1 Dendrobium aqueum
HM054566.1 Dendrobium aqueum
HM054569.1 Dendrobium aqueum
HM054570.1 Dendrobium aqueum
JF713085.1 Dendrobium aqueum
100
KM983096.1 Dendrobium aqueum
KT778749.1 Dendrobium aqueum
MK169308.1 Dendrobium aqueum
HM054565.1 Dendrobium aqueum
HM054567.1 Dendrobium aqueum
HM054568.1 Dendrobium aqueum
72
HM054563.1 Dendrobium aqueum
HM054571.1 Dendrobium aqueum
HM590387.1 Dendrobium clavatum
87
EU121419.1 Dendrobium clavatum var. aurantiacum
AB593530.1 Dendrobium chryseum
AF521606.1 Dendrobium aurantiacum
MK522265.1 Dendrobium aurantiacum
62
KJ672663.1 Dendrobium denneanum
FJ384732.1 Dendrobium denneanum
EU840702.1 Dendrobium denneanum
AF362043.1 Dendrobium denneanum
GU339113.1 Dendrobium denneanum
61
JN388572.1 Dendrobium denneanum
FJ384729.1 Dendrobium denneanum
FJ384730.1 Dendrobium denneanum
97
FJ530949.1 Dendrobium denneanum
KY966523.1 Dendrobium chryseum
MK522182.1 Dendrobium jiajiangense
KF143450.1 Dendrobium denneanum
KJ210417.1 Dendrobium aurantiacum
69
79
95
KF143448.1 Dendrobium denneanum
98
KJ210416.1 Dendrobium aurantiacum
KY499220.1 Dendrobium denneanum
KX600511.1 Dendrobium clavatum
90
KF143449.1 Dendrobium denneanum
55
AF362040.1 Dendrobium denneanum
95
FJ384731.1 Dendrobium denneanum
KJ210418.1 Dendrobium aurantiacum
EF629326.1 Dendrobium moschatum
KY966563.1 Dendrobium moschatum
KJ672687.1 Dendrobium moschatum
100
HM054714.1 Dendrobium moschatum
88
HM054710.1 Dendrobium moschatum
HM054711.1 Dendrobium moschatum
87
HM054712.1 Dendrobium moschatum
HM054716.1 Dendrobium moschatum
HM054715.1 Dendrobium moschatum
64
AY239983.1 Dendrobium moschatum
KX522635.1 Dendrobium moschatum
HM054713.1 Dendrobium moschatum
JF713111.1 Dendrobium moschatum
HM054709.1 Dendrobium moschatum
MK522221.1 Dendrobium pulchellum
98
50
AF362036.1 Dendrobium brymerianum
KJ672652.1 Dendrobium brymerianum
66
HQ114233.1 Dendrobium brymerianum
99
KJ210422.1 Dendrobium brymerianum
KJ210423.1 Dendrobium brymerianum
JN388581.1 Dendrobium brymerianum
EU477500.1 Dendrobium brymerianum
KY499215.1 Dendrobium brymerianum
61
KY966518.1 Dendrobium brymerianum

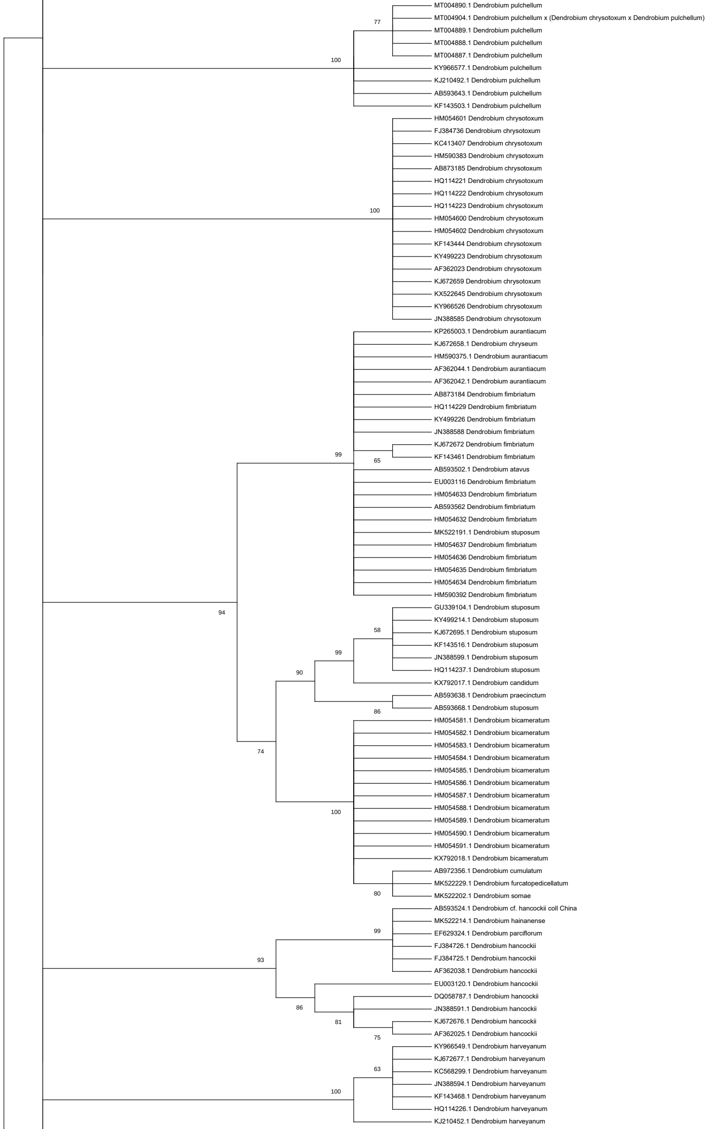
MT004890.1 Dendrobium pulchellum
MT004904.1 Dendrobium pulchellum x (Dendrobium chrysotoxum x Dendrobium pulchellum)
77
MT004889.1 Dendrobium pulchellum
MT004888.1 Dendrobium pulchellum
MT004887.1 Dendrobium pulchellum
100
KY966577.1 Dendrobium pulchellum
KJ210492.1 Dendrobium pulchellum
AB593643.1 Dendrobium pulchellum
KF143503.1 Dendrobium pulchellum
HM054601 Dendrobium chrysotoxum
FJ384736 Dendrobium chrysotoxum
KC413407 Dendrobium chrysotoxum
HM590383 Dendrobium chrysotoxum
AB873185 Dendrobium chrysotoxum
HQ114221 Dendrobium chrysotoxum
HQ114222 Dendrobium chrysotoxum
100
HQ114223 Dendrobium chrysotoxum
HM054600 Dendrobium chrysotoxum
HM054602 Dendrobium chrysotoxum
KF143444 Dendrobium chrysotoxum
KY499223 Dendrobium chrysotoxum
AF362023 Dendrobium chrysotoxum
KJ672659 Dendrobium chrysotoxum
KX522645 Dendrobium chrysotoxum
KY966526 Dendrobium chrysotoxum
JN388585 Dendrobium chrysotoxum
KP265003.1 Dendrobium aurantiacum
KJ672658.1 Dendrobium chryseum
HM590375.1 Dendrobium aurantiacum
AF362044.1 Dendrobium aurantiacum
AF362042.1 Dendrobium aurantiacum
AB873184 Dendrobium fimbriatum
HQ114229 Dendrobium fimbriatum
KY499226 Dendrobium fimbriatum
JN388588 Dendrobium fimbriatum
KJ672672 Dendrobium fimbriatum
99
KF143461 Dendrobium fimbriatum
65
AB593502.1 Dendrobium atavus
EU003116 Dendrobium fimbriatum
HM054633 Dendrobium fimbriatum
AB593562 Dendrobium fimbriatum
HM054632 Dendrobium fimbriatum
MK522191.1 Dendrobium stuposum
HM054637 Dendrobium fimbriatum
HM054636 Dendrobium fimbriatum
HM054635 Dendrobium fimbriatum
HM054634 Dendrobium fimbriatum
HM590392 Dendrobium fimbriatum
GU339104.1 Dendrobium stuposum
94
KY499214.1 Dendrobium stuposum
KJ672695.1 Dendrobium stuposum
58
KF143516.1 Dendrobium stuposum
99
JN388599.1 Dendrobium stuposum
HQ114237.1 Dendrobium stuposum
90
KX792017.1 Dendrobium candidum
AB593638.1 Dendrobium praecinctum
AB593668.1 Dendrobium stuposum
86
HM054581.1 Dendrobium bicameratum
HM054582.1 Dendrobium bicameratum
HM054583.1 Dendrobium bicameratum
74
HM054584.1 Dendrobium bicameratum
HM054585.1 Dendrobium bicameratum
HM054586.1 Dendrobium bicameratum
HM054587.1 Dendrobium bicameratum
HM054588.1 Dendrobium bicameratum
100
HM054589.1 Dendrobium bicameratum
HM054590.1 Dendrobium bicameratum
HM054591.1 Dendrobium bicameratum
KX792018.1 Dendrobium bicameratum
AB972356.1 Dendrobium cumulatum
MK522229.1 Dendrobium furcatopedicellatum
80
MK522202.1 Dendrobium somae
AB593524.1 Dendrobium cf. hancockii coll China
MK522214.1 Dendrobium hainanense
99
EF629324.1 Dendrobium parciflorum
FJ384726.1 Dendrobium hancockii
93
FJ384725.1 Dendrobium hancockii
AF362038.1 Dendrobium hancockii
EU003120.1 Dendrobium hancockii
DQ058787.1 Dendrobium hancockii
86
JN388591.1 Dendrobium hancockii
81
KJ672676.1 Dendrobium hancockii
75
AF362025.1 Dendrobium hancockii
KY966549.1 Dendrobium harveyanum
KJ672677.1 Dendrobium harveyanum
63
KC568299.1 Dendrobium harveyanum
JN388594.1 Dendrobium harveyanum
100
KF143468.1 Dendrobium harveyanum
HQ114226.1 Dendrobium harveyanum
KJ210452.1 Dendrobium harveyanum

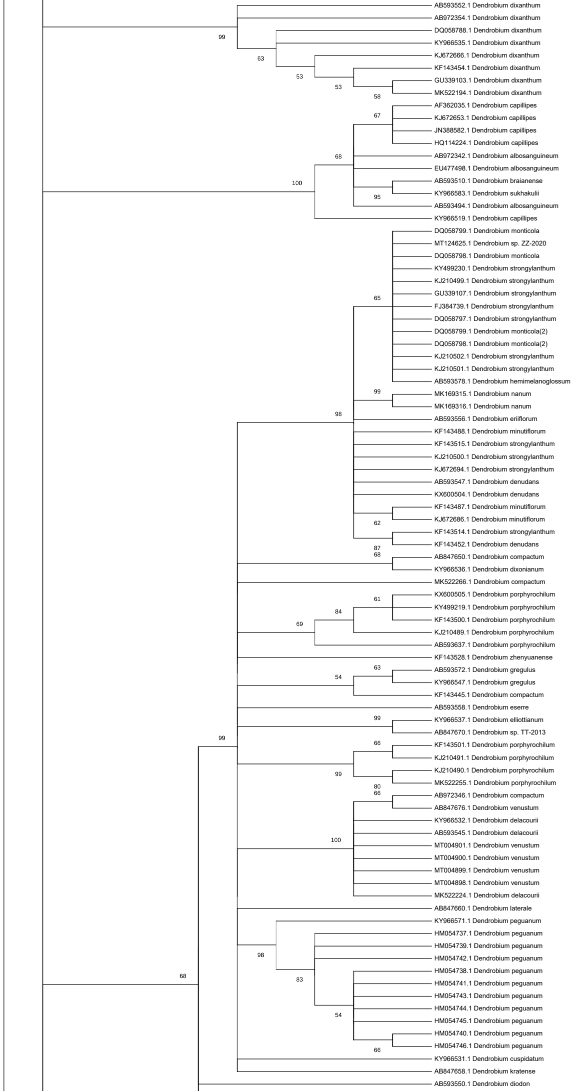

AB593552.1 Dendrobium dixanthum
AB972354.1 Dendrobium dixanthum
99
DQ058788.1 Dendrobium dixanthum
KY966535.1 Dendrobium dixanthum
63
KJ672666.1 Dendrobium dixanthum
KF143454.1 Dendrobium dixanthum
53
53
GU339103.1 Dendrobium dixanthum
MK522194.1 Dendrobium dixanthum
58
AF362035.1 Dendrobium capillipes
67
KJ672653.1 Dendrobium capillipes
JN388582.1 Dendrobium capillipes
HQ114224.1 Dendrobium capillipes
AB972342.1 Dendrobium albosanguineum
68
EU477498.1 Dendrobium albosanguineum
AB593510.1 Dendrobium braianense
100
KY966583.1 Dendrobium sukhakulii
95
AB593494.1 Dendrobium albosanguineum
KY966519.1 Dendrobium capillipes
DQ058799.1 Dendrobium monticola
MT124625.1 Dendrobium sp. ZZ-2020
DQ058798.1 Dendrobium monticola
KY499230.1 Dendrobium strongylanthum
KJ210499.1 Dendrobium strongylanthum
GU339107.1 Dendrobium strongylanthum
65
FJ384739.1 Dendrobium strongylanthum
DQ058797.1 Dendrobium strongylanthum
DQ058799.1 Dendrobium monticola(2)
DQ058798.1 Dendrobium monticola(2)
KJ210502.1 Dendrobium strongylanthum
KJ210501.1 Dendrobium strongylanthum
AB593578.1 Dendrobium hemimelanoglossum
99
MK169315.1 Dendrobium nanum
MK169316.1 Dendrobium nanum
98
AB593556.1 Dendrobium eriiflorum
KF143488.1 Dendrobium minutiflorum
KF143515.1 Dendrobium strongylanthum
KJ210500.1 Dendrobium strongylanthum
KJ672694.1 Dendrobium strongylanthum
AB593547.1 Dendrobium denudans
KX600504.1 Dendrobium denudans
KF143487.1 Dendrobium minutiflorum
KJ672686.1 Dendrobium minutiflorum
62
KF143514.1 Dendrobium strongylanthum
KF143452.1 Dendrobium denudans
87
68
AB847650.1 Dendrobium compactum
KY966536.1 Dendrobium dixonianum
MK522266.1 Dendrobium compactum
KX600505.1 Dendrobium porphyrochilum
61
KY499219.1 Dendrobium porphyrochilum
84
KF143500.1 Dendrobium porphyrochilum
69
KJ210489.1 Dendrobium porphyrochilum
AB593637.1 Dendrobium porphyrochilum
KF143528.1 Dendrobium zhenyuanense
63
AB593572.1 Dendrobium gregulus
54
KY966547.1 Dendrobium gregulus
KF143445.1 Dendrobium compactum
AB593558.1 Dendrobium eserre
99
KY966537.1 Dendrobium elliottianum
AB847670.1 Dendrobium sp. TT-2013
99
66
KF143501.1 Dendrobium porphyrochilum
KJ210491.1 Dendrobium porphyrochilum
99
KJ210490.1 Dendrobium porphyrochilum
MK522255.1 Dendrobium porphyrochilum
80
66
AB972346.1 Dendrobium compactum
AB847676.1 Dendrobium venustum
KY966532.1 Dendrobium delacourii
AB593545.1 Dendrobium delacourii
100
MT004901.1 Dendrobium venustum
MT004900.1 Dendrobium venustum
MT004899.1 Dendrobium venustum
MT004898.1 Dendrobium venustum
MK522224.1 Dendrobium delacourii
AB847660.1 Dendrobium laterale
KY966571.1 Dendrobium peguanum
HM054737.1 Dendrobium peguanum
HM054739.1 Dendrobium peguanum
98
HM054742.1 Dendrobium peguanum
HM054738.1 Dendrobium peguanum
68
83
HM054741.1 Dendrobium peguanum
HM054743.1 Dendrobium peguanum
HM054744.1 Dendrobium peguanum
54
HM054745.1 Dendrobium peguanum
HM054740.1 Dendrobium peguanum
HM054746.1 Dendrobium peguanum
66
KY966531.1 Dendrobium cuspidatum
AB847658.1 Dendrobium kratense
AB593550.1 Dendrobium diodon

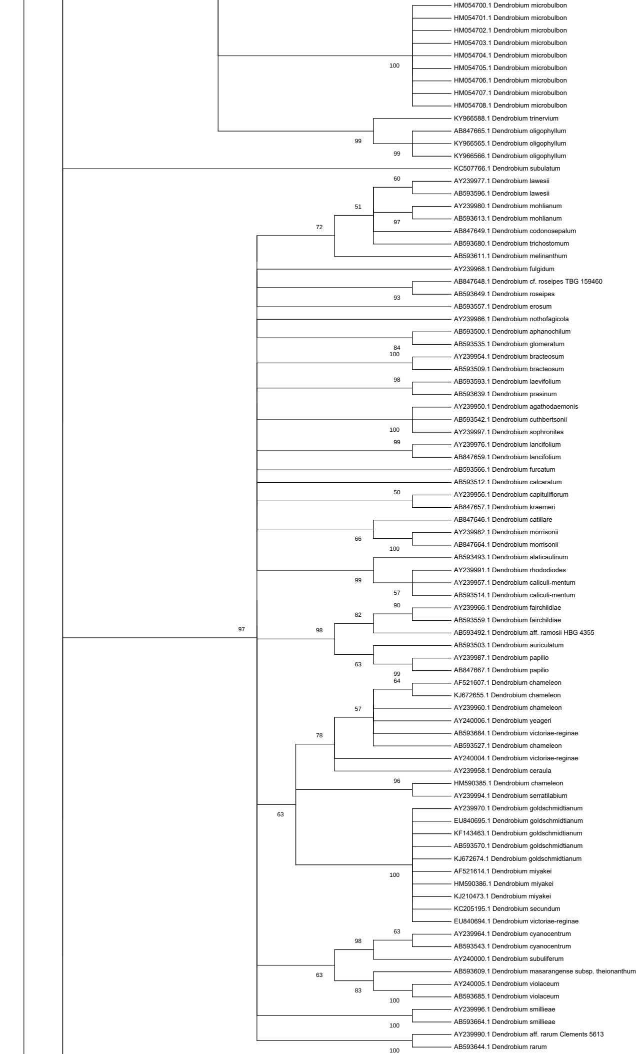
HM054700.1 Dendrobium microbulbon
HM054701.1 Dendrobium microbulbon
HM054702.1 Dendrobium microbulbon
HM054703.1 Dendrobium microbulbon
HM054704.1 Dendrobium microbulbon
100
HM054705.1 Dendrobium microbulbon
HM054706.1 Dendrobium microbulbon
HM054707.1 Dendrobium microbulbon
HM054708.1 Dendrobium microbulbon
KY966588.1 Dendrobium trinervium
AB847665.1 Dendrobium oligophyllum
99
KY966565.1 Dendrobium oligophyllum
99
KY966566.1 Dendrobium oligophyllum
KC507766.1 Dendrobium subulatum
60
AY239977.1 Dendrobium lawesii
AB593596.1 Dendrobium lawesii
51
AY239980.1 Dendrobium mohlianum
AB593613.1 Dendrobium mohlianum
97
72
AB847649.1 Dendrobium codonosepalum
AB593680.1 Dendrobium trichostomum
AB593611.1 Dendrobium melinanthum
AY239968.1 Dendrobium fulgidum
AB847648.1 Dendrobium cf. roseipes TBG 159460
AB593649.1 Dendrobium roseipes
93
AB593557.1 Dendrobium erosum
AY239986.1 Dendrobium nothofagicola
AB593500.1 Dendrobium aphanochilum
AB593535.1 Dendrobium glomeratum
84
100
AY239954.1 Dendrobium bracteosum
AB593509.1 Dendrobium bracteosum
98
AB593593.1 Dendrobium laevifolium
AB593639.1 Dendrobium prasinum
AY239950.1 Dendrobium agathodaemonis
AB593542.1 Dendrobium cuthbertsonii
100
AY239997.1 Dendrobium sophronites
99
AY239976.1 Dendrobium lancifolium
AB847659.1 Dendrobium lancifolium
AB593566.1 Dendrobium furcatum
AB593512.1 Dendrobium calcaratum
50
AY239956.1 Dendrobium capituliflorum
AB847657.1 Dendrobium kraemeri
AB847646.1 Dendrobium catillare
AY239982.1 Dendrobium morrisonii
66
AB847664.1 Dendrobium morrisonii
100
AB593493.1 Dendrobium alaticaulinum
AY239991.1 Dendrobium rhododiodes
99
AY239957.1 Dendrobium caliculi-mentum
57
AB593514.1 Dendrobium caliculi-mentum
90
AY239966.1 Dendrobium fairchildiae
82
AB593559.1 Dendrobium fairchildiae
97
98
AB593492.1 Dendrobium aff. ramosii HBG 4355
AB593503.1 Dendrobium auriculatum
AY239987.1 Dendrobium papilio
63
AB847667.1 Dendrobium papilio
99
64
AF521607.1 Dendrobium chameleon
KJ672655.1 Dendrobium chameleon
57
AY239960.1 Dendrobium chameleon
AY240006.1 Dendrobium yeageri
78
AB593684.1 Dendrobium victoriae-reginae
AB593527.1 Dendrobium chameleon
AY240004.1 Dendrobium victoriae-reginae
AY239958.1 Dendrobium ceraula
96
HM590385.1 Dendrobium chameleon
AY239994.1 Dendrobium serratilabium
AY239970.1 Dendrobium goldschmidtianum
63
EU840695.1 Dendrobium goldschmidtianum
KF143463.1 Dendrobium goldschmidtianum
AB593570.1 Dendrobium goldschmidtianum
KJ672674.1 Dendrobium goldschmidtianum
AF521614.1 Dendrobium miyakei
100
HM590386.1 Dendrobium miyakei
KJ210473.1 Dendrobium miyakei
KC205195.1 Dendrobium secundum
EU840694.1 Dendrobium victoriae-reginae
63
AY239964.1 Dendrobium cyanocentrum
98
AB593543.1 Dendrobium cyanocentrum
AY240000.1 Dendrobium subuliferum
63
AB593609.1 Dendrobium masarangense subsp. theionanthum
AY240005.1 Dendrobium violaceum
83
AB593685.1 Dendrobium violaceum
100
AY239996.1 Dendrobium smillieae
AB593664.1 Dendrobium smillieae
100
AY239990.1 Dendrobium aff. rarum Clements 5613
AB593644.1 Dendrobium rarum
100

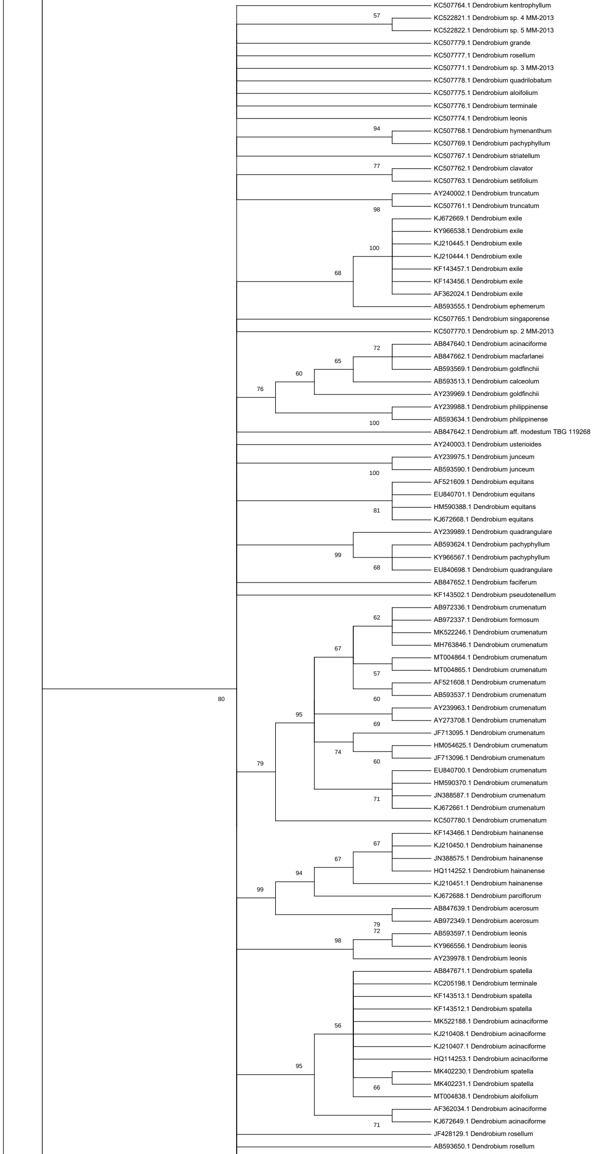
KC507764.1 Dendrobium kentrophyllum
57
KC522821.1 Dendrobium sp. 4 MM-2013
KC522822.1 Dendrobium sp. 5 MM-2013
KC507779.1 Dendrobium grande
KC507777.1 Dendrobium rosellum
KC507771.1 Dendrobium sp. 3 MM-2013
KC507778.1 Dendrobium quadrilobatum
KC507775.1 Dendrobium aloifolium
KC507776.1 Dendrobium terminale
KC507774.1 Dendrobium leonis
94
KC507768.1 Dendrobium hymenanthum
KC507769.1 Dendrobium pachyphyllum
KC507767.1 Dendrobium striatellum
77
KC507762.1 Dendrobium clavator
KC507763.1 Dendrobium setifolium
AY240002.1 Dendrobium truncatum
KC507761.1 Dendrobium truncatum
98
KJ672669.1 Dendrobium exile
KY966538.1 Dendrobium exile
KJ210445.1 Dendrobium exile
100
KJ210444.1 Dendrobium exile
68
KF143457.1 Dendrobium exile
KF143456.1 Dendrobium exile
AF362024.1 Dendrobium exile
AB593555.1 Dendrobium ephemerum
KC507765.1 Dendrobium singaporense
KC507770.1 Dendrobium sp. 2 MM-2013
AB847640.1 Dendrobium acinaciforme
72
AB847662.1 Dendrobium macfarlanei
65
AB593569.1 Dendrobium goldfinchii
60
AB593513.1 Dendrobium calceolum
76
AY239969.1 Dendrobium goldfinchii
AY239988.1 Dendrobium philippinense
AB593634.1 Dendrobium philippinense
100
AB847642.1 Dendrobium aff. modestum TBG 119268
AY240003.1 Dendrobium usterioides
AY239975.1 Dendrobium junceum
AB593590.1 Dendrobium junceum
100
AF521609.1 Dendrobium equitans
EU840701.1 Dendrobium equitans
HM590388.1 Dendrobium equitans
81
KJ672668.1 Dendrobium equitans
AY239989.1 Dendrobium quadrangulare
AB593624.1 Dendrobium pachyphyllum
99
KY966567.1 Dendrobium pachyphyllum
68
EU840698.1 Dendrobium quadrangulare
AB847652.1 Dendrobium faciferum
KF143502.1 Dendrobium pseudotenellum
AB972336.1 Dendrobium crumenatum
62
AB972337.1 Dendrobium formosum
MK522246.1 Dendrobium crumenatum
MH763846.1 Dendrobium crumenatum
67
MT004864.1 Dendrobium crumenatum
MT004865.1 Dendrobium crumenatum
57
AF521608.1 Dendrobium crumenatum
80
AB593537.1 Dendrobium crumenatum
60
95
AY239963.1 Dendrobium crumenatum
AY273708.1 Dendrobium crumenatum
69
JF713095.1 Dendrobium crumenatum
79
HM054625.1 Dendrobium crumenatum
74
JF713096.1 Dendrobium crumenatum
60
EU840700.1 Dendrobium crumenatum
HM590370.1 Dendrobium crumenatum
JN388587.1 Dendrobium crumenatum
71
KJ672661.1 Dendrobium crumenatum
KC507780.1 Dendrobium crumenatum
KF143466.1 Dendrobium hainanense
67
KJ210450.1 Dendrobium hainanense
67
JN388575.1 Dendrobium hainanense
94
HQ114252.1 Dendrobium hainanense
99
KJ210451.1 Dendrobium hainanense
KJ672688.1 Dendrobium parciflorum
AB847639.1 Dendrobium acerosum
AB972349.1 Dendrobium acerosum
79
72
AB593597.1 Dendrobium leonis
98
KY966556.1 Dendrobium leonis
AY239978.1 Dendrobium leonis
AB847671.1 Dendrobium spatella
KC205198.1 Dendrobium terminale
KF143513.1 Dendrobium spatella
KF143512.1 Dendrobium spatella
56
MK522188.1 Dendrobium acinaciforme
KJ210408.1 Dendrobium acinaciforme
KJ210407.1 Dendrobium acinaciforme
95
HQ114253.1 Dendrobium acinaciforme
MK402230.1 Dendrobium spatella
66
MK402231.1 Dendrobium spatella
MT004838.1 Dendrobium aloifolium
AF362034.1 Dendrobium acinaciforme
KJ672649.1 Dendrobium acinaciforme
71
JF428129.1 Dendrobium rosellum
AB593650.1 Dendrobium rosellum

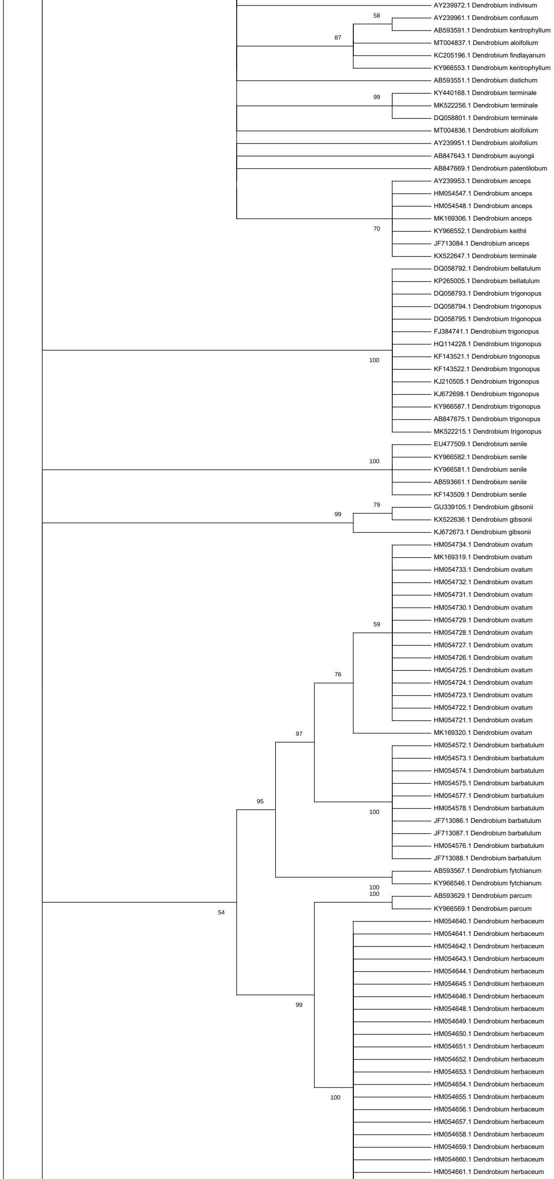
AY239972.1 Dendrobium indivisum
AY239961.1 Dendrobium confusum
AB593591.1 Dendrobium kentrophyllum
MT004837.1 Dendrobium aloifolium
KC205196.1 Dendrobium findlayanum
KY966553.1 Dendrobium kentrophyllum
AB593551.1 Dendrobium distichum
KY440168.1 Dendrobium terminale
MK522256.1 Dendrobium terminale
DQ058801.1 Dendrobium terminale
MT004836.1 Dendrobium aloifolium
AY239951.1 Dendrobium aloifolium
AB847643.1 Dendrobium auyongii
AB847669.1 Dendrobium patentilobum
AY239953.1 Dendrobium anceps
HM054547.1 Dendrobium anceps
HM054548.1 Dendrobium anceps
MK169306.1 Dendrobium anceps
KY966552.1 Dendrobium keithii
JF713084.1 Dendrobium anceps
KX522647.1 Dendrobium terminale
DQ058792.1 Dendrobium bellatulum
KP265005.1 Dendrobium bellatulum
DQ058793.1 Dendrobium trigonopus
DQ058794.1 Dendrobium trigonopus
DQ058795.1 Dendrobium trigonopus
FJ384741.1 Dendrobium trigonopus
HQ114228.1 Dendrobium trigonopus
KF143521.1 Dendrobium trigonopus
KF143522.1 Dendrobium trigonopus
KJ210505.1 Dendrobium trigonopus
KJ672698.1 Dendrobium trigonopus
KY966587.1 Dendrobium trigonopus
AB847675.1 Dendrobium trigonopus
MK522215.1 Dendrobium trigonopus
EU477509.1 Dendrobium senile
KY966582.1 Dendrobium senile
KY966581.1 Dendrobium senile
AB593661.1 Dendrobium senile
KF143509.1 Dendrobium senile
GU339105.1 Dendrobium gibsonii
KX522636.1 Dendrobium gibsonii
KJ672673.1 Dendrobium gibsonii
HM054734.1 Dendrobium ovatum
MK169319.1 Dendrobium ovatum
HM054733.1 Dendrobium ovatum
HM054732.1 Dendrobium ovatum
HM054731.1 Dendrobium ovatum
HM054730.1 Dendrobium ovatum
HM054729.1 Dendrobium ovatum
HM054728.1 Dendrobium ovatum
HM054727.1 Dendrobium ovatum
HM054726.1 Dendrobium ovatum
HM054725.1 Dendrobium ovatum
HM054724.1 Dendrobium ovatum
HM054723.1 Dendrobium ovatum
HM054722.1 Dendrobium ovatum
HM054721.1 Dendrobium ovatum
MK169320.1 Dendrobium ovatum
HM054572.1 Dendrobium barbatulum
HM054573.1 Dendrobium barbatulum
HM054574.1 Dendrobium barbatulum
HM054575.1 Dendrobium barbatulum
HM054577.1 Dendrobium barbatulum
HM054578.1 Dendrobium barbatulum
JF713086.1 Dendrobium barbatulum
JF713087.1 Dendrobium barbatulum
HM054576.1 Dendrobium barbatulum
JF713088.1 Dendrobium barbatulum
AB593567.1 Dendrobium fytchianum
KY966546.1 Dendrobium fytchianum
AB593629.1 Dendrobium parcum
KY966569.1 Dendrobium parcum
HM054640.1 Dendrobium herbaceum
HM054641.1 Dendrobium herbaceum
HM054642.1 Dendrobium herbaceum
HM054643.1 Dendrobium herbaceum
HM054644.1 Dendrobium herbaceum
HM054645.1 Dendrobium herbaceum
HM054646.1 Dendrobium herbaceum
HM054648.1 Dendrobium herbaceum
HM054649.1 Dendrobium herbaceum
HM054650.1 Dendrobium herbaceum
HM054651.1 Dendrobium herbaceum
HM054652.1 Dendrobium herbaceum
HM054653.1 Dendrobium herbaceum
HM054654.1 Dendrobium herbaceum
HM054655.1 Dendrobium herbaceum
HM054656.1 Dendrobium herbaceum
HM054657.1 Dendrobium herbaceum
HM054658.1 Dendrobium herbaceum
HM054659.1 Dendrobium herbaceum
HM054660.1 Dendrobium herbaceum
HM054661.1 Dendrobium herbaceum

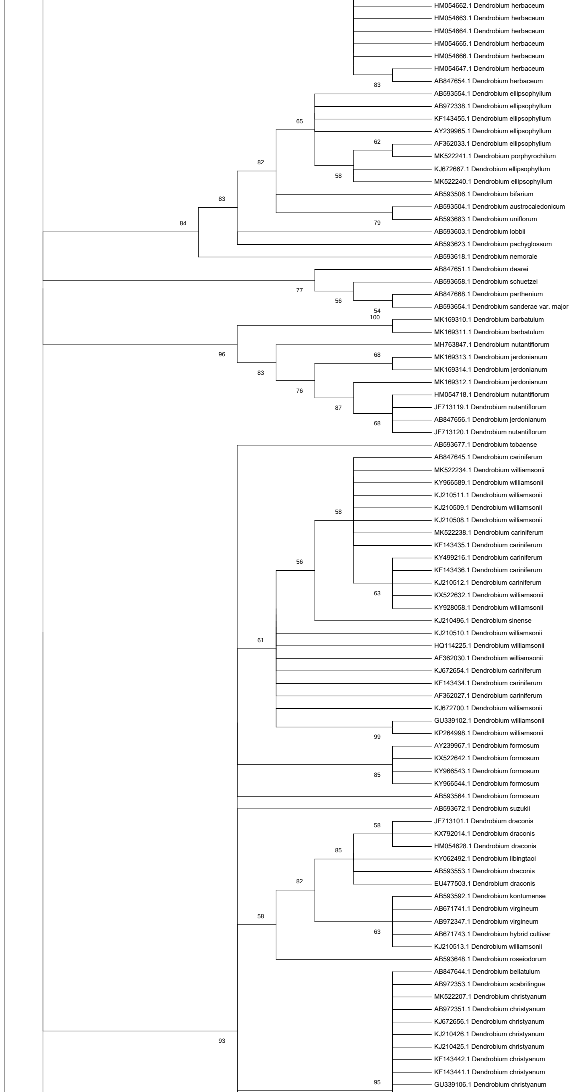
HM054662.1 Dendrobium herbaceum
HM054663.1 Dendrobium herbaceum
HM054664.1 Dendrobium herbaceum
HM054665.1 Dendrobium herbaceum
HM054666.1 Dendrobium herbaceum
HM054647.1 Dendrobium herbaceum
AB847654.1 Dendrobium herbaceum
83
AB593554.1 Dendrobium ellipsophyllum
AB972338.1 Dendrobium ellipsophyllum
65
KF143455.1 Dendrobium ellipsophyllum
AY239965.1 Dendrobium ellipsophyllum
62
AF362033.1 Dendrobium ellipsophyllum
MK522241.1 Dendrobium porphyrochilum
82
KJ672667.1 Dendrobium ellipsophyllum
58
MK522240.1 Dendrobium ellipsophyllum
AB593506.1 Dendrobium bifarium
83
AB593504.1 Dendrobium austrocaledonicum
84
AB593683.1 Dendrobium uniflorum
79
AB593603.1 Dendrobium lobbii
AB593623.1 Dendrobium pachyglossum
AB593618.1 Dendrobium nemorale
AB847651.1 Dendrobium dearei
AB593658.1 Dendrobium schuetzei
77
AB847668.1 Dendrobium parthenium
56
AB593654.1 Dendrobium sanderae var. major
54
100
MK169310.1 Dendrobium barbatulum
MK169311.1 Dendrobium barbatulum
MH763847.1 Dendrobium nutantiflorum
96
68
MK169313.1 Dendrobium jerdonianum
83
MK169314.1 Dendrobium jerdonianum
MK169312.1 Dendrobium jerdonianum
76
HM054718.1 Dendrobium nutantiflorum
87
JF713119.1 Dendrobium nutantiflorum
AB847656.1 Dendrobium jerdonianum
68
JF713120.1 Dendrobium nutantiflorum
AB593677.1 Dendrobium tobaense
AB847645.1 Dendrobium cariniferum
MK522234.1 Dendrobium williamsonii
KY966589.1 Dendrobium williamsonii
KJ210511.1 Dendrobium williamsonii
KJ210509.1 Dendrobium williamsonii
58
KJ210508.1 Dendrobium williamsonii
MK522238.1 Dendrobium cariniferum
KF143435.1 Dendrobium cariniferum
KY499216.1 Dendrobium cariniferum
56
KF143436.1 Dendrobium cariniferum
KJ210512.1 Dendrobium cariniferum
63
KX522632.1 Dendrobium williamsonii
KY928058.1 Dendrobium williamsonii
KJ210496.1 Dendrobium sinense
KJ210510.1 Dendrobium williamsonii
61
HQ114225.1 Dendrobium williamsonii
AF362030.1 Dendrobium williamsonii
KJ672654.1 Dendrobium cariniferum
KF143434.1 Dendrobium cariniferum
AF362027.1 Dendrobium cariniferum
KJ672700.1 Dendrobium williamsonii
GU339102.1 Dendrobium williamsonii
KP264998.1 Dendrobium williamsonii
99
AY239967.1 Dendrobium formosum
KX522642.1 Dendrobium formosum
KY966543.1 Dendrobium formosum
85
KY966544.1 Dendrobium formosum
AB593564.1 Dendrobium formosum
AB593672.1 Dendrobium suzukii
JF713101.1 Dendrobium draconis
58
KX792014.1 Dendrobium draconis
HM054628.1 Dendrobium draconis
85
KY062492.1 Dendrobium libingtaoi
AB593553.1 Dendrobium draconis
82
EU477503.1 Dendrobium draconis
AB593592.1 Dendrobium kontumense
AB671741.1 Dendrobium virgineum
58
AB972347.1 Dendrobium virgineum
AB671743.1 Dendrobium hybrid cultivar
63
KJ210513.1 Dendrobium williamsonii
AB593648.1 Dendrobium roseiodorum
AB847644.1 Dendrobium bellatulum
AB972353.1 Dendrobium scabrilingue
MK522207.1 Dendrobium christyanum
AB972351.1 Dendrobium christyanum
KJ672656.1 Dendrobium christyanum
KJ210426.1 Dendrobium christyanum
93
KJ210425.1 Dendrobium christyanum
KF143442.1 Dendrobium christyanum
KF143441.1 Dendrobium christyanum
95
GU339106.1 Dendrobium christyanum

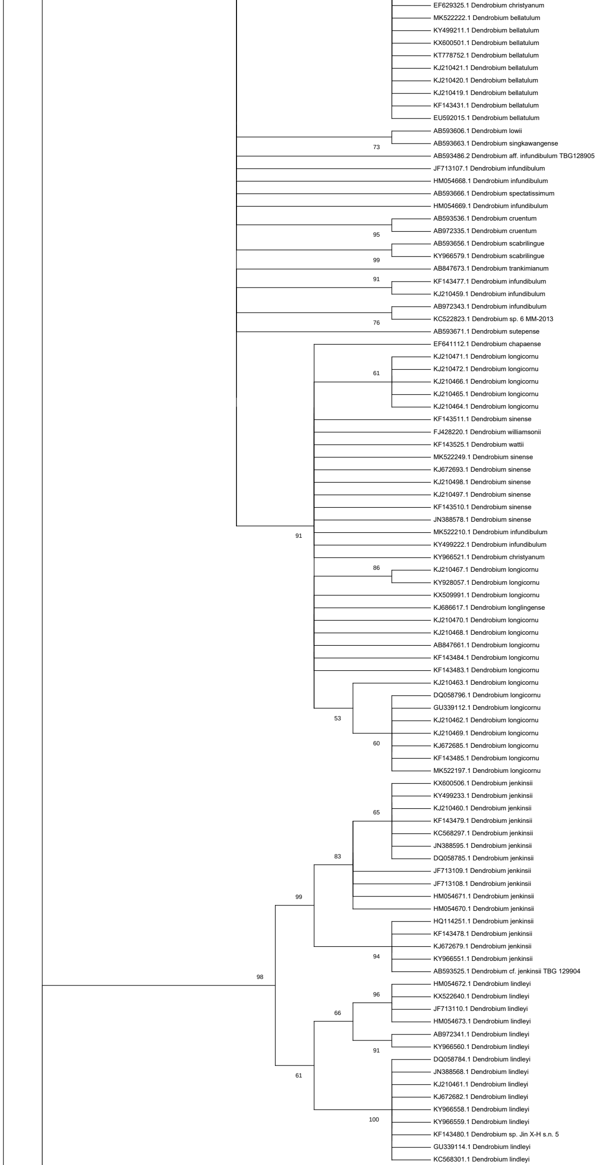

EF629325.1 Dendrobium christyanum
MK522222.1 Dendrobium bellatulum
KY499211.1 Dendrobium bellatulum
KX600501.1 Dendrobium bellatulum
KT778752.1 Dendrobium bellatulum
KJ210421.1 Dendrobium bellatulum
KJ210420.1 Dendrobium bellatulum
KJ210419.1 Dendrobium bellatulum
KF143431.1 Dendrobium bellatulum
EU592015.1 Dendrobium bellatulum
AB593606.1 Dendrobium lowii
AB593663.1 Dendrobium singkawangense
73
AB593486.2 Dendrobium aff. infundibulum TBG128905
JF713107.1 Dendrobium infundibulum
HM054668.1 Dendrobium infundibulum
AB593666.1 Dendrobium spectatissimum
HM054669.1 Dendrobium infundibulum
AB593536.1 Dendrobium cruentum
AB972335.1 Dendrobium cruentum
95
AB593656.1 Dendrobium scabrilingue
KY966579.1 Dendrobium scabrilingue
99
AB847673.1 Dendrobium trankimianum
91
KF143477.1 Dendrobium infundibulum
KJ210459.1 Dendrobium infundibulum
AB972343.1 Dendrobium infundibulum
KC522823.1 Dendrobium sp. 6 MM-2013
76
AB593671.1 Dendrobium sutepense
EF641112.1 Dendrobium chapaense
KJ210471.1 Dendrobium longicornu
61
KJ210472.1 Dendrobium longicornu
KJ210466.1 Dendrobium longicornu
KJ210465.1 Dendrobium longicornu
KJ210464.1 Dendrobium longicornu
KF143511.1 Dendrobium sinense
FJ428220.1 Dendrobium williamsonii
KF143525.1 Dendrobium wattii
MK522249.1 Dendrobium sinense
KJ672693.1 Dendrobium sinense
KJ210498.1 Dendrobium sinense
KJ210497.1 Dendrobium sinense
KF143510.1 Dendrobium sinense
JN388578.1 Dendrobium sinense
MK522210.1 Dendrobium infundibulum
91
KY499222.1 Dendrobium infundibulum
KY966521.1 Dendrobium christyanum
86
KJ210467.1 Dendrobium longicornu
KY928057.1 Dendrobium longicornu
KX509991.1 Dendrobium longicornu
KJ686617.1 Dendrobium longlingense
KJ210470.1 Dendrobium longicornu
KJ210468.1 Dendrobium longicornu
AB847661.1 Dendrobium longicornu
KF143484.1 Dendrobium longicornu
KF143483.1 Dendrobium longicornu
KJ210463.1 Dendrobium longicornu
DQ058796.1 Dendrobium longicornu
GU339112.1 Dendrobium longicornu
53
KJ210462.1 Dendrobium longicornu
KJ210469.1 Dendrobium longicornu
60
KJ672685.1 Dendrobium longicornu
KF143485.1 Dendrobium longicornu
MK522197.1 Dendrobium longicornu
KX600506.1 Dendrobium jenkinsii
KY499233.1 Dendrobium jenkinsii
KJ210460.1 Dendrobium jenkinsii
65
KF143479.1 Dendrobium jenkinsii
KC568297.1 Dendrobium jenkinsii
JN388595.1 Dendrobium jenkinsii
DQ058785.1 Dendrobium jenkinsii
83
JF713109.1 Dendrobium jenkinsii
JF713108.1 Dendrobium jenkinsii
99
HM054671.1 Dendrobium jenkinsii
HM054670.1 Dendrobium jenkinsii
HQ114251.1 Dendrobium jenkinsii
KF143478.1 Dendrobium jenkinsii
KJ672679.1 Dendrobium jenkinsii
94
KY966551.1 Dendrobium jenkinsii
AB593525.1 Dendrobium cf. jenkinsii TBG 129904
98
HM054672.1 Dendrobium lindleyi
96
KX522640.1 Dendrobium lindleyi
JF713110.1 Dendrobium lindleyi
66
HM054673.1 Dendrobium lindleyi
AB972341.1 Dendrobium lindleyi
KY966560.1 Dendrobium lindleyi
91
DQ058784.1 Dendrobium lindleyi
JN388568.1 Dendrobium lindleyi
61
KJ210461.1 Dendrobium lindleyi
KJ672682.1 Dendrobium lindleyi
KY966558.1 Dendrobium lindleyi
100
KY966559.1 Dendrobium lindleyi
KF143480.1 Dendrobium sp. Jin X-H s.n. 5
GU339114.1 Dendrobium lindleyi
KC568301.1 Dendrobium lindleyi

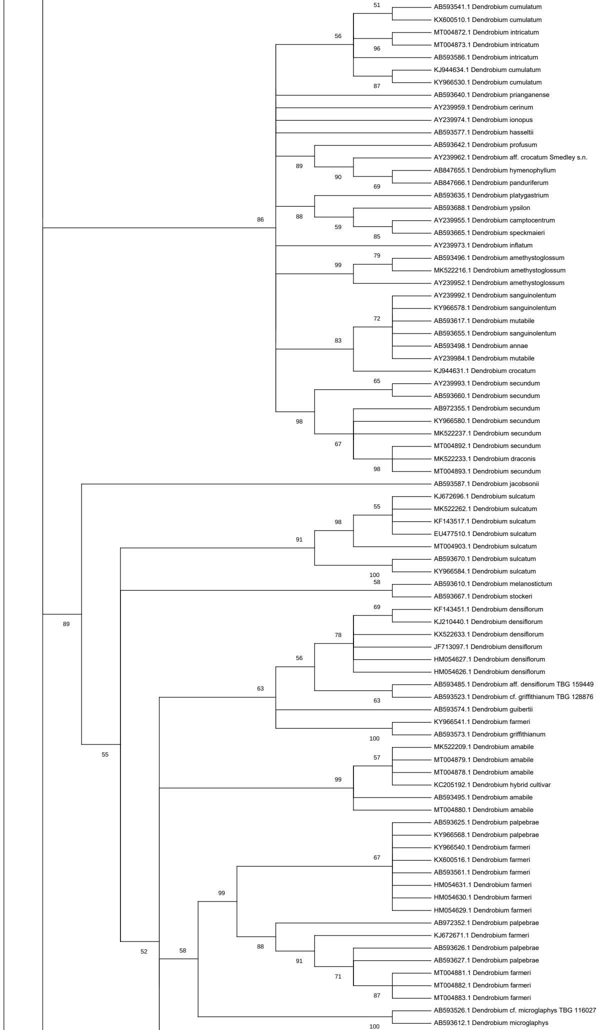
AB593541.1 Dendrobium cumulatum
KX600510.1 Dendrobium cumulatum
MT004872.1 Dendrobium intricatum
MT004873.1 Dendrobium intricatum
AB593586.1 Dendrobium intricatum
KJ944634.1 Dendrobium cumulatum
KY966530.1 Dendrobium cumulatum
AB593640.1 Dendrobium prianganense
AY239959.1 Dendrobium cerinum
AY239974.1 Dendrobium ionopus
AB593577.1 Dendrobium hasseltii
AB593642.1 Dendrobium profusum
AY239962.1 Dendrobium aff. crocatum Smedley s.n.
AB847655.1 Dendrobium hymenophyllum
AB847666.1 Dendrobium panduriferum
AB593635.1 Dendrobium platygastrium
AB593688.1 Dendrobium ypsilon
AY239955.1 Dendrobium camptocentrum
AB593665.1 Dendrobium speckmaieri
AY239973.1 Dendrobium inflatum
AB593496.1 Dendrobium amethystoglossum
MK522216.1 Dendrobium amethystoglossum
AY239952.1 Dendrobium amethystoglossum
AY239992.1 Dendrobium sanguinolentum
KY966578.1 Dendrobium sanguinolentum
AB593617.1 Dendrobium mutabile
AB593655.1 Dendrobium sanguinolentum
AB593498.1 Dendrobium annae
AY239984.1 Dendrobium mutabile
KJ944631.1 Dendrobium crocatum
AY239993.1 Dendrobium secundum
AB593660.1 Dendrobium secundum
AB972355.1 Dendrobium secundum
KY966580.1 Dendrobium secundum
MK522237.1 Dendrobium secundum
MT004892.1 Dendrobium secundum
MK522233.1 Dendrobium draconis
MT004893.1 Dendrobium secundum
AB593587.1 Dendrobium jacobsonii
KJ672696.1 Dendrobium sulcatum
MK522262.1 Dendrobium sulcatum
KF143517.1 Dendrobium sulcatum
EU477510.1 Dendrobium sulcatum
MT004903.1 Dendrobium sulcatum
AB593670.1 Dendrobium sulcatum
KY966584.1 Dendrobium sulcatum
AB593610.1 Dendrobium melanostictum
AB593667.1 Dendrobium stockeri
KF143451.1 Dendrobium densiflorum
KJ210440.1 Dendrobium densiflorum
KX522633.1 Dendrobium densiflorum
JF713097.1 Dendrobium densiflorum
HM054627.1 Dendrobium densiflorum
HM054626.1 Dendrobium densiflorum
AB593485.1 Dendrobium aff. densiflorum TBG 159449
AB593523.1 Dendrobium cf. griffithianum TBG 128876
AB593574.1 Dendrobium guibertii
KY966541.1 Dendrobium farmeri
AB593573.1 Dendrobium griffithianum
MK522209.1 Dendrobium amabile
MT004879.1 Dendrobium amabile
MT004878.1 Dendrobium amabile
KC205192.1 Dendrobium hybrid cultivar
AB593495.1 Dendrobium amabile
MT004880.1 Dendrobium amabile
AB593625.1 Dendrobium palpebrae
KY966568.1 Dendrobium palpebrae
KY966540.1 Dendrobium farmeri
KX600516.1 Dendrobium farmeri
AB593561.1 Dendrobium farmeri
HM054631.1 Dendrobium farmeri
HM054630.1 Dendrobium farmeri
HM054629.1 Dendrobium farmeri
AB972352.1 Dendrobium palpebrae
KJ672671.1 Dendrobium farmeri
AB593626.1 Dendrobium palpebrae
AB593627.1 Dendrobium palpebrae
MT004881.1 Dendrobium farmeri
MT004882.1 Dendrobium farmeri
MT004883.1 Dendrobium farmeri
AB593526.1 Dendrobium cf. microglaphys TBG 116027
AB593612.1 Dendrobium microglaphys

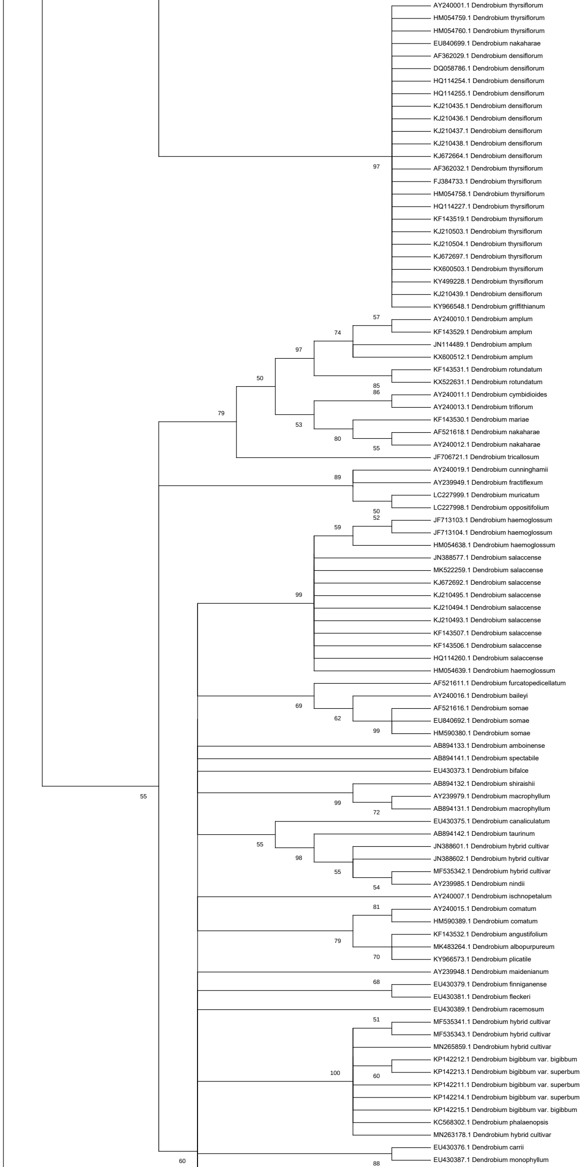

AY240001.1 Dendrobium thyrsiflorum
HM054759.1 Dendrobium thyrsiflorum
HM054760.1 Dendrobium thyrsiflorum
EU840699.1 Dendrobium nakaharae
AF362029.1 Dendrobium densiflorum
DQ058786.1 Dendrobium densiflorum
HQ114254.1 Dendrobium densiflorum
HQ114255.1 Dendrobium densiflorum
KJ210435.1 Dendrobium densiflorum
KJ210436.1 Dendrobium densiflorum
KJ210437.1 Dendrobium densiflorum
KJ210438.1 Dendrobium densiflorum
KJ672664.1 Dendrobium densiflorum
97
AF362032.1 Dendrobium thyrsiflorum
FJ384733.1 Dendrobium thyrsiflorum
HM054758.1 Dendrobium thyrsiflorum
HQ114227.1 Dendrobium thyrsiflorum
KF143519.1 Dendrobium thyrsiflorum
KJ210503.1 Dendrobium thyrsiflorum
KJ210504.1 Dendrobium thyrsiflorum
KJ672697.1 Dendrobium thyrsiflorum
KX600503.1 Dendrobium thyrsiflorum
KY499228.1 Dendrobium thyrsiflorum
KJ210439.1 Dendrobium densiflorum
KY966548.1 Dendrobium griffithianum
57
AY240010.1 Dendrobium amplum
74
KF143529.1 Dendrobium amplum
JN114489.1 Dendrobium amplum
97
KX600512.1 Dendrobium amplum
KF143531.1 Dendrobium rotundatum
50
KX522631.1 Dendrobium rotundatum
85
86
AY240011.1 Dendrobium cymbidioides
AY240013.1 Dendrobium triflorum
79
KF143530.1 Dendrobium mariae
53
AF521618.1 Dendrobium nakaharae
80
AY240012.1 Dendrobium nakaharae
55
JF706721.1 Dendrobium tricallosum
AY240019.1 Dendrobium cunninghamii
89
AY239949.1 Dendrobium fractiflexum
LC227999.1 Dendrobium muricatum
LC227998.1 Dendrobium oppositifolium
50
52
JF713103.1 Dendrobium haemoglossum
59
JF713104.1 Dendrobium haemoglossum
HM054638.1 Dendrobium haemoglossum
JN388577.1 Dendrobium salaccense
MK522259.1 Dendrobium salaccense
KJ672692.1 Dendrobium salaccense
99
KJ210495.1 Dendrobium salaccense
KJ210494.1 Dendrobium salaccense
KJ210493.1 Dendrobium salaccense
KF143507.1 Dendrobium salaccense
KF143506.1 Dendrobium salaccense
HQ114260.1 Dendrobium salaccense
HM054639.1 Dendrobium haemoglossum
AF521611.1 Dendrobium furcatopedicellatum
AY240016.1 Dendrobium baileyi
69
AF521616.1 Dendrobium somae
62
EU840692.1 Dendrobium somae
99
HM590380.1 Dendrobium somae
AB894133.1 Dendrobium amboinense
AB894141.1 Dendrobium spectabile
EU430373.1 Dendrobium bifalce
AB894132.1 Dendrobium shiraishii
AY239979.1 Dendrobium macrophyllum
55
99
AB894131.1 Dendrobium macrophyllum
72
EU430375.1 Dendrobium canaliculatum
AB894142.1 Dendrobium taurinum
55
JN388601.1 Dendrobium hybrid cultivar
98
JN388602.1 Dendrobium hybrid cultivar
MF535342.1 Dendrobium hybrid cultivar
55
AY239985.1 Dendrobium nindii
54
AY240007.1 Dendrobium ischnopetalum
81
AY240015.1 Dendrobium comatum
HM590389.1 Dendrobium comatum
KF143532.1 Dendrobium angustifolium
79
MK483264.1 Dendrobium albopurpureum
70
KY966573.1 Dendrobium plicatile
AY239948.1 Dendrobium maidenianum
68
EU430379.1 Dendrobium finniganense
EU430381.1 Dendrobium fleckeri
EU430389.1 Dendrobium racemosum
51
MF535341.1 Dendrobium hybrid cultivar
MF535343.1 Dendrobium hybrid cultivar
MN265859.1 Dendrobium hybrid cultivar
KP142212.1 Dendrobium bigibbum var. bigibbum
100
60
KP142213.1 Dendrobium bigibbum var. superbum
KP142211.1 Dendrobium bigibbum var. superbum
KP142214.1 Dendrobium bigibbum var. superbum
KP142215.1 Dendrobium bigibbum var. bigibbum
KC568302.1 Dendrobium phalaenopsis
MN263178.1 Dendrobium hybrid cultivar
EU430376.1 Dendrobium carrii
EU430387.1 Dendrobium monophyllum
60
88

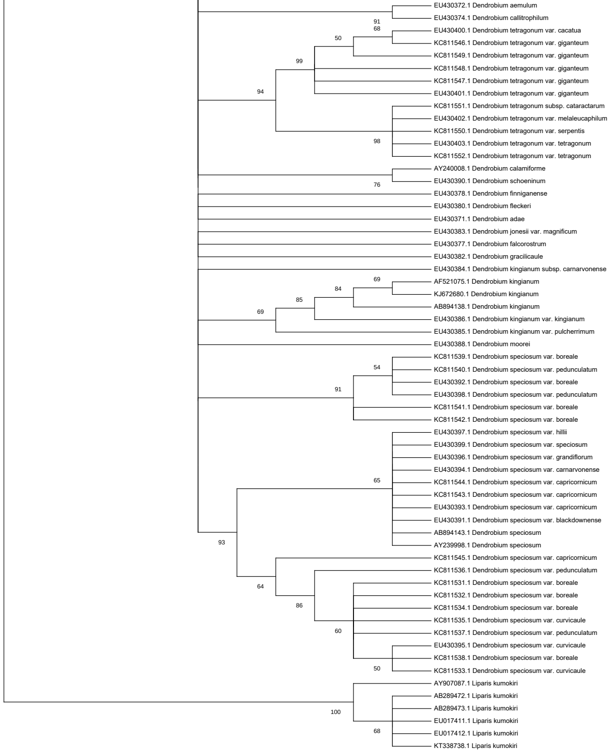
EU430372.1 Dendrobium aemulum
EU430374.1 Dendrobium callitrophilum
EU430400.1 Dendrobium tetragonum var. cacatua
KC811546.1 Dendrobium tetragonum var. giganteum
KC811549.1 Dendrobium tetragonum var. giganteum
KC811548.1 Dendrobium tetragonum var. giganteum
KC811547.1 Dendrobium tetragonum var. giganteum
EU430401.1 Dendrobium tetragonum var. giganteum
KC811551.1 Dendrobium tetragonum subsp. cataractarum
EU430402.1 Dendrobium tetragonum var. melaleucaphilum
KC811550.1 Dendrobium tetragonum var. serpentis
EU430403.1 Dendrobium tetragonum var. tetragonum
KC811552.1 Dendrobium tetragonum var. tetragonum
AY240008.1 Dendrobium calamiforme
EU430390.1 Dendrobium schoeninum
EU430378.1 Dendrobium finniganense
EU430380.1 Dendrobium fleckeri
EU430371.1 Dendrobium adae
EU430383.1 Dendrobium jonesii var. magnificum
EU430377.1 Dendrobium falcorostrum
EU430382.1 Dendrobium gracilicaule
EU430384.1 Dendrobium kingianum subsp. carnarvonense
AF521075.1 Dendrobium kingianum
KJ672680.1 Dendrobium kingianum
AB894138.1 Dendrobium kingianum
EU430386.1 Dendrobium kingianum var. kingianum
EU430385.1 Dendrobium kingianum var. pulcherrimum
EU430388.1 Dendrobium moorei
KC811539.1 Dendrobium speciosum var. boreale
KC811540.1 Dendrobium speciosum var. pedunculatum
EU430392.1 Dendrobium speciosum var. boreale
EU430398.1 Dendrobium speciosum var. pedunculatum
KC811541.1 Dendrobium speciosum var. boreale
KC811542.1 Dendrobium speciosum var. boreale
EU430397.1 Dendrobium speciosum var. hillii
EU430399.1 Dendrobium speciosum var. speciosum
EU430396.1 Dendrobium speciosum var. grandiflorum
EU430394.1 Dendrobium speciosum var. carnarvonense
KC811544.1 Dendrobium speciosum var. capricornicum
KC811543.1 Dendrobium speciosum var. capricornicum
EU430393.1 Dendrobium speciosum var. capricornicum
EU430391.1 Dendrobium speciosum var. blackdownense
AB894143.1 Dendrobium speciosum
AY239998.1 Dendrobium speciosum
KC811545.1 Dendrobium speciosum var. capricornicum
KC811536.1 Dendrobium speciosum var. pedunculatum
KC811531.1 Dendrobium speciosum var. boreale
KC811532.1 Dendrobium speciosum var. boreale
KC811534.1 Dendrobium speciosum var. boreale
KC811535.1 Dendrobium speciosum var. curvicaule
KC811537.1 Dendrobium speciosum var. pedunculatum
EU430395.1 Dendrobium speciosum var. curvicaule
KC811538.1 Dendrobium speciosum var. boreale
KC811533.1 Dendrobium speciosum var. curvicaule
AY907087.1 Liparis kumokiri
AB289472.1 Liparis kumokiri
AB289473.1 Liparis kumokiri
EU017411.1 Liparis kumokiri
EU017412.1 Liparis kumokiri
KT338738.1 Liparis kumokiri
91
68
50
99
94
98
76
69
84
85
69
54
91
65
93
64
86
60
50
100
68
