# Supplementary material for: Assessing the reliability of medicinal Dendrobium sequences in GenBank for botanical species identification
Source: Sci Rep. 2021 Feb 9;11:3439. doi: 10.1038/s41598-021-82385-z (PMC7873228; doi:10.1038/s41598-021-82385-z)
Supplement: Supplementary file 9 — Supplementary Figure S7. [file 41598_2021_82385_MOESM9_ESM.pdf]

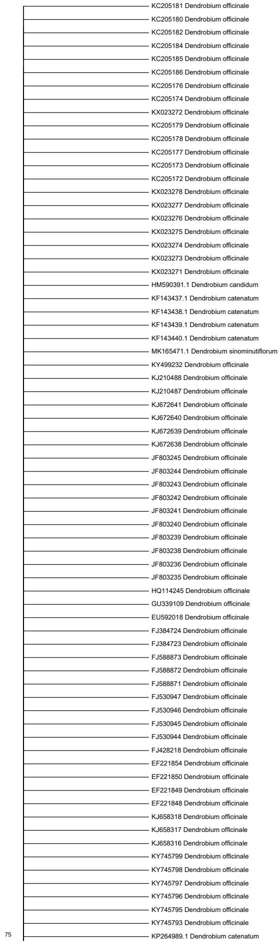
KC205181 Dendrobium officinale
KC205180 Dendrobium officinale
KC205182 Dendrobium officinale
KC205184 Dendrobium officinale
KC205185 Dendrobium officinale
KC205186 Dendrobium officinale
KC205176 Dendrobium officinale
KC205174 Dendrobium officinale
KX023272 Dendrobium officinale
KC205179 Dendrobium officinale
KC205178 Dendrobium officinale
KC205177 Dendrobium officinale
KC205173 Dendrobium officinale
KC205172 Dendrobium officinale
KX023278 Dendrobium officinale
KX023277 Dendrobium officinale
KX023276 Dendrobium officinale
KX023275 Dendrobium officinale
KX023274 Dendrobium officinale
KX023273 Dendrobium officinale
KX023271 Dendrobium officinale
HM590391.1 Dendrobium candidum
KF143437.1 Dendrobium catenatum
KF143438.1 Dendrobium catenatum
KF143439.1 Dendrobium catenatum
KF143440.1 Dendrobium catenatum
MK165471.1 Dendrobium sinominutiflorum
KY499232 Dendrobium officinale
KJ210488 Dendrobium officinale
KJ210487 Dendrobium officinale
KJ672641 Dendrobium officinale
KJ672640 Dendrobium officinale
KJ672639 Dendrobium officinale
KJ672638 Dendrobium officinale
JF803245 Dendrobium officinale
JF803244 Dendrobium officinale
JF803243 Dendrobium officinale
JF803242 Dendrobium officinale
JF803241 Dendrobium officinale
JF803240 Dendrobium officinale
JF803239 Dendrobium officinale
JF803238 Dendrobium officinale
JF803236 Dendrobium officinale
JF803235 Dendrobium officinale
HQ114245 Dendrobium officinale
GU339109 Dendrobium officinale
EU592018 Dendrobium officinale
FJ384724 Dendrobium officinale
FJ384723 Dendrobium officinale
FJ588873 Dendrobium officinale
FJ588872 Dendrobium officinale
FJ588871 Dendrobium officinale
FJ530947 Dendrobium officinale
FJ530946 Dendrobium officinale
FJ530945 Dendrobium officinale
FJ530944 Dendrobium officinale
FJ428218 Dendrobium officinale
EF221854 Dendrobium officinale
EF221850 Dendrobium officinale
EF221849 Dendrobium officinale
EF221848 Dendrobium officinale
KJ658318 Dendrobium officinale
KJ658317 Dendrobium officinale
KJ658316 Dendrobium officinale
KY745799 Dendrobium officinale
KY745798 Dendrobium officinale
KY745797 Dendrobium officinale
KY745796 Dendrobium officinale
KY745795 Dendrobium officinale
KY745793 Dendrobium officinale
75
KP264989.1 Dendrobium catenatum

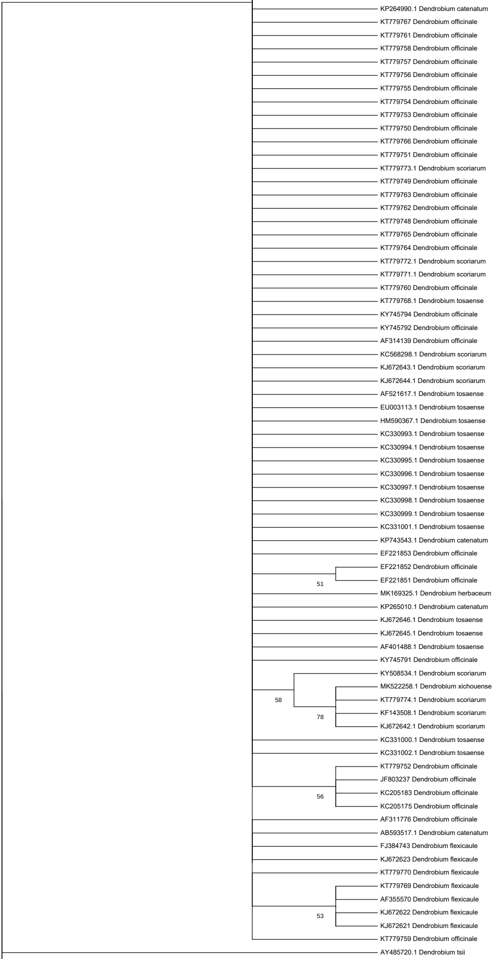
KP264990.1 Dendrobium catenatum
KT779767 Dendrobium officinale
KT779761 Dendrobium officinale
KT779758 Dendrobium officinale
KT779757 Dendrobium officinale
KT779756 Dendrobium officinale
KT779755 Dendrobium officinale
KT779754 Dendrobium officinale
KT779753 Dendrobium officinale
KT779750 Dendrobium officinale
KT779766 Dendrobium officinale
KT779751 Dendrobium officinale
KT779773.1 Dendrobium scoriarum
KT779749 Dendrobium officinale
KT779763 Dendrobium officinale
KT779762 Dendrobium officinale
KT779748 Dendrobium officinale
KT779765 Dendrobium officinale
KT779764 Dendrobium officinale
KT779772.1 Dendrobium scoriarum
KT779771.1 Dendrobium scoriarum
KT779760 Dendrobium officinale
KT779768.1 Dendrobium tosaense
KY745794 Dendrobium officinale
KY745792 Dendrobium officinale
AF314139 Dendrobium officinale
KC568298.1 Dendrobium scoriarum
KJ672643.1 Dendrobium scoriarum
KJ672644.1 Dendrobium scoriarum
AF521617.1 Dendrobium tosaense
EU003113.1 Dendrobium tosaense
HM590367.1 Dendrobium tosaense
KC330993.1 Dendrobium tosaense
KC330994.1 Dendrobium tosaense
KC330995.1 Dendrobium tosaense
KC330996.1 Dendrobium tosaense
KC330997.1 Dendrobium tosaense
KC330998.1 Dendrobium tosaense
KC330999.1 Dendrobium tosaense
KC331001.1 Dendrobium tosaense
KP743543.1 Dendrobium catenatum
EF221853 Dendrobium officinale
EF221852 Dendrobium officinale
51
EF221851 Dendrobium officinale
MK169325.1 Dendrobium herbaceum
KP265010.1 Dendrobium catenatum
KJ672646.1 Dendrobium tosaense
KJ672645.1 Dendrobium tosaense
AF401488.1 Dendrobium tosaense
KY745791 Dendrobium officinale
KY508534.1 Dendrobium scoriarum
MK522258.1 Dendrobium xichouense
58
KT779774.1 Dendrobium scoriarum
KF143508.1 Dendrobium scoriarum
78
KJ672642.1 Dendrobium scoriarum
KC331000.1 Dendrobium tosaense
KC331002.1 Dendrobium tosaense
KT779752 Dendrobium officinale
JF803237 Dendrobium officinale
KC205183 Dendrobium officinale
56
KC205175 Dendrobium officinale
AF311776 Dendrobium officinale
AB593517.1 Dendrobium catenatum
FJ384743 Dendrobium flexicaule
KJ672623 Dendrobium flexicaule
KT779770 Dendrobium flexicaule
KT779769 Dendrobium flexicaule
AF355570 Dendrobium flexicaule
KJ672622 Dendrobium flexicaule
53
KJ672621 Dendrobium flexicaule
KT779759 Dendrobium officinale
AY485720.1 Dendrobium tsii

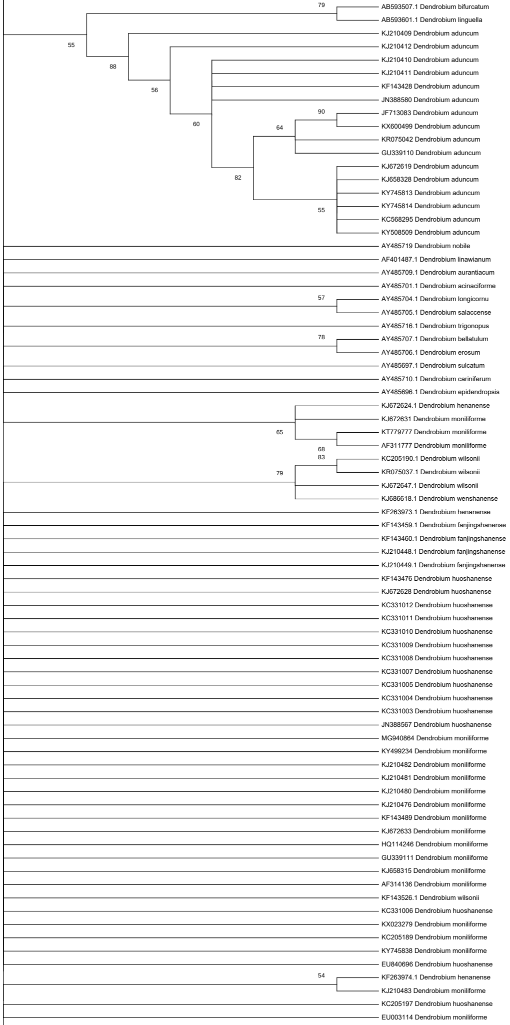
AB593507.1 Dendrobium bifurcatum
AB593601.1 Dendrobium linguella
KJ210409 Dendrobium aduncum
KJ210412 Dendrobium aduncum
KJ210410 Dendrobium aduncum
KJ210411 Dendrobium aduncum
KF143428 Dendrobium aduncum
JN388580 Dendrobium aduncum
JF713083 Dendrobium aduncum
KX600499 Dendrobium aduncum
KR075042 Dendrobium aduncum
GU339110 Dendrobium aduncum
KJ672619 Dendrobium aduncum
KJ658328 Dendrobium aduncum
KY745813 Dendrobium aduncum
KY745814 Dendrobium aduncum
KC568295 Dendrobium aduncum
KY508509 Dendrobium aduncum
AY485719 Dendrobium nobile
AF401487.1 Dendrobium linawianum
AY485709.1 Dendrobium aurantiacum
AY485701.1 Dendrobium acinaciforme
AY485704.1 Dendrobium longicornu
AY485705.1 Dendrobium salaccense
AY485716.1 Dendrobium trigonopus
AY485707.1 Dendrobium bellatulum
AY485706.1 Dendrobium erosum
AY485697.1 Dendrobium sulcatum
AY485710.1 Dendrobium cariniferum
AY485696.1 Dendrobium epidendropsis
KJ672624.1 Dendrobium henanense
KJ672631 Dendrobium moniliforme
KT779777 Dendrobium moniliforme
AF311777 Dendrobium moniliforme
KC205190.1 Dendrobium wilsonii
KR075037.1 Dendrobium wilsonii
KJ672647.1 Dendrobium wilsonii
KJ686618.1 Dendrobium wenshanense
KF263973.1 Dendrobium henanense
KF143459.1 Dendrobium fanjingshanense
KF143460.1 Dendrobium fanjingshanense
KJ210448.1 Dendrobium fanjingshanense
KJ210449.1 Dendrobium fanjingshanense
KF143476 Dendrobium huoshanense
KJ672628 Dendrobium huoshanense
KC331012 Dendrobium huoshanense
KC331011 Dendrobium huoshanense
KC331010 Dendrobium huoshanense
KC331009 Dendrobium huoshanense
KC331008 Dendrobium huoshanense
KC331007 Dendrobium huoshanense
KC331005 Dendrobium huoshanense
KC331004 Dendrobium huoshanense
KC331003 Dendrobium huoshanense
JN388567 Dendrobium huoshanense
MG940864 Dendrobium moniliforme
KY499234 Dendrobium moniliforme
KJ210482 Dendrobium moniliforme
KJ210481 Dendrobium moniliforme
KJ210480 Dendrobium moniliforme
KJ210476 Dendrobium moniliforme
KF143489 Dendrobium moniliforme
KJ672633 Dendrobium moniliforme
HQ114246 Dendrobium moniliforme
GU339111 Dendrobium moniliforme
KJ658315 Dendrobium moniliforme
AF314136 Dendrobium moniliforme
KF143526.1 Dendrobium wilsonii
KC331006 Dendrobium huoshanense
KX023279 Dendrobium moniliforme
KC205189 Dendrobium moniliforme
KY745838 Dendrobium moniliforme
EU840696 Dendrobium huoshanense
KF263974.1 Dendrobium henanense
KJ210483 Dendrobium moniliforme
KC205197 Dendrobium huoshanense
EU003114 Dendrobium moniliforme

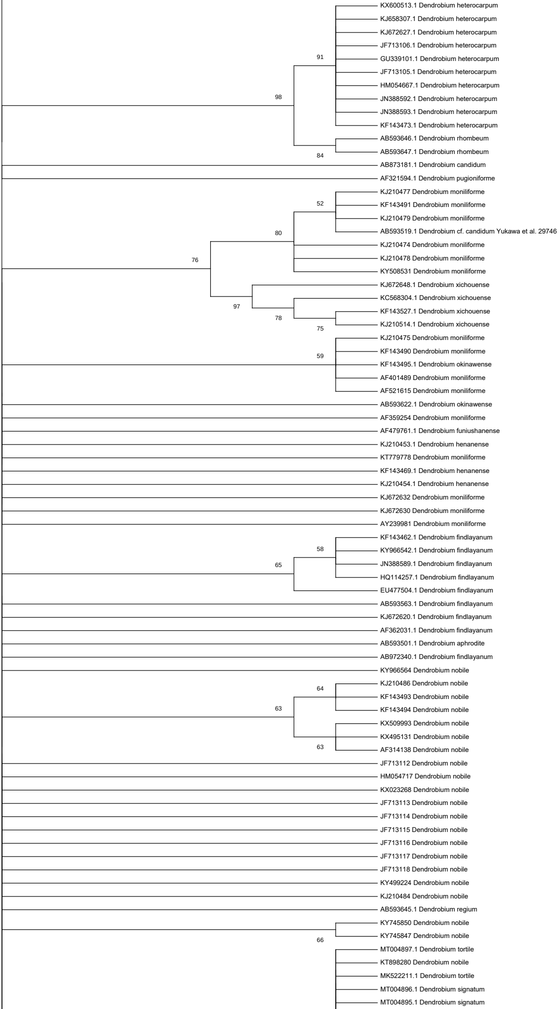
KX600513.1 Dendrobium heterocarpum
KJ658307.1 Dendrobium heterocarpum
KJ672627.1 Dendrobium heterocarpum
JF713106.1 Dendrobium heterocarpum
GU339101.1 Dendrobium heterocarpum
JF713105.1 Dendrobium heterocarpum
HM054667.1 Dendrobium heterocarpum
JN388592.1 Dendrobium heterocarpum
JN388593.1 Dendrobium heterocarpum
KF143473.1 Dendrobium heterocarpum
AB593646.1 Dendrobium rhombeum
AB593647.1 Dendrobium rhombeum
AB873181.1 Dendrobium candidum
AF321594.1 Dendrobium pugioniforme
KJ210477 Dendrobium moniliforme
KF143491 Dendrobium moniliforme
KJ210479 Dendrobium moniliforme
AB593519.1 Dendrobium cf. candidum Yukawa et al. 29746
KJ210474 Dendrobium moniliforme
KJ210478 Dendrobium moniliforme
KY508531 Dendrobium moniliforme
KJ672648.1 Dendrobium xichouense
KC568304.1 Dendrobium xichouense
KF143527.1 Dendrobium xichouense
KJ210514.1 Dendrobium xichouense
KJ210475 Dendrobium moniliforme
KF143490 Dendrobium moniliforme
KF143495.1 Dendrobium okinawense
AF401489 Dendrobium moniliforme
AF521615 Dendrobium moniliforme
AB593622.1 Dendrobium okinawense
AF359254 Dendrobium moniliforme
AF479761.1 Dendrobium funiushanense
KJ210453.1 Dendrobium henanense
KT779778 Dendrobium moniliforme
KF143469.1 Dendrobium henanense
KJ210454.1 Dendrobium henanense
KJ672632 Dendrobium moniliforme
KJ672630 Dendrobium moniliforme
AY239981 Dendrobium moniliforme
KF143462.1 Dendrobium findlayanum
KY966542.1 Dendrobium findlayanum
JN388589.1 Dendrobium findlayanum
HQ114257.1 Dendrobium findlayanum
EU477504.1 Dendrobium findlayanum
AB593563.1 Dendrobium findlayanum
KJ672620.1 Dendrobium findlayanum
AF362031.1 Dendrobium findlayanum
AB593501.1 Dendrobium aphrodite
AB972340.1 Dendrobium findlayanum
KY966564 Dendrobium nobile
KJ210486 Dendrobium nobile
KF143493 Dendrobium nobile
KF143494 Dendrobium nobile
KX509993 Dendrobium nobile
KX495131 Dendrobium nobile
AF314138 Dendrobium nobile
JF713112 Dendrobium nobile
HM054717 Dendrobium nobile
KX023268 Dendrobium nobile
JF713113 Dendrobium nobile
JF713114 Dendrobium nobile
JF713115 Dendrobium nobile
JF713116 Dendrobium nobile
JF713117 Dendrobium nobile
JF713118 Dendrobium nobile
KY499224 Dendrobium nobile
KJ210484 Dendrobium nobile
AB593645.1 Dendrobium regium
KY745850 Dendrobium nobile
KY745847 Dendrobium nobile
MT004897.1 Dendrobium tortile
KT898280 Dendrobium nobile
MK522211.1 Dendrobium tortile
MT004896.1 Dendrobium signatum
MT004895.1 Dendrobium signatum

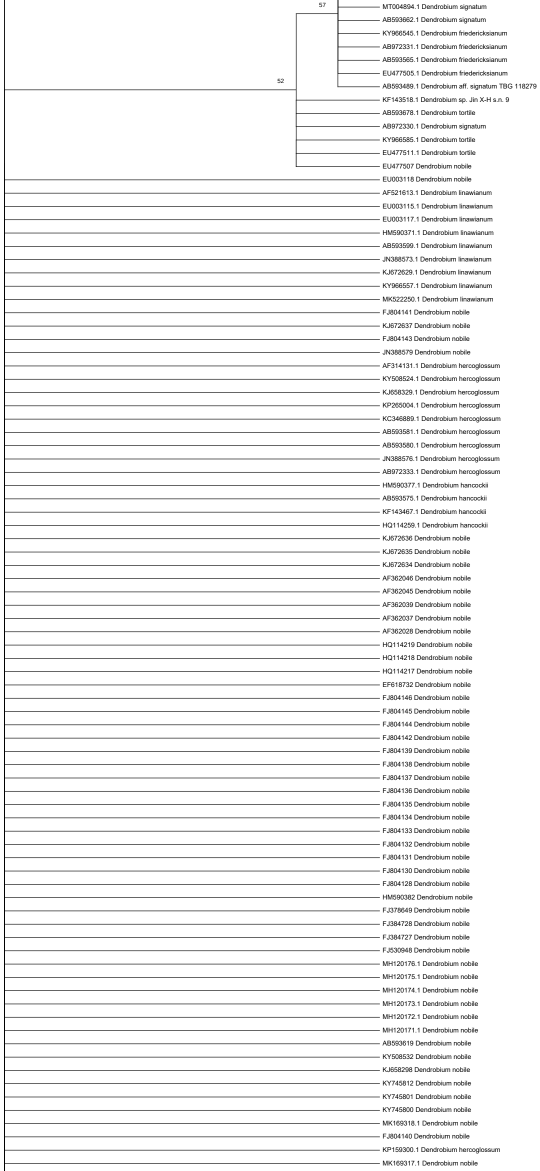

57
52
MT004894.1 Dendrobium signatum
AB593662.1 Dendrobium signatum
KY966545.1 Dendrobium friedericksianum
AB972331.1 Dendrobium friedericksianum
AB593565.1 Dendrobium friedericksianum
EU477505.1 Dendrobium friedericksianum
AB593489.1 Dendrobium aff. signatum TBG 118279
KF143518.1 Dendrobium sp. Jin X-H s.n. 9
AB593678.1 Dendrobium tortile
AB972330.1 Dendrobium signatum
KY966585.1 Dendrobium tortile
EU477511.1 Dendrobium tortile
EU477507 Dendrobium nobile
EU003118 Dendrobium nobile
AF521613.1 Dendrobium linawianum
EU003115.1 Dendrobium linawianum
EU003117.1 Dendrobium linawianum
HM590371.1 Dendrobium linawianum
AB593599.1 Dendrobium linawianum
JN388573.1 Dendrobium linawianum
KJ672629.1 Dendrobium linawianum
KY966557.1 Dendrobium linawianum
MK522250.1 Dendrobium linawianum
FJ804141 Dendrobium nobile
KJ672637 Dendrobium nobile
FJ804143 Dendrobium nobile
JN388579 Dendrobium nobile
AF314131.1 Dendrobium hercoglossum
KY508524.1 Dendrobium hercoglossum
KJ658329.1 Dendrobium hercoglossum
KP265004.1 Dendrobium hercoglossum
KC346889.1 Dendrobium hercoglossum
AB593581.1 Dendrobium hercoglossum
AB593580.1 Dendrobium hercoglossum
JN388576.1 Dendrobium hercoglossum
AB972333.1 Dendrobium hercoglossum
HM590377.1 Dendrobium hancockii
AB593575.1 Dendrobium hancockii
KF143467.1 Dendrobium hancockii
HQ114259.1 Dendrobium hancockii
KJ672636 Dendrobium nobile
KJ672635 Dendrobium nobile
KJ672634 Dendrobium nobile
AF362046 Dendrobium nobile
AF362045 Dendrobium nobile
AF362039 Dendrobium nobile
AF362037 Dendrobium nobile
AF362028 Dendrobium nobile
HQ114219 Dendrobium nobile
HQ114218 Dendrobium nobile
HQ114217 Dendrobium nobile
EF618732 Dendrobium nobile
FJ804146 Dendrobium nobile
FJ804145 Dendrobium nobile
FJ804144 Dendrobium nobile
FJ804142 Dendrobium nobile
FJ804139 Dendrobium nobile
FJ804138 Dendrobium nobile
FJ804137 Dendrobium nobile
FJ804136 Dendrobium nobile
FJ804135 Dendrobium nobile
FJ804134 Dendrobium nobile
FJ804133 Dendrobium nobile
FJ804132 Dendrobium nobile
FJ804131 Dendrobium nobile
FJ804130 Dendrobium nobile
FJ804128 Dendrobium nobile
HM590382 Dendrobium nobile
FJ378649 Dendrobium nobile
FJ384728 Dendrobium nobile
FJ384727 Dendrobium nobile
FJ530948 Dendrobium nobile
MH120176.1 Dendrobium nobile
MH120175.1 Dendrobium nobile
MH120174.1 Dendrobium nobile
MH120173.1 Dendrobium nobile
MH120172.1 Dendrobium nobile
MH120171.1 Dendrobium nobile
AB593619 Dendrobium nobile
KY508532 Dendrobium nobile
KJ658298 Dendrobium nobile
KY745812 Dendrobium nobile
KY745801 Dendrobium nobile
KY745800 Dendrobium nobile
MK169318.1 Dendrobium nobile
FJ804140 Dendrobium nobile
KP159300.1 Dendrobium hercoglossum
MK169317.1 Dendrobium nobile

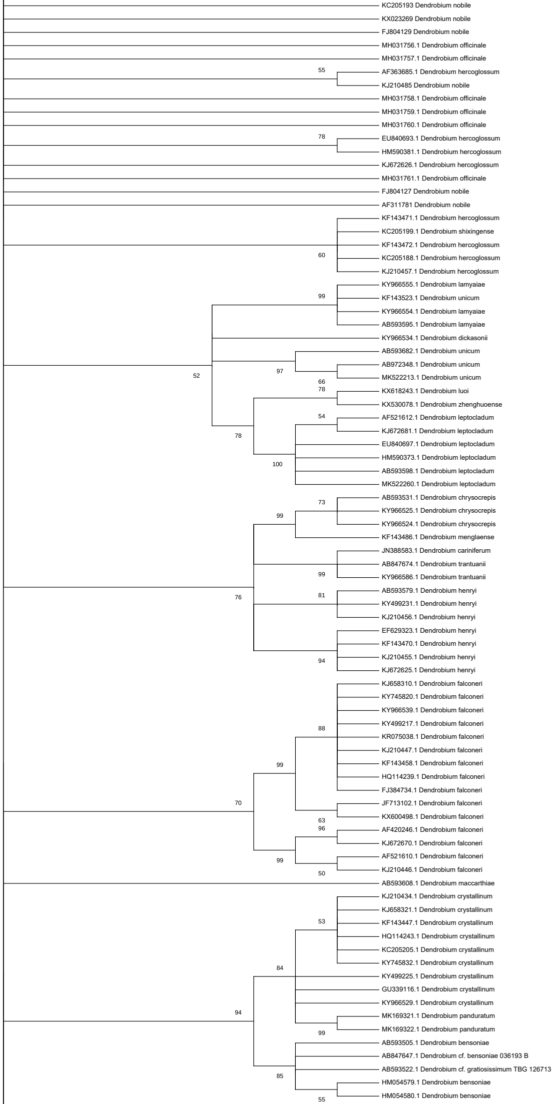
KC205193 Dendrobium nobile
KX023269 Dendrobium nobile
FJ804129 Dendrobium nobile
MH031756.1 Dendrobium officinale
MH031757.1 Dendrobium officinale
55
AF363685.1 Dendrobium hercoglossum
KJ210485 Dendrobium nobile
MH031758.1 Dendrobium officinale
MH031759.1 Dendrobium officinale
MH031760.1 Dendrobium officinale
78
EU840693.1 Dendrobium hercoglossum
HM590381.1 Dendrobium hercoglossum
KJ672626.1 Dendrobium hercoglossum
MH031761.1 Dendrobium officinale
FJ804127 Dendrobium nobile
AF311781 Dendrobium nobile
KF143471.1 Dendrobium hercoglossum
KC205199.1 Dendrobium shixingense
KF143472.1 Dendrobium hercoglossum
KC205188.1 Dendrobium hercoglossum
60
KJ210457.1 Dendrobium hercoglossum
KY966555.1 Dendrobium lamyaiae
99
KF143523.1 Dendrobium unicum
KY966554.1 Dendrobium lamyaiae
AB593595.1 Dendrobium lamyaiae
KY966534.1 Dendrobium dickasonii
AB593682.1 Dendrobium unicum
AB972348.1 Dendrobium unicum
97
52
MK522213.1 Dendrobium unicum
66
78
KX618243.1 Dendrobium luoi
KX530078.1 Dendrobium zhenghuoense
54
AF521612.1 Dendrobium leptocladum
KJ672681.1 Dendrobium leptocladum
78
EU840697.1 Dendrobium leptocladum
HM590373.1 Dendrobium leptocladum
100
AB593598.1 Dendrobium leptocladum
MK522260.1 Dendrobium leptocladum
AB593531.1 Dendrobium chrysocrepis
73
KY966525.1 Dendrobium chrysocrepis
99
KY966524.1 Dendrobium chrysocrepis
KF143486.1 Dendrobium menglaense
JN388583.1 Dendrobium cariniferum
AB847674.1 Dendrobium trantuanii
99
KY966586.1 Dendrobium trantuanii
AB593579.1 Dendrobium henryi
81
76
KY499231.1 Dendrobium henryi
KJ210456.1 Dendrobium henryi
EF629323.1 Dendrobium henryi
KF143470.1 Dendrobium henryi
KJ210455.1 Dendrobium henryi
94
KJ672625.1 Dendrobium henryi
KJ658310.1 Dendrobium falconeri
KY745820.1 Dendrobium falconeri
KY966539.1 Dendrobium falconeri
KY499217.1 Dendrobium falconeri
88
KR075038.1 Dendrobium falconeri
KJ210447.1 Dendrobium falconeri
99
KF143458.1 Dendrobium falconeri
HQ114239.1 Dendrobium falconeri
FJ384734.1 Dendrobium falconeri
70
JF713102.1 Dendrobium falconeri
KX600498.1 Dendrobium falconeri
63
96
AF420246.1 Dendrobium falconeri
KJ672670.1 Dendrobium falconeri
AF521610.1 Dendrobium falconeri
99
50
KJ210446.1 Dendrobium falconeri
AB593608.1 Dendrobium maccarthiae
KJ210434.1 Dendrobium crystallinum
KJ658321.1 Dendrobium crystallinum
53
KF143447.1 Dendrobium crystallinum
HQ114243.1 Dendrobium crystallinum
KC205205.1 Dendrobium crystallinum
KY745832.1 Dendrobium crystallinum
84
KY499225.1 Dendrobium crystallinum
GU339116.1 Dendrobium crystallinum
94
KY966529.1 Dendrobium crystallinum
MK169321.1 Dendrobium panduratum
MK169322.1 Dendrobium panduratum
99
AB593505.1 Dendrobium bensoniae
AB847647.1 Dendrobium cf. bensoniae 036193 B
AB593522.1 Dendrobium cf. gratiosissimum TBG 126713
85
HM054579.1 Dendrobium bensoniae
HM054580.1 Dendrobium bensoniae
55

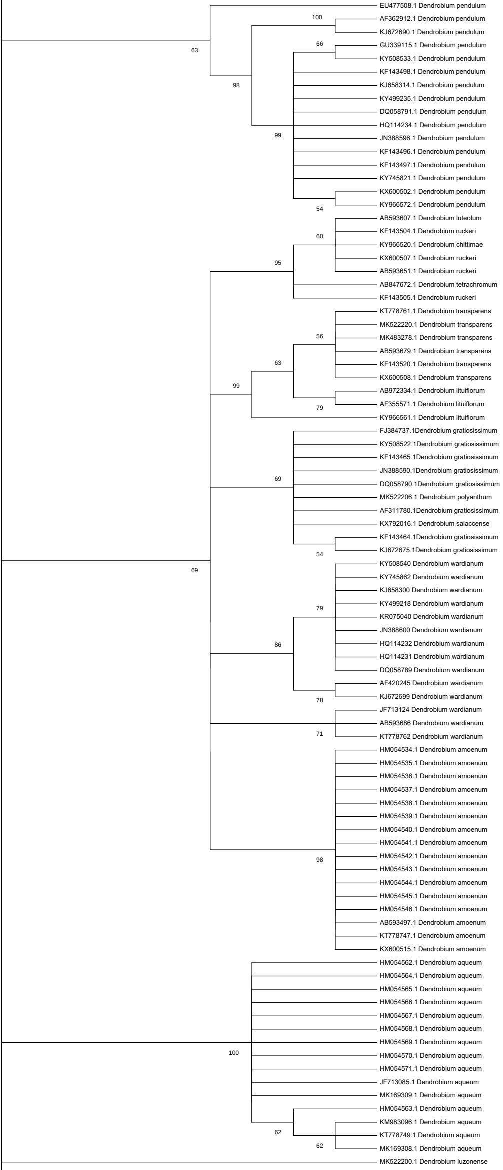
EU477508.1 Dendrobium pendulum
AF362912.1 Dendrobium pendulum
KJ672690.1 Dendrobium pendulum
GU339115.1 Dendrobium pendulum
KY508533.1 Dendrobium pendulum
KF143498.1 Dendrobium pendulum
KJ658314.1 Dendrobium pendulum
KY499235.1 Dendrobium pendulum
DQ058791.1 Dendrobium pendulum
HQ114234.1 Dendrobium pendulum
JN388596.1 Dendrobium pendulum
KF143496.1 Dendrobium pendulum
KF143497.1 Dendrobium pendulum
KY745821.1 Dendrobium pendulum
KX600502.1 Dendrobium pendulum
KY966572.1 Dendrobium pendulum
AB593607.1 Dendrobium luteolum
KF143504.1 Dendrobium ruckeri
KY966520.1 Dendrobium chittimae
KX600507.1 Dendrobium ruckeri
AB593651.1 Dendrobium ruckeri
AB847672.1 Dendrobium tetrachromum
KF143505.1 Dendrobium ruckeri
KT778761.1 Dendrobium transparens
MK522220.1 Dendrobium transparens
MK483278.1 Dendrobium transparens
AB593679.1 Dendrobium transparens
KF143520.1 Dendrobium transparens
KX600508.1 Dendrobium transparens
AB972334.1 Dendrobium lituiflorum
AF355571.1 Dendrobium lituiflorum
KY966561.1 Dendrobium lituiflorum
FJ384737.1Dendrobium gratiosissimum
KY508522.1Dendrobium gratiosissimum
KF143465.1Dendrobium gratiosissimum
JN388590.1Dendrobium gratiosissimum
DQ058790.1Dendrobium gratiosissimum
MK522206.1 Dendrobium polyanthum
AF311780.1Dendrobium gratiosissimum
KX792016.1 Dendrobium salaccense
KF143464.1Dendrobium gratiosissimum
KJ672675.1Dendrobium gratiosissimum
KY508540 Dendrobium wardianum
KY745862 Dendrobium wardianum
KJ658300 Dendrobium wardianum
KY499218 Dendrobium wardianum
KR075040 Dendrobium wardianum
JN388600 Dendrobium wardianum
HQ114232 Dendrobium wardianum
HQ114231 Dendrobium wardianum
DQ058789 Dendrobium wardianum
AF420245 Dendrobium wardianum
KJ672699 Dendrobium wardianum
JF713124 Dendrobium wardianum
AB593686 Dendrobium wardianum
KT778762 Dendrobium wardianum
HM054534.1 Dendrobium amoenum
HM054535.1 Dendrobium amoenum
HM054536.1 Dendrobium amoenum
HM054537.1 Dendrobium amoenum
HM054538.1 Dendrobium amoenum
HM054539.1 Dendrobium amoenum
HM054540.1 Dendrobium amoenum
HM054541.1 Dendrobium amoenum
HM054542.1 Dendrobium amoenum
HM054543.1 Dendrobium amoenum
HM054544.1 Dendrobium amoenum
HM054545.1 Dendrobium amoenum
HM054546.1 Dendrobium amoenum
AB593497.1 Dendrobium amoenum
KT778747.1 Dendrobium amoenum
KX600515.1 Dendrobium amoenum
HM054562.1 Dendrobium aqueum
HM054564.1 Dendrobium aqueum
HM054565.1 Dendrobium aqueum
HM054566.1 Dendrobium aqueum
HM054567.1 Dendrobium aqueum
HM054568.1 Dendrobium aqueum
HM054569.1 Dendrobium aqueum
HM054570.1 Dendrobium aqueum
HM054571.1 Dendrobium aqueum
JF713085.1 Dendrobium aqueum
MK169309.1 Dendrobium aqueum
HM054563.1 Dendrobium aqueum
KM983096.1 Dendrobium aqueum
KT778749.1 Dendrobium aqueum
MK169308.1 Dendrobium aqueum
MK522200.1 Dendrobium luzonense
100
66
63
98
99
54
60
95
56
63
99
79
69
54
69
79
86
78
71
98
100
62
62

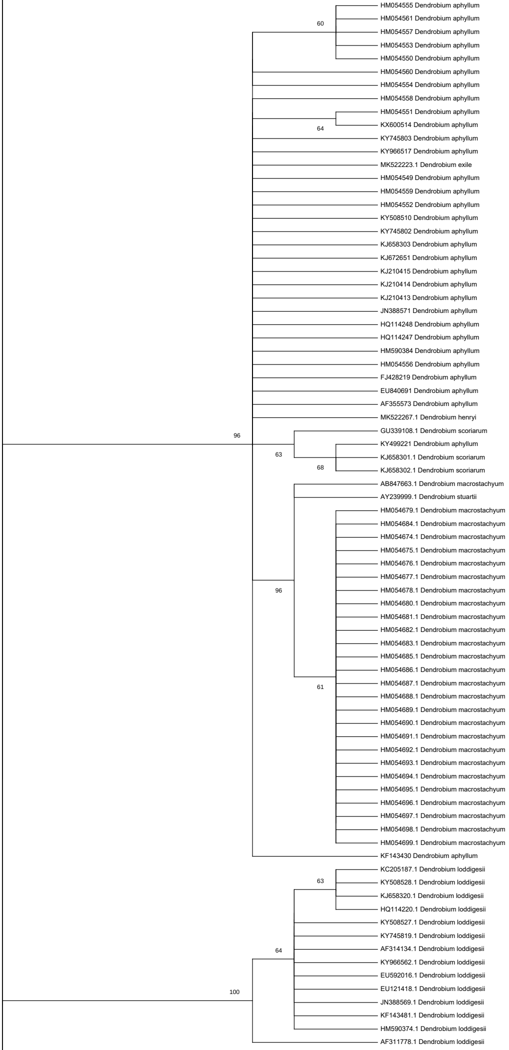
HM054555 Dendrobium aphyllum
HM054561 Dendrobium aphyllum
60
HM054557 Dendrobium aphyllum
HM054553 Dendrobium aphyllum
HM054550 Dendrobium aphyllum
HM054560 Dendrobium aphyllum
HM054554 Dendrobium aphyllum
HM054558 Dendrobium aphyllum
HM054551 Dendrobium aphyllum
KX600514 Dendrobium aphyllum
64
KY745803 Dendrobium aphyllum
KY966517 Dendrobium aphyllum
MK522223.1 Dendrobium exile
HM054549 Dendrobium aphyllum
HM054559 Dendrobium aphyllum
HM054552 Dendrobium aphyllum
KY508510 Dendrobium aphyllum
KY745802 Dendrobium aphyllum
KJ658303 Dendrobium aphyllum
KJ672651 Dendrobium aphyllum
KJ210415 Dendrobium aphyllum
KJ210414 Dendrobium aphyllum
KJ210413 Dendrobium aphyllum
JN388571 Dendrobium aphyllum
HQ114248 Dendrobium aphyllum
HQ114247 Dendrobium aphyllum
HM590384 Dendrobium aphyllum
HM054556 Dendrobium aphyllum
FJ428219 Dendrobium aphyllum
EU840691 Dendrobium aphyllum
AF355573 Dendrobium aphyllum
MK522267.1 Dendrobium henryi
96
GU339108.1 Dendrobium scoriarum
KY499221 Dendrobium aphyllum
63
KJ658301.1 Dendrobium scoriarum
68
KJ658302.1 Dendrobium scoriarum
AB847663.1 Dendrobium macrostachyum
AY239999.1 Dendrobium stuartii
HM054679.1 Dendrobium macrostachyum
HM054684.1 Dendrobium macrostachyum
HM054674.1 Dendrobium macrostachyum
HM054675.1 Dendrobium macrostachyum
HM054676.1 Dendrobium macrostachyum
HM054677.1 Dendrobium macrostachyum
96
HM054678.1 Dendrobium macrostachyum
HM054680.1 Dendrobium macrostachyum
HM054681.1 Dendrobium macrostachyum
HM054682.1 Dendrobium macrostachyum
HM054683.1 Dendrobium macrostachyum
HM054685.1 Dendrobium macrostachyum
HM054686.1 Dendrobium macrostachyum
HM054687.1 Dendrobium macrostachyum
61
HM054688.1 Dendrobium macrostachyum
HM054689.1 Dendrobium macrostachyum
HM054690.1 Dendrobium macrostachyum
HM054691.1 Dendrobium macrostachyum
HM054692.1 Dendrobium macrostachyum
HM054693.1 Dendrobium macrostachyum
HM054694.1 Dendrobium macrostachyum
HM054695.1 Dendrobium macrostachyum
HM054696.1 Dendrobium macrostachyum
HM054697.1 Dendrobium macrostachyum
HM054698.1 Dendrobium macrostachyum
HM054699.1 Dendrobium macrostachyum
KF143430 Dendrobium aphyllum
KC205187.1 Dendrobium loddigesii
63
KY508528.1 Dendrobium loddigesii
KJ658320.1 Dendrobium loddigesii
HQ114220.1 Dendrobium loddigesii
KY508527.1 Dendrobium loddigesii
KY745819.1 Dendrobium loddigesii
64
AF314134.1 Dendrobium loddigesii
KY966562.1 Dendrobium loddigesii
EU592016.1 Dendrobium loddigesii
100
EU121418.1 Dendrobium loddigesii
JN388569.1 Dendrobium loddigesii
KF143481.1 Dendrobium loddigesii
HM590374.1 Dendrobium loddigesii
AF311778.1 Dendrobium loddigesii

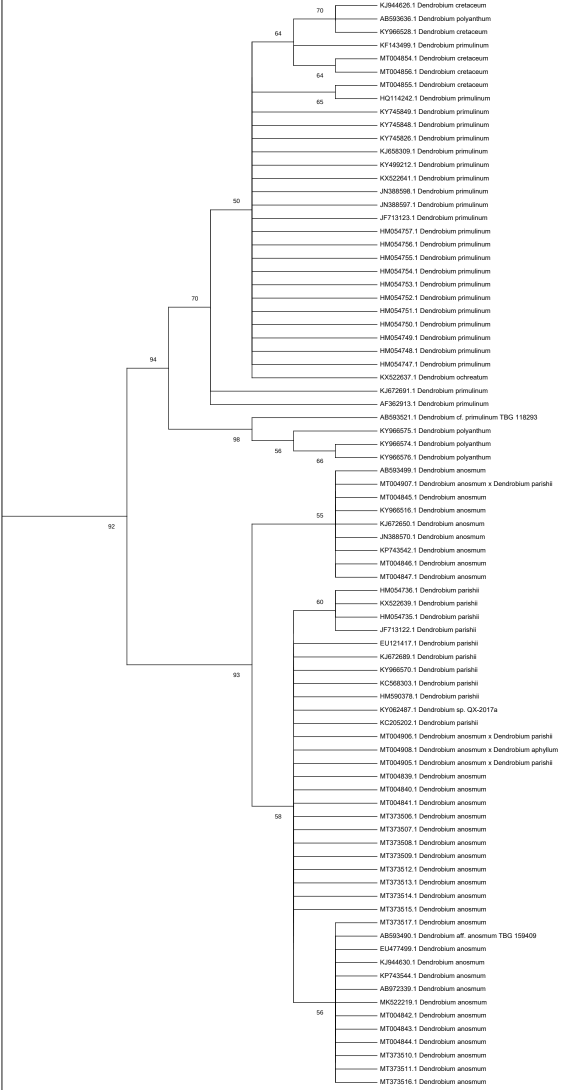
KJ944626.1 Dendrobium cretaceum
70
AB593636.1 Dendrobium polyanthum
64
KY966528.1 Dendrobium cretaceum
KF143499.1 Dendrobium primulinum
MT004854.1 Dendrobium cretaceum
MT004856.1 Dendrobium cretaceum
64
MT004855.1 Dendrobium cretaceum
HQ114242.1 Dendrobium primulinum
65
KY745849.1 Dendrobium primulinum
KY745848.1 Dendrobium primulinum
KY745826.1 Dendrobium primulinum
KJ658309.1 Dendrobium primulinum
KY499212.1 Dendrobium primulinum
KX522641.1 Dendrobium primulinum
JN388598.1 Dendrobium primulinum
50
JN388597.1 Dendrobium primulinum
JF713123.1 Dendrobium primulinum
HM054757.1 Dendrobium primulinum
HM054756.1 Dendrobium primulinum
HM054755.1 Dendrobium primulinum
HM054754.1 Dendrobium primulinum
HM054753.1 Dendrobium primulinum
70
HM054752.1 Dendrobium primulinum
HM054751.1 Dendrobium primulinum
HM054750.1 Dendrobium primulinum
HM054749.1 Dendrobium primulinum
HM054748.1 Dendrobium primulinum
94
HM054747.1 Dendrobium primulinum
KX522637.1 Dendrobium ochreatum
KJ672691.1 Dendrobium primulinum
AF362913.1 Dendrobium primulinum
AB593521.1 Dendrobium cf. primulinum TBG 118293
KY966575.1 Dendrobium polyanthum
98
KY966574.1 Dendrobium polyanthum
56
KY966576.1 Dendrobium polyanthum
66
AB593499.1 Dendrobium anosmum
MT004907.1 Dendrobium anosmum x Dendrobium parishii
MT004845.1 Dendrobium anosmum
KY966516.1 Dendrobium anosmum
55
KJ672650.1 Dendrobium anosmum
92
JN388570.1 Dendrobium anosmum
KP743542.1 Dendrobium anosmum
MT004846.1 Dendrobium anosmum
MT004847.1 Dendrobium anosmum
HM054736.1 Dendrobium parishii
60
KX522639.1 Dendrobium parishii
HM054735.1 Dendrobium parishii
JF713122.1 Dendrobium parishii
EU121417.1 Dendrobium parishii
KJ672689.1 Dendrobium parishii
KY966570.1 Dendrobium parishii
93
KC568303.1 Dendrobium parishii
HM590378.1 Dendrobium parishii
KY062487.1 Dendrobium sp. QX-2017a
KC205202.1 Dendrobium parishii
MT004906.1 Dendrobium anosmum x Dendrobium parishii
MT004908.1 Dendrobium anosmum x Dendrobium aphyllum
MT004905.1 Dendrobium anosmum x Dendrobium parishii
MT004839.1 Dendrobium anosmum
MT004840.1 Dendrobium anosmum
MT004841.1 Dendrobium anosmum
58
MT373506.1 Dendrobium anosmum
MT373507.1 Dendrobium anosmum
MT373508.1 Dendrobium anosmum
MT373509.1 Dendrobium anosmum
MT373512.1 Dendrobium anosmum
MT373513.1 Dendrobium anosmum
MT373514.1 Dendrobium anosmum
MT373515.1 Dendrobium anosmum
MT373517.1 Dendrobium anosmum
AB593490.1 Dendrobium aff. anosmum TBG 159409
EU477499.1 Dendrobium anosmum
KJ944630.1 Dendrobium anosmum
KP743544.1 Dendrobium anosmum
AB972339.1 Dendrobium anosmum
MK522219.1 Dendrobium anosmum
56
MT004842.1 Dendrobium anosmum
MT004843.1 Dendrobium anosmum
MT004844.1 Dendrobium anosmum
MT373510.1 Dendrobium anosmum
MT373511.1 Dendrobium anosmum
MT373516.1 Dendrobium anosmum

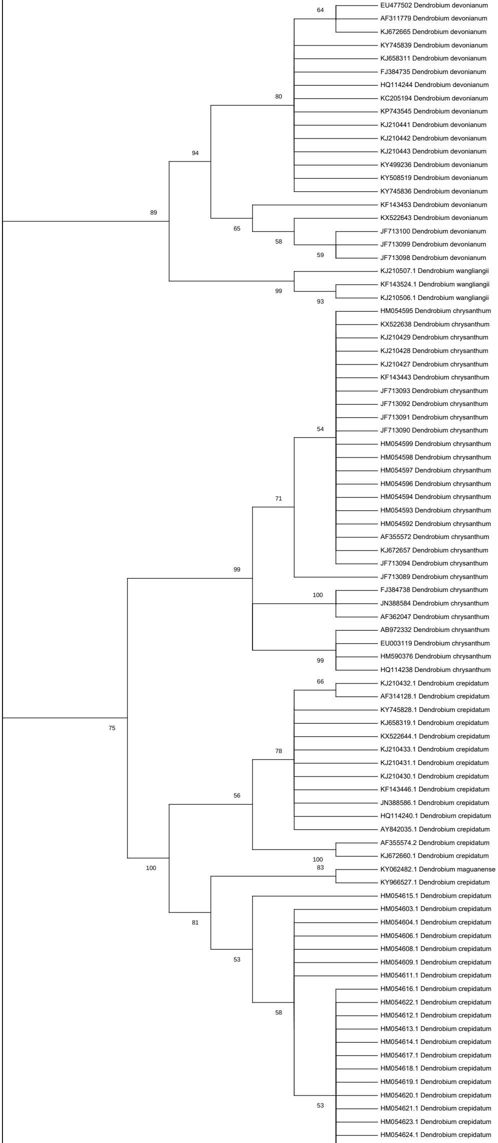

64
EU477502 Dendrobium devonianum
AF311779 Dendrobium devonianum
KJ672665 Dendrobium devonianum
KY745839 Dendrobium devonianum
KJ658311 Dendrobium devonianum
FJ384735 Dendrobium devonianum
HQ114244 Dendrobium devonianum
80
KC205194 Dendrobium devonianum
KP743545 Dendrobium devonianum
KJ210441 Dendrobium devonianum
KJ210442 Dendrobium devonianum
94
KJ210443 Dendrobium devonianum
KY499236 Dendrobium devonianum
KY508519 Dendrobium devonianum
KY745836 Dendrobium devonianum
KF143453 Dendrobium devonianum
89
KX522643 Dendrobium devonianum
65
JF713100 Dendrobium devonianum
58
JF713099 Dendrobium devonianum
59
JF713098 Dendrobium devonianum
KJ210507.1 Dendrobium wangliangii
KF143524.1 Dendrobium wangliangii
99
KJ210506.1 Dendrobium wangliangii
93
HM054595 Dendrobium chrysanthum
KX522638 Dendrobium chrysanthum
KJ210429 Dendrobium chrysanthum
KJ210428 Dendrobium chrysanthum
KJ210427 Dendrobium chrysanthum
KF143443 Dendrobium chrysanthum
JF713093 Dendrobium chrysanthum
JF713092 Dendrobium chrysanthum
JF713091 Dendrobium chrysanthum
54
JF713090 Dendrobium chrysanthum
HM054599 Dendrobium chrysanthum
HM054598 Dendrobium chrysanthum
HM054597 Dendrobium chrysanthum
HM054596 Dendrobium chrysanthum
71
HM054594 Dendrobium chrysanthum
HM054593 Dendrobium chrysanthum
HM054592 Dendrobium chrysanthum
AF355572 Dendrobium chrysanthum
KJ672657 Dendrobium chrysanthum
99
JF713094 Dendrobium chrysanthum
JF713089 Dendrobium chrysanthum
100
FJ384738 Dendrobium chrysanthum
JN388584 Dendrobium chrysanthum
AF362047 Dendrobium chrysanthum
AB972332 Dendrobium chrysanthum
EU003119 Dendrobium chrysanthum
99
HM590376 Dendrobium chrysanthum
HQ114238 Dendrobium chrysanthum
66
KJ210432.1 Dendrobium crepidatum
AF314128.1 Dendrobium crepidatum
KY745828.1 Dendrobium crepidatum
75
KJ658319.1 Dendrobium crepidatum
KX522644.1 Dendrobium crepidatum
78
KJ210433.1 Dendrobium crepidatum
KJ210431.1 Dendrobium crepidatum
KJ210430.1 Dendrobium crepidatum
56
KF143446.1 Dendrobium crepidatum
JN388586.1 Dendrobium crepidatum
HQ114240.1 Dendrobium crepidatum
AY842035.1 Dendrobium crepidatum
AF355574.2 Dendrobium crepidatum
100
KJ672660.1 Dendrobium crepidatum
100
83
KY062482.1 Dendrobium maguanense
KY966527.1 Dendrobium crepidatum
HM054615.1 Dendrobium crepidatum
HM054603.1 Dendrobium crepidatum
81
HM054604.1 Dendrobium crepidatum
HM054606.1 Dendrobium crepidatum
HM054608.1 Dendrobium crepidatum
53
HM054609.1 Dendrobium crepidatum
HM054611.1 Dendrobium crepidatum
HM054616.1 Dendrobium crepidatum
HM054622.1 Dendrobium crepidatum
58
HM054612.1 Dendrobium crepidatum
HM054613.1 Dendrobium crepidatum
HM054614.1 Dendrobium crepidatum
HM054617.1 Dendrobium crepidatum
HM054618.1 Dendrobium crepidatum
HM054619.1 Dendrobium crepidatum
HM054620.1 Dendrobium crepidatum
53
HM054621.1 Dendrobium crepidatum
HM054623.1 Dendrobium crepidatum
HM054624.1 Dendrobium crepidatum

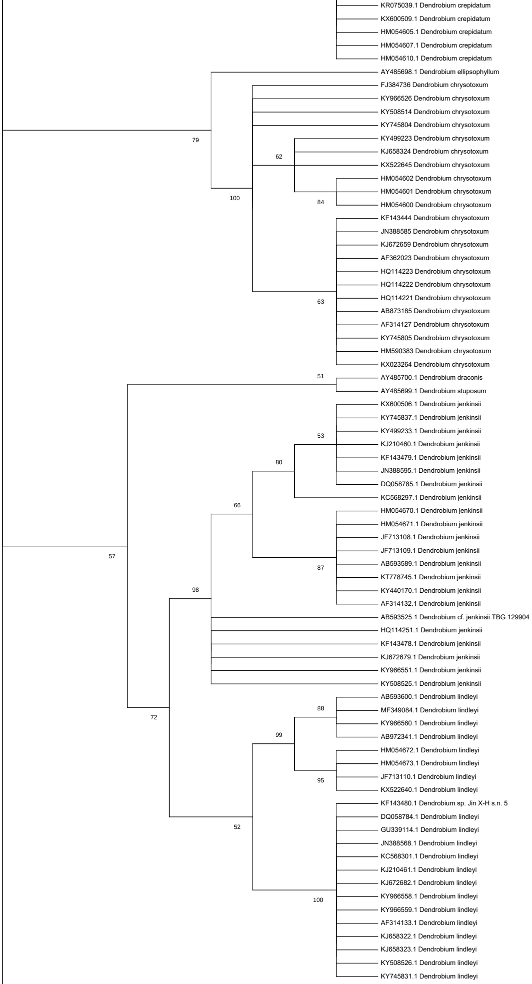
KR075039.1 Dendrobium crepidatum
KX600509.1 Dendrobium crepidatum
HM054605.1 Dendrobium crepidatum
HM054607.1 Dendrobium crepidatum
HM054610.1 Dendrobium crepidatum
AY485698.1 Dendrobium ellipsophyllum
FJ384736 Dendrobium chrysotoxum
KY966526 Dendrobium chrysotoxum
KY508514 Dendrobium chrysotoxum
KY745804 Dendrobium chrysotoxum
79
KY499223 Dendrobium chrysotoxum
KJ658324 Dendrobium chrysotoxum
62
KX522645 Dendrobium chrysotoxum
HM054602 Dendrobium chrysotoxum
HM054601 Dendrobium chrysotoxum
100
84
HM054600 Dendrobium chrysotoxum
KF143444 Dendrobium chrysotoxum
JN388585 Dendrobium chrysotoxum
KJ672659 Dendrobium chrysotoxum
AF362023 Dendrobium chrysotoxum
HQ114223 Dendrobium chrysotoxum
HQ114222 Dendrobium chrysotoxum
HQ114221 Dendrobium chrysotoxum
63
AB873185 Dendrobium chrysotoxum
AF314127 Dendrobium chrysotoxum
KY745805 Dendrobium chrysotoxum
HM590383 Dendrobium chrysotoxum
KX023264 Dendrobium chrysotoxum
51
AY485700.1 Dendrobium draconis
AY485699.1 Dendrobium stuposum
KX600506.1 Dendrobium jenkinsii
KY745837.1 Dendrobium jenkinsii
KY499233.1 Dendrobium jenkinsii
53
KJ210460.1 Dendrobium jenkinsii
80
KF143479.1 Dendrobium jenkinsii
JN388595.1 Dendrobium jenkinsii
DQ058785.1 Dendrobium jenkinsii
KC568297.1 Dendrobium jenkinsii
66
HM054670.1 Dendrobium jenkinsii
HM054671.1 Dendrobium jenkinsii
JF713108.1 Dendrobium jenkinsii
JF713109.1 Dendrobium jenkinsii
57
87
AB593589.1 Dendrobium jenkinsii
KT778745.1 Dendrobium jenkinsii
98
KY440170.1 Dendrobium jenkinsii
AF314132.1 Dendrobium jenkinsii
AB593525.1 Dendrobium cf. jenkinsii TBG 129904
HQ114251.1 Dendrobium jenkinsii
KF143478.1 Dendrobium jenkinsii
KJ672679.1 Dendrobium jenkinsii
KY966551.1 Dendrobium jenkinsii
KY508525.1 Dendrobium jenkinsii
AB593600.1 Dendrobium lindleyi
88
MF349084.1 Dendrobium lindleyi
72
KY966560.1 Dendrobium lindleyi
99
AB972341.1 Dendrobium lindleyi
HM054672.1 Dendrobium lindleyi
HM054673.1 Dendrobium lindleyi
95
JF713110.1 Dendrobium lindleyi
KX522640.1 Dendrobium lindleyi
KF143480.1 Dendrobium sp. Jin X-H s.n. 5
DQ058784.1 Dendrobium lindleyi
52
GU339114.1 Dendrobium lindleyi
JN388568.1 Dendrobium lindleyi
KC568301.1 Dendrobium lindleyi
KJ210461.1 Dendrobium lindleyi
KJ672682.1 Dendrobium lindleyi
100
KY966558.1 Dendrobium lindleyi
KY966559.1 Dendrobium lindleyi
AF314133.1 Dendrobium lindleyi
KJ658322.1 Dendrobium lindleyi
KJ658323.1 Dendrobium lindleyi
KY508526.1 Dendrobium lindleyi
KY745831.1 Dendrobium lindleyi

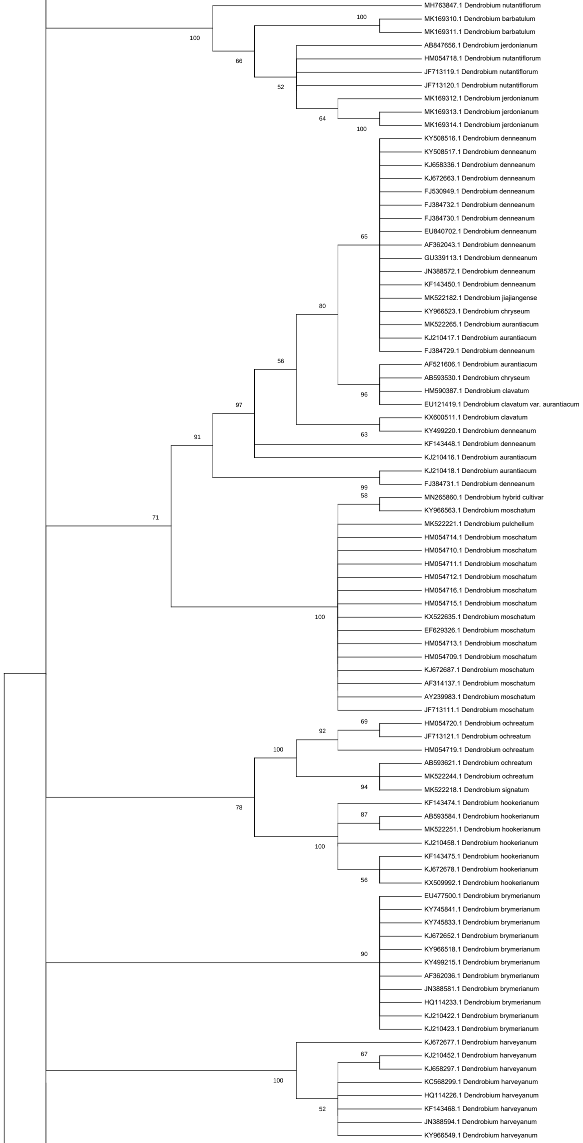
MH763847.1 Dendrobium nutantiflorum
100
MK169310.1 Dendrobium barbatulum
MK169311.1 Dendrobium barbatulum
100
AB847656.1 Dendrobium jerdonianum
HM054718.1 Dendrobium nutantiflorum
66
JF713119.1 Dendrobium nutantiflorum
JF713120.1 Dendrobium nutantiflorum
52
MK169312.1 Dendrobium jerdonianum
MK169313.1 Dendrobium jerdonianum
64
MK169314.1 Dendrobium jerdonianum
100
KY508516.1 Dendrobium denneanum
KY508517.1 Dendrobium denneanum
KJ658336.1 Dendrobium denneanum
KJ672663.1 Dendrobium denneanum
FJ530949.1 Dendrobium denneanum
FJ384732.1 Dendrobium denneanum
FJ384730.1 Dendrobium denneanum
EU840702.1 Dendrobium denneanum
65
AF362043.1 Dendrobium denneanum
GU339113.1 Dendrobium denneanum
JN388572.1 Dendrobium denneanum
KF143450.1 Dendrobium denneanum
MK522182.1 Dendrobium jiajiangense
80
KY966523.1 Dendrobium chryseum
MK522265.1 Dendrobium aurantiacum
KJ210417.1 Dendrobium aurantiacum
FJ384729.1 Dendrobium denneanum
56
AF521606.1 Dendrobium aurantiacum
AB593530.1 Dendrobium chryseum
HM590387.1 Dendrobium clavatum
96
97
EU121419.1 Dendrobium clavatum var. aurantiacum
KX600511.1 Dendrobium clavatum
KY499220.1 Dendrobium denneanum
63
91
KF143448.1 Dendrobium denneanum
KJ210416.1 Dendrobium aurantiacum
KJ210418.1 Dendrobium aurantiacum
FJ384731.1 Dendrobium denneanum
99
58
MN265860.1 Dendrobium hybrid cultivar
KY966563.1 Dendrobium moschatum
71
MK522221.1 Dendrobium pulchellum
HM054714.1 Dendrobium moschatum
HM054710.1 Dendrobium moschatum
HM054711.1 Dendrobium moschatum
HM054712.1 Dendrobium moschatum
HM054716.1 Dendrobium moschatum
HM054715.1 Dendrobium moschatum
100
KX522635.1 Dendrobium moschatum
EF629326.1 Dendrobium moschatum
HM054713.1 Dendrobium moschatum
HM054709.1 Dendrobium moschatum
KJ672687.1 Dendrobium moschatum
AF314137.1 Dendrobium moschatum
AY239983.1 Dendrobium moschatum
JF713111.1 Dendrobium moschatum
69
HM054720.1 Dendrobium ochreatum
92
JF713121.1 Dendrobium ochreatum
100
HM054719.1 Dendrobium ochreatum
AB593621.1 Dendrobium ochreatum
MK522244.1 Dendrobium ochreatum
94
MK522218.1 Dendrobium signatum
KF143474.1 Dendrobium hookerianum
78
87
AB593584.1 Dendrobium hookerianum
MK522251.1 Dendrobium hookerianum
KJ210458.1 Dendrobium hookerianum
100
KF143475.1 Dendrobium hookerianum
KJ672678.1 Dendrobium hookerianum
56
KX509992.1 Dendrobium hookerianum
EU477500.1 Dendrobium brymerianum
KY745841.1 Dendrobium brymerianum
KY745833.1 Dendrobium brymerianum
KJ672652.1 Dendrobium brymerianum
90
KY966518.1 Dendrobium brymerianum
KY499215.1 Dendrobium brymerianum
AF362036.1 Dendrobium brymerianum
JN388581.1 Dendrobium brymerianum
HQ114233.1 Dendrobium brymerianum
KJ210422.1 Dendrobium brymerianum
KJ210423.1 Dendrobium brymerianum
KJ672677.1 Dendrobium harveyanum
67
KJ210452.1 Dendrobium harveyanum
KJ658297.1 Dendrobium harveyanum
100
KC568299.1 Dendrobium harveyanum
HQ114226.1 Dendrobium harveyanum
52
KF143468.1 Dendrobium harveyanum
JN388594.1 Dendrobium harveyanum
KY966549.1 Dendrobium harveyanum

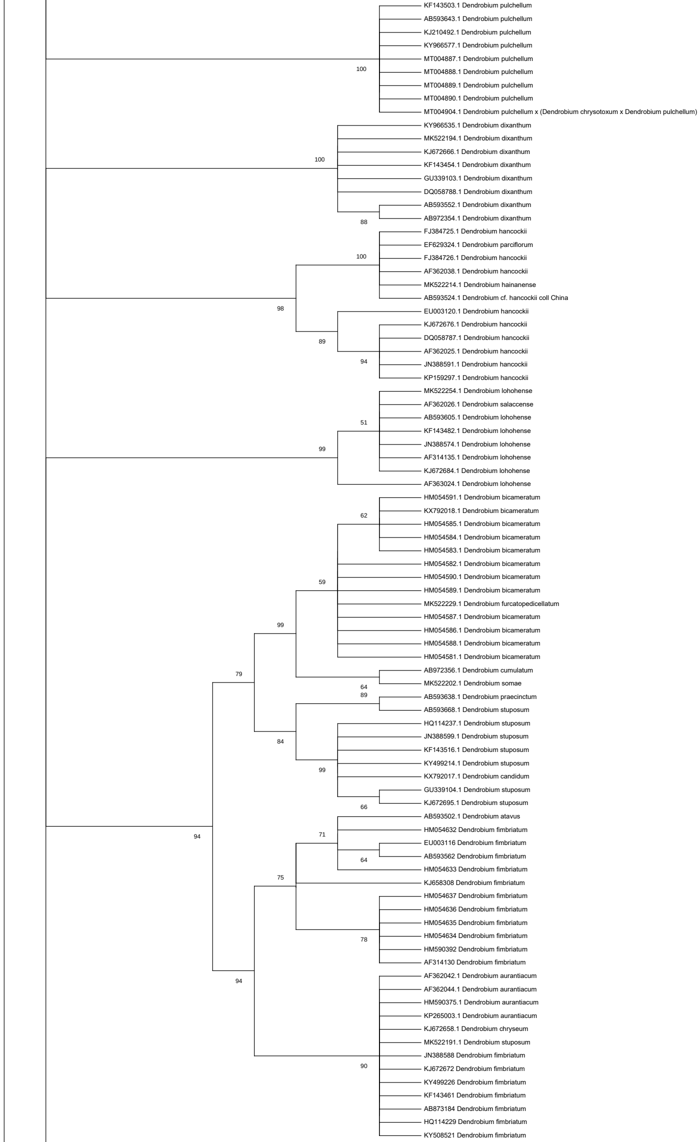

KF143503.1 Dendrobium pulchellum
AB593643.1 Dendrobium pulchellum
KJ210492.1 Dendrobium pulchellum
KY966577.1 Dendrobium pulchellum
MT004887.1 Dendrobium pulchellum
100
MT004888.1 Dendrobium pulchellum
MT004889.1 Dendrobium pulchellum
MT004890.1 Dendrobium pulchellum
MT004904.1 Dendrobium pulchellum x (Dendrobium chrysotoxum x Dendrobium pulchellum)
KY966535.1 Dendrobium dixanthum
MK522194.1 Dendrobium dixanthum
KJ672666.1 Dendrobium dixanthum
100
KF143454.1 Dendrobium dixanthum
GU339103.1 Dendrobium dixanthum
DQ058788.1 Dendrobium dixanthum
AB593552.1 Dendrobium dixanthum
AB972354.1 Dendrobium dixanthum
88
FJ384725.1 Dendrobium hancockii
EF629324.1 Dendrobium parciflorum
100
FJ384726.1 Dendrobium hancockii
AF362038.1 Dendrobium hancockii
MK522214.1 Dendrobium hainanense
AB593524.1 Dendrobium cf. hancockii coll China
98
EU003120.1 Dendrobium hancockii
KJ672676.1 Dendrobium hancockii
DQ058787.1 Dendrobium hancockii
89
AF362025.1 Dendrobium hancockii
94
JN388591.1 Dendrobium hancockii
KP159297.1 Dendrobium hancockii
MK522254.1 Dendrobium lohohense
AF362026.1 Dendrobium salaccense
AB593605.1 Dendrobium lohohense
51
KF143482.1 Dendrobium lohohense
JN388574.1 Dendrobium lohohense
99
AF314135.1 Dendrobium lohohense
KJ672684.1 Dendrobium lohohense
AF363024.1 Dendrobium lohohense
HM054591.1 Dendrobium bicameratum
KX792018.1 Dendrobium bicameratum
62
HM054585.1 Dendrobium bicameratum
HM054584.1 Dendrobium bicameratum
HM054583.1 Dendrobium bicameratum
HM054582.1 Dendrobium bicameratum
HM054590.1 Dendrobium bicameratum
59
HM054589.1 Dendrobium bicameratum
MK522229.1 Dendrobium furcatopedicellatum
HM054587.1 Dendrobium bicameratum
99
HM054586.1 Dendrobium bicameratum
HM054588.1 Dendrobium bicameratum
HM054581.1 Dendrobium bicameratum
AB972356.1 Dendrobium cumulatum
79
MK522202.1 Dendrobium somae
64
89
AB593638.1 Dendrobium praecinctum
AB593668.1 Dendrobium stuposum
HQ114237.1 Dendrobium stuposum
JN388599.1 Dendrobium stuposum
84
KF143516.1 Dendrobium stuposum
KY499214.1 Dendrobium stuposum
99
KX792017.1 Dendrobium candidum
GU339104.1 Dendrobium stuposum
KJ672695.1 Dendrobium stuposum
66
AB593502.1 Dendrobium atavus
HM054632 Dendrobium fimbriatum
94
71
EU003116 Dendrobium fimbriatum
AB593562 Dendrobium fimbriatum
64
HM054633 Dendrobium fimbriatum
75
KJ658308 Dendrobium fimbriatum
HM054637 Dendrobium fimbriatum
HM054636 Dendrobium fimbriatum
HM054635 Dendrobium fimbriatum
HM054634 Dendrobium fimbriatum
78
HM590392 Dendrobium fimbriatum
AF314130 Dendrobium fimbriatum
AF362042.1 Dendrobium aurantiacum
94
AF362044.1 Dendrobium aurantiacum
HM590375.1 Dendrobium aurantiacum
KP265003.1 Dendrobium aurantiacum
KJ672658.1 Dendrobium chryseum
MK522191.1 Dendrobium stuposum
JN388588 Dendrobium fimbriatum
90
KJ672672 Dendrobium fimbriatum
KY499226 Dendrobium fimbriatum
KF143461 Dendrobium fimbriatum
AB873184 Dendrobium fimbriatum
HQ114229 Dendrobium fimbriatum
KY508521 Dendrobium fimbriatum

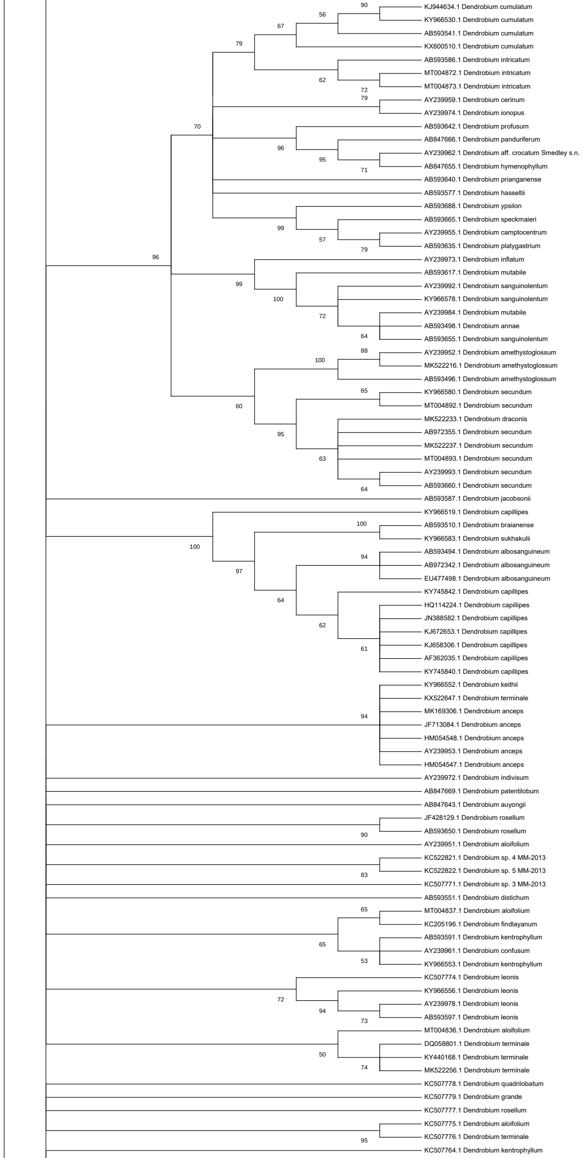
KJ944634.1 Dendrobium cumulatum
KY966530.1 Dendrobium cumulatum
AB593541.1 Dendrobium cumulatum
KX600510.1 Dendrobium cumulatum
AB593586.1 Dendrobium intricatum
MT004872.1 Dendrobium intricatum
MT004873.1 Dendrobium intricatum
AY239959.1 Dendrobium cerinum
AY239974.1 Dendrobium ionopus
AB593642.1 Dendrobium profusum
AB847666.1 Dendrobium panduriferum
AY239962.1 Dendrobium aff. crocatum Smedley s.n.
AB847655.1 Dendrobium hymenophyllum
AB593640.1 Dendrobium prianganense
AB593577.1 Dendrobium hasseltii
AB593688.1 Dendrobium ypsilon
AB593665.1 Dendrobium speckmaieri
AY239955.1 Dendrobium camptocentrum
AB593635.1 Dendrobium platygastrium
AY239973.1 Dendrobium inflatum
AB593617.1 Dendrobium mutabile
AY239992.1 Dendrobium sanguinolentum
KY966578.1 Dendrobium sanguinolentum
AY239984.1 Dendrobium mutabile
AB593498.1 Dendrobium annae
AB593655.1 Dendrobium sanguinolentum
AY239952.1 Dendrobium amethystoglossum
MK522216.1 Dendrobium amethystoglossum
AB593496.1 Dendrobium amethystoglossum
KY966580.1 Dendrobium secundum
MT004892.1 Dendrobium secundum
MK522233.1 Dendrobium draconis
AB972355.1 Dendrobium secundum
MK522237.1 Dendrobium secundum
MT004893.1 Dendrobium secundum
AY239993.1 Dendrobium secundum
AB593660.1 Dendrobium secundum
AB593587.1 Dendrobium jacobsonii
KY966519.1 Dendrobium capillipes
AB593510.1 Dendrobium braianense
KY966583.1 Dendrobium sukhakulii
AB593494.1 Dendrobium albosanguineum
AB972342.1 Dendrobium albosanguineum
EU477498.1 Dendrobium albosanguineum
KY745842.1 Dendrobium capillipes
HQ114224.1 Dendrobium capillipes
JN388582.1 Dendrobium capillipes
KJ672653.1 Dendrobium capillipes
KJ658306.1 Dendrobium capillipes
AF362035.1 Dendrobium capillipes
KY745840.1 Dendrobium capillipes
KY966552.1 Dendrobium keithii
KX522647.1 Dendrobium terminale
MK169306.1 Dendrobium anceps
JF713084.1 Dendrobium anceps
HM054548.1 Dendrobium anceps
AY239953.1 Dendrobium anceps
HM054547.1 Dendrobium anceps
AY239972.1 Dendrobium indivisum
AB847669.1 Dendrobium patentilobum
AB847643.1 Dendrobium auyongii
JF428129.1 Dendrobium rosellum
AB593650.1 Dendrobium rosellum
AY239951.1 Dendrobium aloifolium
KC522821.1 Dendrobium sp. 4 MM-2013
KC522822.1 Dendrobium sp. 5 MM-2013
KC507771.1 Dendrobium sp. 3 MM-2013
AB593551.1 Dendrobium distichum
MT004837.1 Dendrobium aloifolium
KC205196.1 Dendrobium findlayanum
AB593591.1 Dendrobium kentrophyllum
AY239961.1 Dendrobium confusum
KY966553.1 Dendrobium kentrophyllum
KC507774.1 Dendrobium leonis
KY966556.1 Dendrobium leonis
AY239978.1 Dendrobium leonis
AB593597.1 Dendrobium leonis
MT004836.1 Dendrobium aloifolium
DQ058801.1 Dendrobium terminale
KY440168.1 Dendrobium terminale
MK522256.1 Dendrobium terminale
KC507778.1 Dendrobium quadrilobatum
KC507779.1 Dendrobium grande
KC507777.1 Dendrobium rosellum
KC507775.1 Dendrobium aloifolium
KC507776.1 Dendrobium terminale
KC507764.1 Dendrobium kentrophyllum

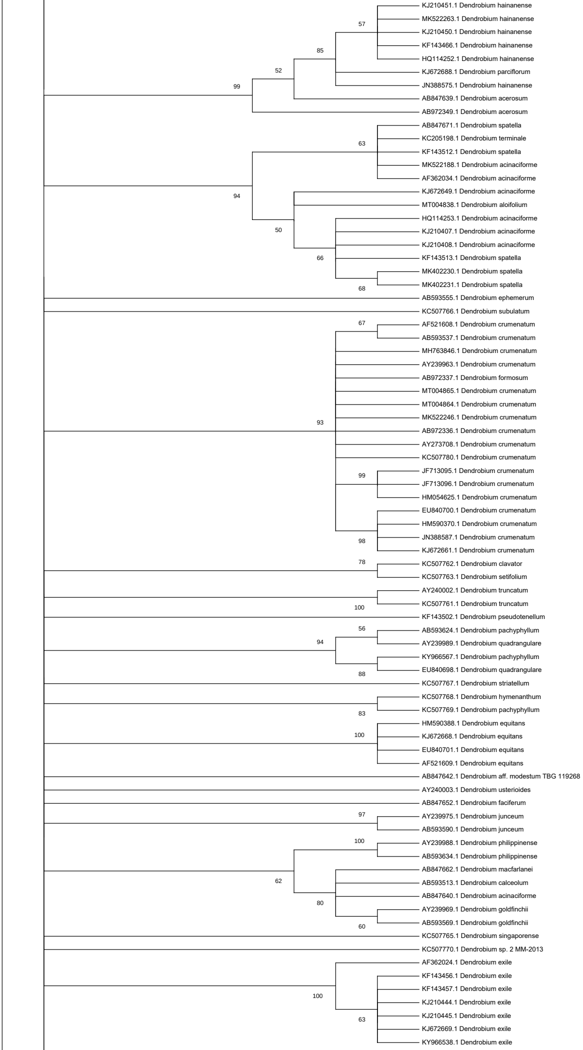
KJ210451.1 Dendrobium hainanense
MK522263.1 Dendrobium hainanense
KJ210450.1 Dendrobium hainanense
KF143466.1 Dendrobium hainanense
HQ114252.1 Dendrobium hainanense
KJ672688.1 Dendrobium parciflorum
JN388575.1 Dendrobium hainanense
AB847639.1 Dendrobium acerosum
AB972349.1 Dendrobium acerosum
AB847671.1 Dendrobium spatella
KC205198.1 Dendrobium terminale
KF143512.1 Dendrobium spatella
MK522188.1 Dendrobium acinaciforme
AF362034.1 Dendrobium acinaciforme
KJ672649.1 Dendrobium acinaciforme
MT004838.1 Dendrobium aloifolium
HQ114253.1 Dendrobium acinaciforme
KJ210407.1 Dendrobium acinaciforme
KJ210408.1 Dendrobium acinaciforme
KF143513.1 Dendrobium spatella
MK402230.1 Dendrobium spatella
MK402231.1 Dendrobium spatella
AB593555.1 Dendrobium ephemerum
KC507766.1 Dendrobium subulatum
AF521608.1 Dendrobium crumenatum
AB593537.1 Dendrobium crumenatum
MH763846.1 Dendrobium crumenatum
AY239963.1 Dendrobium crumenatum
AB972337.1 Dendrobium formosum
MT004865.1 Dendrobium crumenatum
MT004864.1 Dendrobium crumenatum
MK522246.1 Dendrobium crumenatum
AB972336.1 Dendrobium crumenatum
AY273708.1 Dendrobium crumenatum
KC507780.1 Dendrobium crumenatum
JF713095.1 Dendrobium crumenatum
JF713096.1 Dendrobium crumenatum
HM054625.1 Dendrobium crumenatum
EU840700.1 Dendrobium crumenatum
HM590370.1 Dendrobium crumenatum
JN388587.1 Dendrobium crumenatum
KJ672661.1 Dendrobium crumenatum
KC507762.1 Dendrobium clavator
KC507763.1 Dendrobium setifolium
AY240002.1 Dendrobium truncatum
KC507761.1 Dendrobium truncatum
KF143502.1 Dendrobium pseudotenellum
AB593624.1 Dendrobium pachyphyllum
AY239989.1 Dendrobium quadrangulare
KY966567.1 Dendrobium pachyphyllum
EU840698.1 Dendrobium quadrangulare
KC507767.1 Dendrobium striatellum
KC507768.1 Dendrobium hymenanthum
KC507769.1 Dendrobium pachyphyllum
HM590388.1 Dendrobium equitans
KJ672668.1 Dendrobium equitans
EU840701.1 Dendrobium equitans
AF521609.1 Dendrobium equitans
AB847642.1 Dendrobium aff. modestum TBG 119268
AY240003.1 Dendrobium usterioides
AB847652.1 Dendrobium faciferum
AY239975.1 Dendrobium junceum
AB593590.1 Dendrobium junceum
AY239988.1 Dendrobium philippinense
AB593634.1 Dendrobium philippinense
AB847662.1 Dendrobium macfarlanei
AB593513.1 Dendrobium calceolum
AB847640.1 Dendrobium acinaciforme
AY239969.1 Dendrobium goldfinchii
AB593569.1 Dendrobium goldfinchii
KC507765.1 Dendrobium singaporense
KC507770.1 Dendrobium sp. 2 MM-2013
AF362024.1 Dendrobium exile
KF143456.1 Dendrobium exile
KF143457.1 Dendrobium exile
KJ210444.1 Dendrobium exile
KJ210445.1 Dendrobium exile
KJ672669.1 Dendrobium exile
KY966538.1 Dendrobium exile

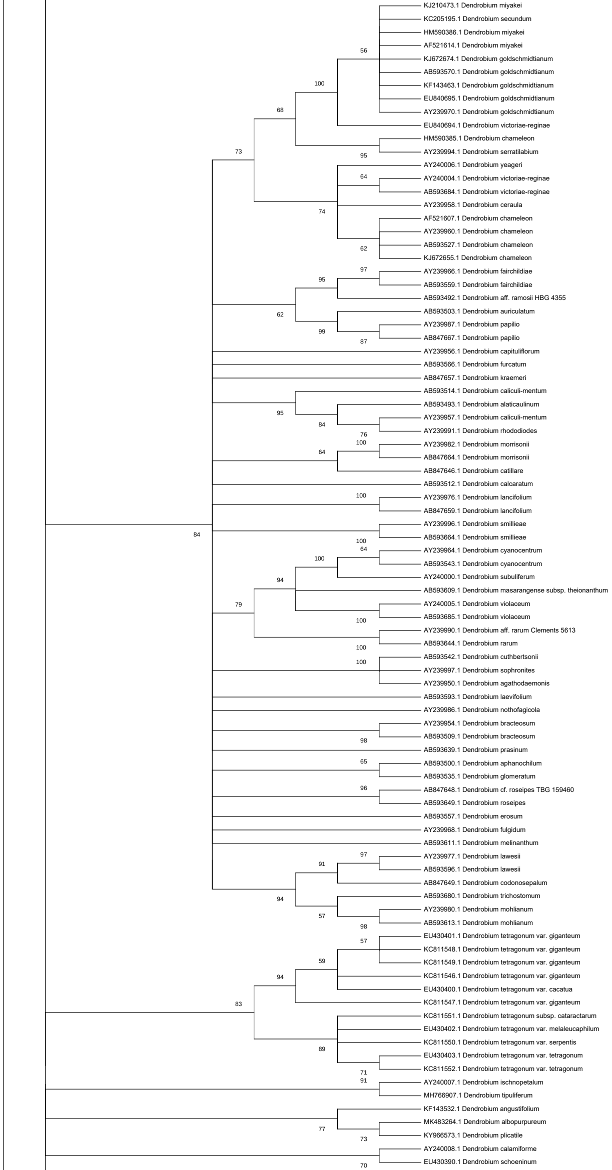
KJ210473.1 Dendrobium miyakei
KC205195.1 Dendrobium secundum
HM590386.1 Dendrobium miyakei
AF521614.1 Dendrobium miyakei
KJ672674.1 Dendrobium goldschmidtianum
AB593570.1 Dendrobium goldschmidtianum
KF143463.1 Dendrobium goldschmidtianum
EU840695.1 Dendrobium goldschmidtianum
AY239970.1 Dendrobium goldschmidtianum
EU840694.1 Dendrobium victoriae-reginae
HM590385.1 Dendrobium chameleon
AY239994.1 Dendrobium serratilabium
AY240006.1 Dendrobium yeageri
AY240004.1 Dendrobium victoriae-reginae
AB593684.1 Dendrobium victoriae-reginae
AY239958.1 Dendrobium ceraula
AF521607.1 Dendrobium chameleon
AY239960.1 Dendrobium chameleon
AB593527.1 Dendrobium chameleon
KJ672655.1 Dendrobium chameleon
AY239966.1 Dendrobium fairchildiae
AB593559.1 Dendrobium fairchildiae
AB593492.1 Dendrobium aff. ramosii HBG 4355
AB593503.1 Dendrobium auriculatum
AY239987.1 Dendrobium papilio
AB847667.1 Dendrobium papilio
AY239956.1 Dendrobium capituliflorum
AB593566.1 Dendrobium furcatum
AB847657.1 Dendrobium kraemeri
AB593514.1 Dendrobium caliculi-mentum
AB593493.1 Dendrobium alaticaulinum
AY239957.1 Dendrobium caliculi-mentum
AY239991.1 Dendrobium rhododiodes
AY239982.1 Dendrobium morrisonii
AB847664.1 Dendrobium morrisonii
AB847646.1 Dendrobium catillare
AB593512.1 Dendrobium calcaratum
AY239976.1 Dendrobium lancifolium
AB847659.1 Dendrobium lancifolium
AY239996.1 Dendrobium smillieae
AB593664.1 Dendrobium smillieae
AY239964.1 Dendrobium cyanocentrum
AB593543.1 Dendrobium cyanocentrum
AY240000.1 Dendrobium subuliferum
AB593609.1 Dendrobium masarangense subsp. theionanthum
AY240005.1 Dendrobium violaceum
AB593685.1 Dendrobium violaceum
AY239990.1 Dendrobium aff. rarum Clements 5613
AB593644.1 Dendrobium rarum
AB593542.1 Dendrobium cuthbertsonii
AY239997.1 Dendrobium sophronites
AY239950.1 Dendrobium agathodaemonis
AB593593.1 Dendrobium laevifolium
AY239986.1 Dendrobium nothofagicola
AY239954.1 Dendrobium bracteosum
AB593509.1 Dendrobium bracteosum
AB593639.1 Dendrobium prasinum
AB593500.1 Dendrobium aphanochilum
AB593535.1 Dendrobium glomeratum
AB847648.1 Dendrobium cf. roseipes TBG 159460
AB593649.1 Dendrobium roseipes
AB593557.1 Dendrobium erosum
AY239968.1 Dendrobium fulgidum
AB593611.1 Dendrobium melinanthum
AY239977.1 Dendrobium lawesii
AB593596.1 Dendrobium lawesii
AB847649.1 Dendrobium codonosepalum
AB593680.1 Dendrobium trichostomum
AY239980.1 Dendrobium mohlianum
AB593613.1 Dendrobium mohlianum
EU430401.1 Dendrobium tetragonum var. giganteum
KC811548.1 Dendrobium tetragonum var. giganteum
KC811549.1 Dendrobium tetragonum var. giganteum
KC811546.1 Dendrobium tetragonum var. giganteum
EU430400.1 Dendrobium tetragonum var. cacatua
KC811547.1 Dendrobium tetragonum var. giganteum
KC811551.1 Dendrobium tetragonum subsp. cataractarum
EU430402.1 Dendrobium tetragonum var. melaleucaphilum
KC811550.1 Dendrobium tetragonum var. serpentis
EU430403.1 Dendrobium tetragonum var. tetragonum
KC811552.1 Dendrobium tetragonum var. tetragonum
AY240007.1 Dendrobium ischnopetalum
MH766907.1 Dendrobium tipuliferum
KF143532.1 Dendrobium angustifolium
MK483264.1 Dendrobium albopurpureum
KY966573.1 Dendrobium plicatile
AY240008.1 Dendrobium calamiforme
EU430390.1 Dendrobium schoeninum

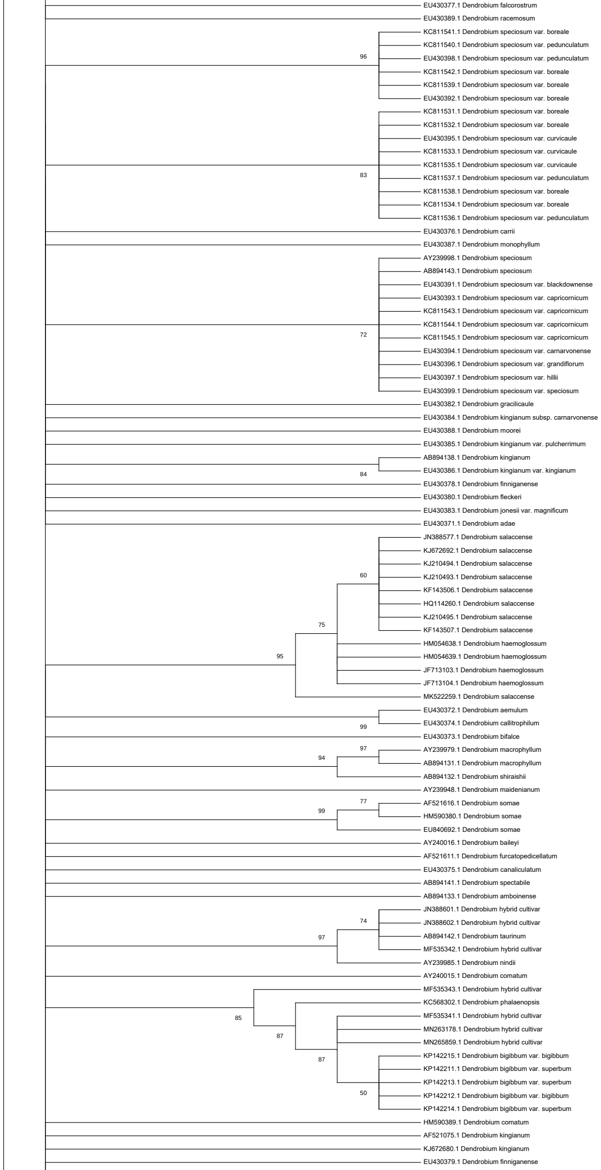
EU430377.1 Dendrobium falcorostrum
EU430389.1 Dendrobium racemosum
KC811541.1 Dendrobium speciosum var. boreale
KC811540.1 Dendrobium speciosum var. pedunculatum
EU430398.1 Dendrobium speciosum var. pedunculatum
KC811542.1 Dendrobium speciosum var. boreale
KC811539.1 Dendrobium speciosum var. boreale
EU430392.1 Dendrobium speciosum var. boreale
KC811531.1 Dendrobium speciosum var. boreale
KC811532.1 Dendrobium speciosum var. boreale
EU430395.1 Dendrobium speciosum var. curvicaule
KC811533.1 Dendrobium speciosum var. curvicaule
KC811535.1 Dendrobium speciosum var. curvicaule
KC811537.1 Dendrobium speciosum var. pedunculatum
KC811538.1 Dendrobium speciosum var. boreale
KC811534.1 Dendrobium speciosum var. boreale
KC811536.1 Dendrobium speciosum var. pedunculatum
EU430376.1 Dendrobium carrii
EU430387.1 Dendrobium monophyllum
AY239998.1 Dendrobium speciosum
AB894143.1 Dendrobium speciosum
EU430391.1 Dendrobium speciosum var. blackdownense
EU430393.1 Dendrobium speciosum var. capricornicum
KC811543.1 Dendrobium speciosum var. capricornicum
KC811544.1 Dendrobium speciosum var. capricornicum
KC811545.1 Dendrobium speciosum var. capricornicum
EU430394.1 Dendrobium speciosum var. carnarvonense
EU430396.1 Dendrobium speciosum var. grandiflorum
EU430397.1 Dendrobium speciosum var. hillii
EU430399.1 Dendrobium speciosum var. speciosum
EU430382.1 Dendrobium gracilicaule
EU430384.1 Dendrobium kingianum subsp. carnarvonense
EU430388.1 Dendrobium moorei
EU430385.1 Dendrobium kingianum var. pulcherrimum
AB894138.1 Dendrobium kingianum
EU430386.1 Dendrobium kingianum var. kingianum
EU430378.1 Dendrobium finniganense
EU430380.1 Dendrobium fleckeri
EU430383.1 Dendrobium jonesii var. magnificum
EU430371.1 Dendrobium adae
JN388577.1 Dendrobium salaccense
KJ672692.1 Dendrobium salaccense
KJ210494.1 Dendrobium salaccense
KJ210493.1 Dendrobium salaccense
KF143506.1 Dendrobium salaccense
HQ114260.1 Dendrobium salaccense
KJ210495.1 Dendrobium salaccense
KF143507.1 Dendrobium salaccense
HM054638.1 Dendrobium haemoglossum
HM054639.1 Dendrobium haemoglossum
JF713103.1 Dendrobium haemoglossum
JF713104.1 Dendrobium haemoglossum
MK522259.1 Dendrobium salaccense
EU430372.1 Dendrobium aemulum
EU430374.1 Dendrobium callitrophilum
EU430373.1 Dendrobium bifalce
AY239979.1 Dendrobium macrophyllum
AB894131.1 Dendrobium macrophyllum
AB894132.1 Dendrobium shiraishii
AY239948.1 Dendrobium maidenianum
AF521616.1 Dendrobium somae
HM590380.1 Dendrobium somae
EU840692.1 Dendrobium somae
AY240016.1 Dendrobium baileyi
AF521611.1 Dendrobium furcatopedicellatum
EU430375.1 Dendrobium canaliculatum
AB894141.1 Dendrobium spectabile
AB894133.1 Dendrobium amboinense
JN388601.1 Dendrobium hybrid cultivar
JN388602.1 Dendrobium hybrid cultivar
AB894142.1 Dendrobium taurinum
MF535342.1 Dendrobium hybrid cultivar
AY239985.1 Dendrobium nindii
AY240015.1 Dendrobium comatum
MF535343.1 Dendrobium hybrid cultivar
KC568302.1 Dendrobium phalaenopsis
MF535341.1 Dendrobium hybrid cultivar
MN263178.1 Dendrobium hybrid cultivar
MN265859.1 Dendrobium hybrid cultivar
KP142215.1 Dendrobium bigibbum var. bigibbum
KP142211.1 Dendrobium bigibbum var. superbum
KP142213.1 Dendrobium bigibbum var. superbum
KP142212.1 Dendrobium bigibbum var. bigibbum
KP142214.1 Dendrobium bigibbum var. superbum
HM590389.1 Dendrobium comatum
AF521075.1 Dendrobium kingianum
KJ672680.1 Dendrobium kingianum
EU430379.1 Dendrobium finniganense

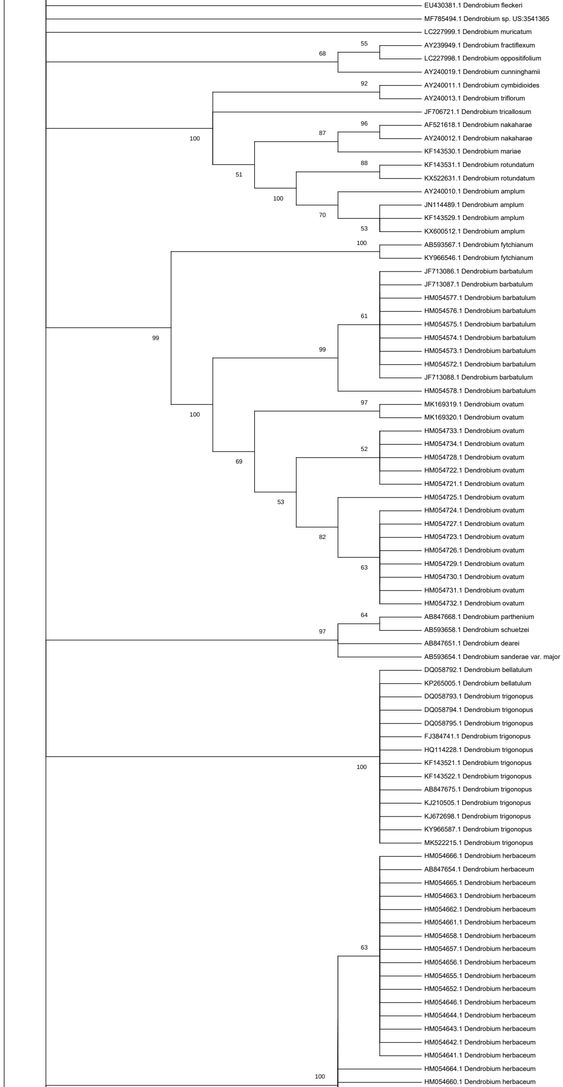

EU430381.1 Dendrobium fleckeri
MF785494.1 Dendrobium sp. US:3541365
LC227999.1 Dendrobium muricatum
55
AY239949.1 Dendrobium fractiflexum
68
LC227998.1 Dendrobium oppositifolium
AY240019.1 Dendrobium cunninghamii
92
AY240011.1 Dendrobium cymbidioides
AY240013.1 Dendrobium triflorum
JF706721.1 Dendrobium tricallosum
96
AF521618.1 Dendrobium nakaharae
87
100
AY240012.1 Dendrobium nakaharae
KF143530.1 Dendrobium mariae
88
KF143531.1 Dendrobium rotundatum
51
KX522631.1 Dendrobium rotundatum
AY240010.1 Dendrobium amplum
100
JN114489.1 Dendrobium amplum
70
KF143529.1 Dendrobium amplum
53
KX600512.1 Dendrobium amplum
100
AB593567.1 Dendrobium fytchianum
KY966546.1 Dendrobium fytchianum
JF713086.1 Dendrobium barbatulum
JF713087.1 Dendrobium barbatulum
HM054577.1 Dendrobium barbatulum
HM054576.1 Dendrobium barbatulum
61
HM054575.1 Dendrobium barbatulum
99
HM054574.1 Dendrobium barbatulum
HM054573.1 Dendrobium barbatulum
99
HM054572.1 Dendrobium barbatulum
JF713088.1 Dendrobium barbatulum
HM054578.1 Dendrobium barbatulum
97
MK169319.1 Dendrobium ovatum
100
MK169320.1 Dendrobium ovatum
HM054733.1 Dendrobium ovatum
HM054734.1 Dendrobium ovatum
52
HM054728.1 Dendrobium ovatum
69
HM054722.1 Dendrobium ovatum
HM054721.1 Dendrobium ovatum
HM054725.1 Dendrobium ovatum
53
HM054724.1 Dendrobium ovatum
HM054727.1 Dendrobium ovatum
82
HM054723.1 Dendrobium ovatum
HM054726.1 Dendrobium ovatum
63
HM054729.1 Dendrobium ovatum
HM054730.1 Dendrobium ovatum
HM054731.1 Dendrobium ovatum
HM054732.1 Dendrobium ovatum
64
AB847668.1 Dendrobium parthenium
97
AB593658.1 Dendrobium schuetzei
AB847651.1 Dendrobium dearei
AB593654.1 Dendrobium sanderae var. major
DQ058792.1 Dendrobium bellatulum
KP265005.1 Dendrobium bellatulum
DQ058793.1 Dendrobium trigonopus
DQ058794.1 Dendrobium trigonopus
DQ058795.1 Dendrobium trigonopus
FJ384741.1 Dendrobium trigonopus
HQ114228.1 Dendrobium trigonopus
100
KF143521.1 Dendrobium trigonopus
KF143522.1 Dendrobium trigonopus
AB847675.1 Dendrobium trigonopus
KJ210505.1 Dendrobium trigonopus
KJ672698.1 Dendrobium trigonopus
KY966587.1 Dendrobium trigonopus
MK522215.1 Dendrobium trigonopus
HM054666.1 Dendrobium herbaceum
AB847654.1 Dendrobium herbaceum
HM054665.1 Dendrobium herbaceum
HM054663.1 Dendrobium herbaceum
HM054662.1 Dendrobium herbaceum
HM054661.1 Dendrobium herbaceum
HM054658.1 Dendrobium herbaceum
63
HM054657.1 Dendrobium herbaceum
HM054656.1 Dendrobium herbaceum
HM054655.1 Dendrobium herbaceum
HM054652.1 Dendrobium herbaceum
HM054646.1 Dendrobium herbaceum
HM054644.1 Dendrobium herbaceum
HM054643.1 Dendrobium herbaceum
HM054642.1 Dendrobium herbaceum
HM054641.1 Dendrobium herbaceum
HM054664.1 Dendrobium herbaceum
100
HM054660.1 Dendrobium herbaceum

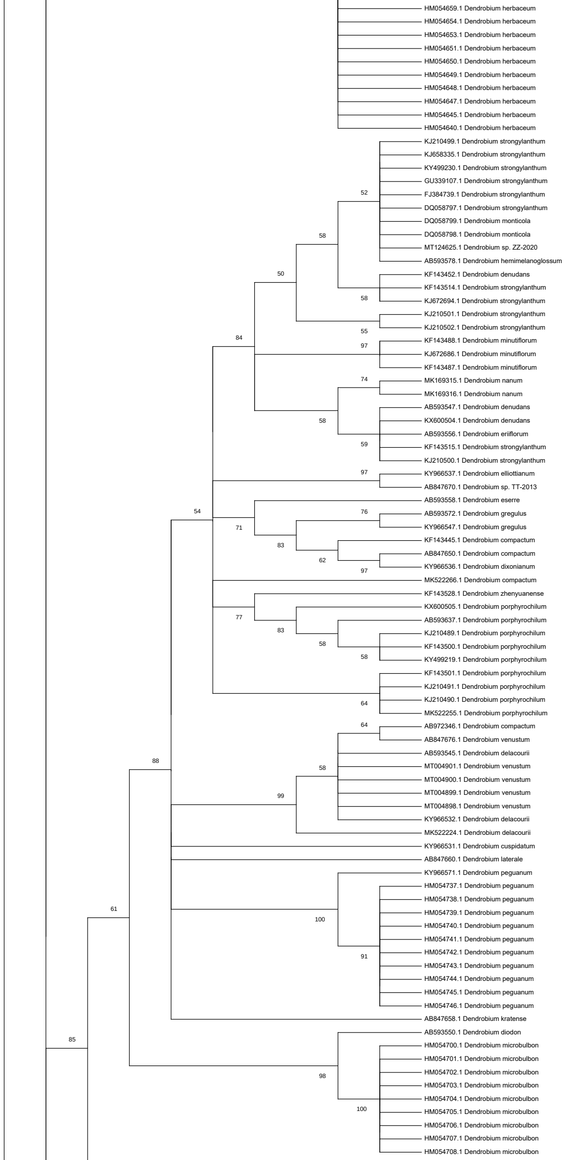
HM054659.1 Dendrobium herbaceum
HM054654.1 Dendrobium herbaceum
HM054653.1 Dendrobium herbaceum
HM054651.1 Dendrobium herbaceum
HM054650.1 Dendrobium herbaceum
HM054649.1 Dendrobium herbaceum
HM054648.1 Dendrobium herbaceum
HM054647.1 Dendrobium herbaceum
HM054645.1 Dendrobium herbaceum
HM054640.1 Dendrobium herbaceum
KJ210499.1 Dendrobium strongylanthum
KJ658335.1 Dendrobium strongylanthum
KY499230.1 Dendrobium strongylanthum
GU339107.1 Dendrobium strongylanthum
FJ384739.1 Dendrobium strongylanthum
DQ058797.1 Dendrobium strongylanthum
DQ058799.1 Dendrobium monticola
DQ058798.1 Dendrobium monticola
MT124625.1 Dendrobium sp. ZZ-2020
AB593578.1 Dendrobium hemimelanoglossum
KF143452.1 Dendrobium denudans
KF143514.1 Dendrobium strongylanthum
KJ672694.1 Dendrobium strongylanthum
KJ210501.1 Dendrobium strongylanthum
KJ210502.1 Dendrobium strongylanthum
KF143488.1 Dendrobium minutiflorum
KJ672686.1 Dendrobium minutiflorum
KF143487.1 Dendrobium minutiflorum
MK169315.1 Dendrobium nanum
MK169316.1 Dendrobium nanum
AB593547.1 Dendrobium denudans
KX600504.1 Dendrobium denudans
AB593556.1 Dendrobium eriiflorum
KF143515.1 Dendrobium strongylanthum
KJ210500.1 Dendrobium strongylanthum
KY966537.1 Dendrobium elliottianum
AB847670.1 Dendrobium sp. TT-2013
AB593558.1 Dendrobium eserre
AB593572.1 Dendrobium gregulus
KY966547.1 Dendrobium gregulus
KF143445.1 Dendrobium compactum
AB847650.1 Dendrobium compactum
KY966536.1 Dendrobium dixonianum
MK522266.1 Dendrobium compactum
KF143528.1 Dendrobium zhenyuanense
KX600505.1 Dendrobium porphyrochilum
AB593637.1 Dendrobium porphyrochilum
KJ210489.1 Dendrobium porphyrochilum
KF143500.1 Dendrobium porphyrochilum
KY499219.1 Dendrobium porphyrochilum
KF143501.1 Dendrobium porphyrochilum
KJ210491.1 Dendrobium porphyrochilum
KJ210490.1 Dendrobium porphyrochilum
MK522255.1 Dendrobium porphyrochilum
AB972346.1 Dendrobium compactum
AB847676.1 Dendrobium venustum
AB593545.1 Dendrobium delacourii
MT004901.1 Dendrobium venustum
MT004900.1 Dendrobium venustum
MT004899.1 Dendrobium venustum
MT004898.1 Dendrobium venustum
KY966532.1 Dendrobium delacourii
MK522224.1 Dendrobium delacourii
KY966531.1 Dendrobium cuspidatum
AB847660.1 Dendrobium laterale
KY966571.1 Dendrobium peguanum
HM054737.1 Dendrobium peguanum
HM054738.1 Dendrobium peguanum
HM054739.1 Dendrobium peguanum
HM054740.1 Dendrobium peguanum
HM054741.1 Dendrobium peguanum
HM054742.1 Dendrobium peguanum
HM054743.1 Dendrobium peguanum
HM054744.1 Dendrobium peguanum
HM054745.1 Dendrobium peguanum
HM054746.1 Dendrobium peguanum
AB847658.1 Dendrobium kratense
AB593550.1 Dendrobium diodon
HM054700.1 Dendrobium microbulbon
HM054701.1 Dendrobium microbulbon
HM054702.1 Dendrobium microbulbon
HM054703.1 Dendrobium microbulbon
HM054704.1 Dendrobium microbulbon
HM054705.1 Dendrobium microbulbon
HM054706.1 Dendrobium microbulbon
HM054707.1 Dendrobium microbulbon
HM054708.1 Dendrobium microbulbon

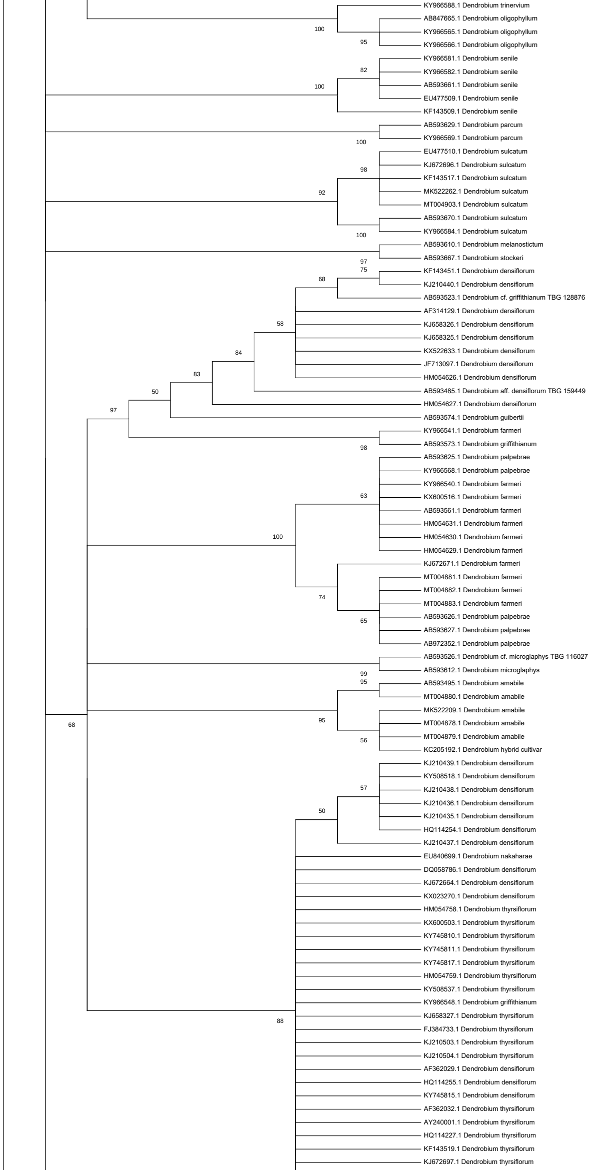
KY966588.1 Dendrobium trinervium
AB847665.1 Dendrobium oligophyllum
KY966565.1 Dendrobium oligophyllum
KY966566.1 Dendrobium oligophyllum
KY966581.1 Dendrobium senile
KY966582.1 Dendrobium senile
AB593661.1 Dendrobium senile
EU477509.1 Dendrobium senile
KF143509.1 Dendrobium senile
AB593629.1 Dendrobium parcum
KY966569.1 Dendrobium parcum
EU477510.1 Dendrobium sulcatum
KJ672696.1 Dendrobium sulcatum
KF143517.1 Dendrobium sulcatum
MK522262.1 Dendrobium sulcatum
MT004903.1 Dendrobium sulcatum
AB593670.1 Dendrobium sulcatum
KY966584.1 Dendrobium sulcatum
AB593610.1 Dendrobium melanostictum
AB593667.1 Dendrobium stockeri
KF143451.1 Dendrobium densiflorum
KJ210440.1 Dendrobium densiflorum
AB593523.1 Dendrobium cf. griffithianum TBG 128876
AF314129.1 Dendrobium densiflorum
KJ658326.1 Dendrobium densiflorum
KJ658325.1 Dendrobium densiflorum
KX522633.1 Dendrobium densiflorum
JF713097.1 Dendrobium densiflorum
HM054626.1 Dendrobium densiflorum
AB593485.1 Dendrobium aff. densiflorum TBG 159449
HM054627.1 Dendrobium densiflorum
AB593574.1 Dendrobium guibertii
KY966541.1 Dendrobium farmeri
AB593573.1 Dendrobium griffithianum
AB593625.1 Dendrobium palpebrae
KY966568.1 Dendrobium palpebrae
KY966540.1 Dendrobium farmeri
KX600516.1 Dendrobium farmeri
AB593561.1 Dendrobium farmeri
HM054631.1 Dendrobium farmeri
HM054630.1 Dendrobium farmeri
HM054629.1 Dendrobium farmeri
KJ672671.1 Dendrobium farmeri
MT004881.1 Dendrobium farmeri
MT004882.1 Dendrobium farmeri
MT004883.1 Dendrobium farmeri
AB593626.1 Dendrobium palpebrae
AB593627.1 Dendrobium palpebrae
AB972352.1 Dendrobium palpebrae
AB593526.1 Dendrobium cf. microglaphys TBG 116027
AB593612.1 Dendrobium microglaphys
AB593495.1 Dendrobium amabile
MT004880.1 Dendrobium amabile
MK522209.1 Dendrobium amabile
MT004878.1 Dendrobium amabile
MT004879.1 Dendrobium amabile
KC205192.1 Dendrobium hybrid cultivar
KJ210439.1 Dendrobium densiflorum
KY508518.1 Dendrobium densiflorum
KJ210438.1 Dendrobium densiflorum
KJ210436.1 Dendrobium densiflorum
KJ210435.1 Dendrobium densiflorum
HQ114254.1 Dendrobium densiflorum
KJ210437.1 Dendrobium densiflorum
EU840699.1 Dendrobium nakaharae
DQ058786.1 Dendrobium densiflorum
KJ672664.1 Dendrobium densiflorum
KX023270.1 Dendrobium densiflorum
HM054758.1 Dendrobium thyrsiflorum
KX600503.1 Dendrobium thyrsiflorum
KY745810.1 Dendrobium thyrsiflorum
KY745811.1 Dendrobium thyrsiflorum
KY745817.1 Dendrobium thyrsiflorum
HM054759.1 Dendrobium thyrsiflorum
KY508537.1 Dendrobium thyrsiflorum
KY966548.1 Dendrobium griffithianum
KJ658327.1 Dendrobium thyrsiflorum
FJ384733.1 Dendrobium thyrsiflorum
KJ210503.1 Dendrobium thyrsiflorum
KJ210504.1 Dendrobium thyrsiflorum
AF362029.1 Dendrobium densiflorum
HQ114255.1 Dendrobium densiflorum
KY745815.1 Dendrobium densiflorum
AF362032.1 Dendrobium thyrsiflorum
AY240001.1 Dendrobium thyrsiflorum
HQ114227.1 Dendrobium thyrsiflorum
KF143519.1 Dendrobium thyrsiflorum
KJ672697.1 Dendrobium thyrsiflorum

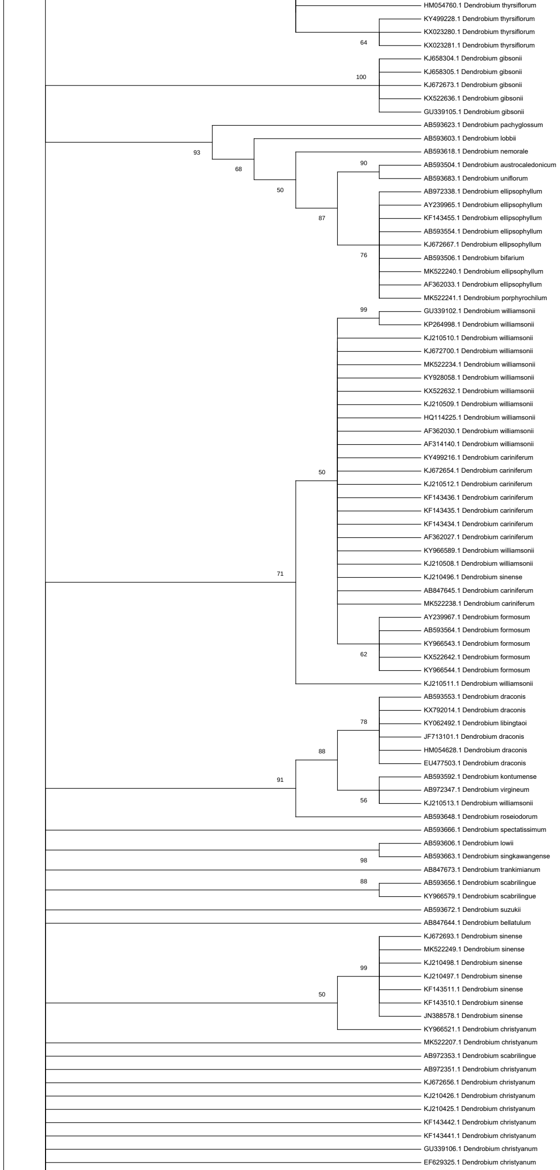

HM054760.1 Dendrobium thyrsiflorum
KY499228.1 Dendrobium thyrsiflorum
KX023280.1 Dendrobium thyrsiflorum
64
KX023281.1 Dendrobium thyrsiflorum
KJ658304.1 Dendrobium gibsonii
KJ658305.1 Dendrobium gibsonii
100
KJ672673.1 Dendrobium gibsonii
KX522636.1 Dendrobium gibsonii
GU339105.1 Dendrobium gibsonii
AB593623.1 Dendrobium pachyglossum
AB593603.1 Dendrobium lobbii
93
AB593618.1 Dendrobium nemorale
68
90
AB593504.1 Dendrobium austrocaledonicum
AB593683.1 Dendrobium uniflorum
50
AB972338.1 Dendrobium ellipsophyllum
AY239965.1 Dendrobium ellipsophyllum
87
KF143455.1 Dendrobium ellipsophyllum
AB593554.1 Dendrobium ellipsophyllum
KJ672667.1 Dendrobium ellipsophyllum
76
AB593506.1 Dendrobium bifarium
MK522240.1 Dendrobium ellipsophyllum
AF362033.1 Dendrobium ellipsophyllum
MK522241.1 Dendrobium porphyrochilum
99
GU339102.1 Dendrobium williamsonii
KP264998.1 Dendrobium williamsonii
KJ210510.1 Dendrobium williamsonii
KJ672700.1 Dendrobium williamsonii
MK522234.1 Dendrobium williamsonii
KY928058.1 Dendrobium williamsonii
KX522632.1 Dendrobium williamsonii
KJ210509.1 Dendrobium williamsonii
HQ114225.1 Dendrobium williamsonii
AF362030.1 Dendrobium williamsonii
AF314140.1 Dendrobium williamsonii
KY499216.1 Dendrobium cariniferum
50
KJ672654.1 Dendrobium cariniferum
KJ210512.1 Dendrobium cariniferum
KF143436.1 Dendrobium cariniferum
KF143435.1 Dendrobium cariniferum
KF143434.1 Dendrobium cariniferum
AF362027.1 Dendrobium cariniferum
KY966589.1 Dendrobium williamsonii
KJ210508.1 Dendrobium williamsonii
71
KJ210496.1 Dendrobium sinense
AB847645.1 Dendrobium cariniferum
MK522238.1 Dendrobium cariniferum
AY239967.1 Dendrobium formosum
AB593564.1 Dendrobium formosum
KY966543.1 Dendrobium formosum
62
KX522642.1 Dendrobium formosum
KY966544.1 Dendrobium formosum
KJ210511.1 Dendrobium williamsonii
AB593553.1 Dendrobium draconis
KX792014.1 Dendrobium draconis
78
KY062492.1 Dendrobium libingtaoi
JF713101.1 Dendrobium draconis
88
HM054628.1 Dendrobium draconis
EU477503.1 Dendrobium draconis
91
AB593592.1 Dendrobium kontumense
AB972347.1 Dendrobium virgineum
56
KJ210513.1 Dendrobium williamsonii
AB593648.1 Dendrobium roseiodorum
AB593666.1 Dendrobium spectatissimum
AB593606.1 Dendrobium lowii
AB593663.1 Dendrobium singkawangense
98
AB847673.1 Dendrobium trankimianum
88
AB593656.1 Dendrobium scabrilingue
KY966579.1 Dendrobium scabrilingue
AB593672.1 Dendrobium suzukii
AB847644.1 Dendrobium bellatulum
KJ672693.1 Dendrobium sinense
MK522249.1 Dendrobium sinense
KJ210498.1 Dendrobium sinense
99
KJ210497.1 Dendrobium sinense
50
KF143511.1 Dendrobium sinense
KF143510.1 Dendrobium sinense
JN388578.1 Dendrobium sinense
KY966521.1 Dendrobium christyanum
MK522207.1 Dendrobium christyanum
AB972353.1 Dendrobium scabrilingue
AB972351.1 Dendrobium christyanum
KJ672656.1 Dendrobium christyanum
KJ210426.1 Dendrobium christyanum
KJ210425.1 Dendrobium christyanum
KF143442.1 Dendrobium christyanum
KF143441.1 Dendrobium christyanum
GU339106.1 Dendrobium christyanum
EF629325.1 Dendrobium christyanum

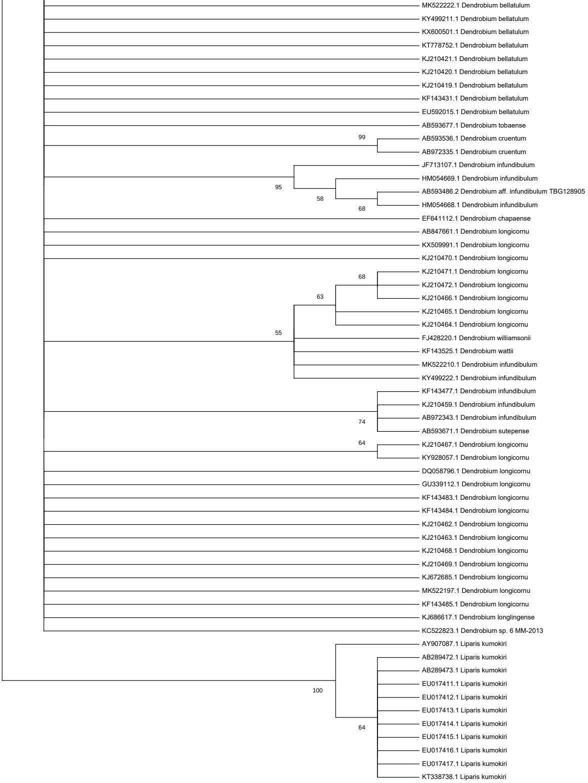
MK522222.1 Dendrobium bellatulum
KY499211.1 Dendrobium bellatulum
KX600501.1 Dendrobium bellatulum
KT778752.1 Dendrobium bellatulum
KJ210421.1 Dendrobium bellatulum
KJ210420.1 Dendrobium bellatulum
KJ210419.1 Dendrobium bellatulum
KF143431.1 Dendrobium bellatulum
EU592015.1 Dendrobium bellatulum
AB593677.1 Dendrobium tobaense
99
AB593536.1 Dendrobium cruentum
AB972335.1 Dendrobium cruentum
JF713107.1 Dendrobium infundibulum
HM054669.1 Dendrobium infundibulum
95
AB593486.2 Dendrobium aff. infundibulum TBG128905
58
HM054668.1 Dendrobium infundibulum
68
EF641112.1 Dendrobium chapaense
AB847661.1 Dendrobium longicornu
KX509991.1 Dendrobium longicornu
KJ210470.1 Dendrobium longicornu
68
KJ210471.1 Dendrobium longicornu
KJ210472.1 Dendrobium longicornu
63
KJ210466.1 Dendrobium longicornu
KJ210465.1 Dendrobium longicornu
KJ210464.1 Dendrobium longicornu
55
FJ428220.1 Dendrobium williamsonii
KF143525.1 Dendrobium wattii
MK522210.1 Dendrobium infundibulum
KY499222.1 Dendrobium infundibulum
KF143477.1 Dendrobium infundibulum
KJ210459.1 Dendrobium infundibulum
AB972343.1 Dendrobium infundibulum
74
AB593671.1 Dendrobium sutepense
64
KJ210467.1 Dendrobium longicornu
KY928057.1 Dendrobium longicornu
DQ058796.1 Dendrobium longicornu
GU339112.1 Dendrobium longicornu
KF143483.1 Dendrobium longicornu
KF143484.1 Dendrobium longicornu
KJ210462.1 Dendrobium longicornu
KJ210463.1 Dendrobium longicornu
KJ210468.1 Dendrobium longicornu
KJ210469.1 Dendrobium longicornu
KJ672685.1 Dendrobium longicornu
MK522197.1 Dendrobium longicornu
KF143485.1 Dendrobium longicornu
KJ686617.1 Dendrobium longlingense
KC522823.1 Dendrobium sp. 6 MM-2013
AY907087.1 Liparis kumokiri
AB289472.1 Liparis kumokiri
AB289473.1 Liparis kumokiri
EU017411.1 Liparis kumokiri
100
EU017412.1 Liparis kumokiri
EU017413.1 Liparis kumokiri
EU017414.1 Liparis kumokiri
64
EU017415.1 Liparis kumokiri
EU017416.1 Liparis kumokiri
EU017417.1 Liparis kumokiri
KT338738.1 Liparis kumokiri
